# Accurate de novo design of high-affinity protein-binding macrocycles using deep learning

**DOI:** 10.1038/s41589-025-01929-w

**Published:** 2025-06-20

**Authors:** Stephen A. Rettie, David Juergens, Victor Adebomi, Yensi Flores Bueso, Qinqin Zhao, Alexandria N. Leveille, Andi Liu, Asim K. Bera, Joana A. Wilms, Alina Üffing, Alex Kang, Evans Brackenbrough, Mila Lamb, Stacey R. Gerben, Analisa Murray, Paul M. Levine, Maika Schneider, Vibha Vasireddy, Sergey Ovchinnikov, Oliver H. Weiergräber, Dieter Willbold, Joshua A. Kritzer, Joseph D. Mougous, David Baker, Frank DiMaio, Gaurav Bhardwaj

**Affiliations:** 1 Department of Medicinal Chemistry, University of Washington, Seattle, WA USA; 2 Institute for Protein Design, University of Washington, Seattle, WA USA; 3 Molecular and Cellular Biology Program, University of Washington, Seattle, WA USA; 4 Graduate Program in Molecular Engineering, University of Washington, Seattle, WA USA; 5 Department of Biochemistry, University of Washington, Seattle, WA USA; 6 Cancer Research @UCC, University College Cork, Cork, Ireland; 7 Department of Microbiology, University of Washington, Seattle, WA USA; 8 Department of Chemistry, Tufts University, 62 Talbot Avenue, Medford, MA USA; 9 Heinrich–Heine–Universität Düsseldorf, Institut für Physikalische Biologie, Düsseldorf, Germany; 10 Forschungszentrum Jülich, Institute of Biological Information Processing, Structural Biochemistry (IBI–7), Jülich, Germany; 11 Department of Chemistry, University of Washington, Seattle, WA USA; 12 Department of Biology, Massachusetts Institute of Technology, Cambridge, MA USA; 13 Howard Hughes Medical Institute, University of Washington, Seattle, WA USA; 14 Present Address: Molecular Cell Biology of Autophagy Laboratory, The Francis Crick Institute, London, UK

**Keywords:** Peptides, Protein design, X-ray crystallography, Machine learning

## Abstract

Developing macrocyclic binders to therapeutic proteins typically relies on large-scale screening methods that are resource intensive and provide little control over binding mode. Despite progress in protein design, there are currently no robust approaches for de novo design of protein-binding macrocycles. Here we introduce RFpeptides, a denoising diffusion-based pipeline for designing macrocyclic binders against protein targets of interest. We tested 20 or fewer designed macrocycles against each of four diverse proteins and obtained binders with medium to high affinity against all targets. For one of the targets, Rhombotarget A (RbtA), we designed a high-affinity binder (*K*_d_ < 10 nM) despite starting from the predicted target structure. X-ray structures for macrocycle-bound myeloid cell leukemia 1, γ-aminobutyric acid type A receptor-associated protein and RbtA complexes match closely with the computational models, with a Cα root-mean-square deviation < 1.5 Å to the design models. RFpeptides provides a framework for rapid and custom design of macrocyclic peptides for diagnostic and therapeutic applications.

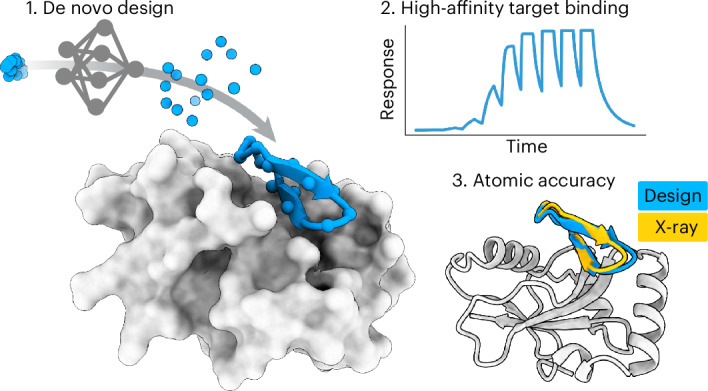

## Abstract (web summary)

A method for de novo design of peptide macrocyles called RFpeptides has been developed. RFpeptides is an extension of RoseTTAFold2 and RFdiffusion and combines structure prediction and protein backbone generation for rapid and custom design of macrocyclic peptide binders.

## Main

Macrocyclic peptides present a promising avenue for developing new therapeutics that bridge the gap between small-molecule drugs and large biologics^1,2^. Biologics, while capable of binding diverse therapeutic targets with high affinity and selectivity, are usually unable to cross cell membranes because of their large size and high polarity, limiting them to extracellular targets. Conversely, small molecules can access intracellular targets but are not ideal for targeting proteins lacking deep hydrophobic pockets. In principle, macrocyclic peptides with sizes between small molecules and proteins can be developed to modulate molecular targets inaccessible to traditional therapeutic modalities^3^. The ability to develop custom protein-binding macrocycles for diverse protein targets would have many diagnostic and therapeutic applications. Traditionally, the development of peptide therapeutics has relied on natural product discovery or high-throughput screening of trillions of random peptides for target binding using display-based techniques^1,2^. However, natural product discovery has several challenges, particularly synthetic difficulties, marginal stability and low mutational tolerance of identified hits^4^. While powerful, the high-throughput screening methods are time-intensive, cost-intensive and labor-intensive and only span a small fraction of the rich chemical and structural diversity accessible to macrocycles. Moreover, such approaches frequently fail to simultaneously optimize for multiple biophysical properties, such as target binding, selectivity and membrane permeability, because of the precise structural control required to achieve such functional properties^5^.

Structure-guided design methods offer a complementary approach to the library screening approaches, enabling rapid in silico exploration of a large chemical and structural diversity to design macrocycle binders for therapeutic targets. We previously developed physics-based methods for designing hyperstable constrained peptides, structured macrocycles and binders to protein targets by borrowing the motifs or interactions from previously described binding partners as anchors^6–9^. However, despite the high accuracy observed in the design of monomeric macrocycles with these methods^7^, the design of protein-binding macrocycles has had limited success, achieving only modest binding affinities and, in many cases, with the experimentally determined structures not agreeing with the design models^7,8,10^. The reliance on previously described binding partners for starting motifs also restricts such approaches to well-studied protein targets. In recent work, we described a pipeline for hallucinating and predicting the structures of macrocyclic peptide monomers by modifying AlphaFold2 (AF2) to include cyclic relative positional encoding (named ‘AfCycDesign’)^11^. Other promising deep learning (DL) methods were described recently to predict the structures of macrocycles and macrocycle–target complexes^12,13^ and to design peptide binders to protein targets^14–16^. However, these methods have not been extensively structurally validated to date or shown to robustly perform atomically accurate de novo design of macrocyclic peptide structures in complexes with diverse protein targets. Computational methods that can accurately design high-affinity macrocycle binders de novo, using just the information of target structure or sequence, are required for wider therapeutic applications.

We reasoned that recent breakthroughs in generative DL methods could be leveraged to develop a robust pipeline for the accurate and efficient design of macrocycle binders. Diffusion models for protein design, such as RFdiffusion^17^, are trained to generate diverse protein structures from randomly initialized residues as starting points and have demonstrated remarkable success in designing protein monomers, binders and symmetric oligomers of medium-sized to large-sized proteins. However, despite considerable recent progress in DL-based protein design methods, these methods are not readily applicable to designing macrocyclic peptides. Developing analogous methods for peptide design from scratch has been challenging because of the limited availability of experimental data for training such models. To address these challenges, we set out to extend the RoseTTAFold2 (RF2)^18^ structure prediction network and the RFdiffusion^17^ protein backbone generation framework to incorporate cyclic relative positional encoding and enable the generation of the macrocyclic peptide backbones.

## Extending RF2 and RFdiffusion for macrocycles

**Fig. 1:**
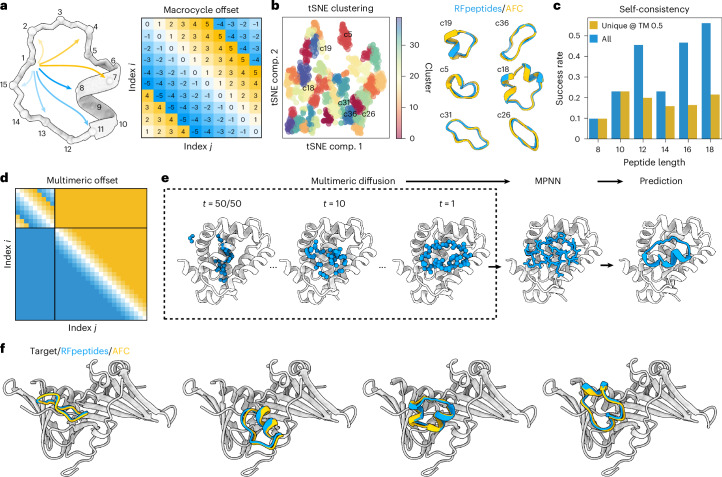
RFpeptides is a diffusion-based pipeline for the de novo design of protein-binding macrocycles. **a**, Cyclically symmetric relative position encoding enables the generation of macrocyclic peptide backbones with N and C termini linked by a peptide bond. The relative position encodings are cyclized by switching from positive relative position encodings (that is, to the right) to negative encodings (that is, to the left) when index *j* is more than halfway around the peptide relative to index *i*. **b**, RFpeptides produces diverse and designable cyclic peptides. Left: structural clusters calculated using *t*-distributed stochastic neighbor embedding (tSNE)^31,32^ to reduce the dimensionality of an all-by-all TMscore matrix computed with TMalign^33^ on an unfiltered set of 1,200 macrocycles generated using RFpeptides. Right: six RFpeptides outputs from differing structural clusters, all with <1 Å backbone r.m.s.d. between the design model (blue) and the structure predicted by AfCycDesign (gold). comp., component. **c**, Self-consistency of designed macrocycles of various lengths. For each peptide length, the fraction (with *n* = 200 per length) of backbones with at least one of the of eight LigandMPNN^34^ sequences predicted by AfCycDesign to refold with pLDDT > 0.8 and within 2.0 Å backbone r.m.s.d. of the designed structure. Success rates for all sampled backbones are in blue and success rates only counting unique structural clusters (as calculated using MaxCluster^35,36^ at a TMscore threshold of 0.5) are in orange. **d**, For multichain diffusion trajectories (for example, macrocycle binder design), the relative positional encoding for the macrocycle chain is cyclized, whereas interchain and target chain relative positional encoding is kept as standard. **e**, Pipeline for the design of protein-binding macrocycles using RFpeptides. Macrocycle backbones are generated from randomly initialized atoms by a stepwise RFdiffusion-based denoising process, followed by amino acid sequence design using ProteinMPNN. Design models are downselected on the basis of the computational metrics from structure prediction using AfCycDesign and physics-based interface quality metrics using Rosetta. **f**, RFpeptides generates diverse macrocycles against selected targets. Four diverse cyclic peptide binders against the same target were generated using RFpeptides, with AfCycDesign iPAE < 0.3 and Cα r.m.s.d. < 1.5 Å between the design model (blue) and AfCycDesign prediction (gold). Source data

We began by examining the ability of the RF2 (ref. ^18^) structure prediction network to model known macrocyclic peptide structures. We implemented a modified (Methods) cyclic relative position encoding for RF2 (Fig. 1a) and observed robust prediction of natural cyclic peptide structures (Supplementary Fig. 1). Given this success, we reasoned that the same relative positional encoding should enable RFdiffusion^17^ to generate macrocyclic peptide structures because of its similar network architecture. We added the cyclic positional encoding scheme to RFdiffusion and observed robust generation of diverse macrocyclic peptides (Fig. 1b,c and Supplementary Fig. 2). Similar to the previously described work on designing monomeric cyclic peptides with physics-based methods^7^ and AfCycDesign^11^, we observed 9,045 and 8,913 structurally unique 10-residue and 12-residue backbones, respectively, when 48,000 macrocycle backbones were generated for each size (Supplementary Fig. 2). The distribution of phi and psi values in these generated backbones is similar to the standard Ramachandran plot for protein structures (Supplementary Fig. 2), suggesting that generated backbones do not require extensive d-amino acids to stabilize the generated structures^7^. While we did not attempt to comprehensively enumerate the structural space of cyclic peptide monomers, RFpeptides can readily be scaled up to comprehensively cover the structural space accessible to macrocyclic peptides. Encouraged by the transferability of the cyclic positional encoding, we set out to use RFdiffusion for the de novo design of protein-binding macrocycles. We chose RFdiffusion for several reasons. Firstly, we expected the high experimental success rate of RFdiffusion^17,19^ for protein binder design to carry over to macrocycle binder design. Secondly, de novo binder design with AfCycDesign as is would be far more computationally expensive and has not been successfully implemented or experimentally validated. Thirdly, the method can still take advantage of the current built-in conditional generation functionalities of RFdiffusion, such as epitope-specific targeting and ‘motif’ scaffolding. Lastly, the method should be directly transferable to other current and future RoseTTAFold-based design networks, such as RFdiffusion All-Atom^20^, for incorporating nonpeptidic molecules (nucleic acids, ions, etc.) during design calculations.

We modified the RFdiffusion protein binder design pipeline to use cyclic relative position encodings for the generated chain and standard positional encodings for the target and interbinder target indices (Fig. 1d). We then completed our design pipeline by using ProteinMPNN^21^ to design amino acid sequences compatible with the backbones generated by RFdiffusion (Fig. 1e). We chose ProteinMPNN for its improved performance in sequence design and ability to generate sequences with better solubility profiles over the sequences generated by traditional physics-based methods^22^. This pipeline readily generated macrocycles with diverse secondary structure content against target proteins (Fig. 1f) and the inclusion of standard RFdiffusion hotspot features clearly shifted the distribution of generated binders toward desired residues (Supplementary Fig. 3). We refer to this integrated pipeline as ‘RFpeptides’ throughout the remainder of the text.

## De novo design of macrocyclic binders to myeloid cell leukemia 1 and MDM2

We selected myeloid cell leukemia 1 (MCL1) as our first target protein, given the availability of multiple high-resolution X-ray crystal structures available to initiate the design calculations. MCL1 is also a promising target for anticancer therapeutics because of its roles in autophagy, cell survival, DNA repair and cellular proliferation^23^. For targeting MCL1, we used RFpeptides to generate 9,965 diverse cyclic peptide backbones, followed by four iterative rounds of ProteinMPNN and Rosetta Relax to design four amino acid sequences for each generated backbone. We expected the local changes to the generated backbone during the Rosetta Relax steps to allow for improved amino acid sequence diversity from the ProteinMPNN steps. While there are other ways to achieve increased sequence diversity, including generating multiple sequences per backbone from ProteinMPNN or adding noise during ProteinMPNN sequence generation, we did not explicitly try or compare them in this study. For downselecting the design candidates for experimental testing, we used AfCycDesign to repredict the designed macrocycle–target complexes from the macrocycle sequence and the target structure as a template. We selected the designs on the basis of the confidence metric (interface predicted aligned error (iPAE)) and the similarity between the original design model and the protein–macrocycle complex predicted by the AfCycDesign (Supplementary Fig. 4). For further stringency in the design selection process, we also used RF2 to repredict the complex structures, reasoning that the design models predicted identically by two orthogonal structure prediction networks (AfCycDesign and RF2) should have a higher likelihood of binding to the target as designed. However, the 1,984 selected designs at this stage were still more than the number of designs we could reasonably synthesize and test experimentally. Therefore, we next used Rosetta^24^ to calculate the ‘physics-based’ metrics of interface and macrocycle quality, such as calculated binding affinity (ddG), spatial aggregation propensity (SAP) of the designed macrocycle and the molecular surface area of the interface contacts (CMS) (Supplementary Fig. 4).

**Fig. 2:**
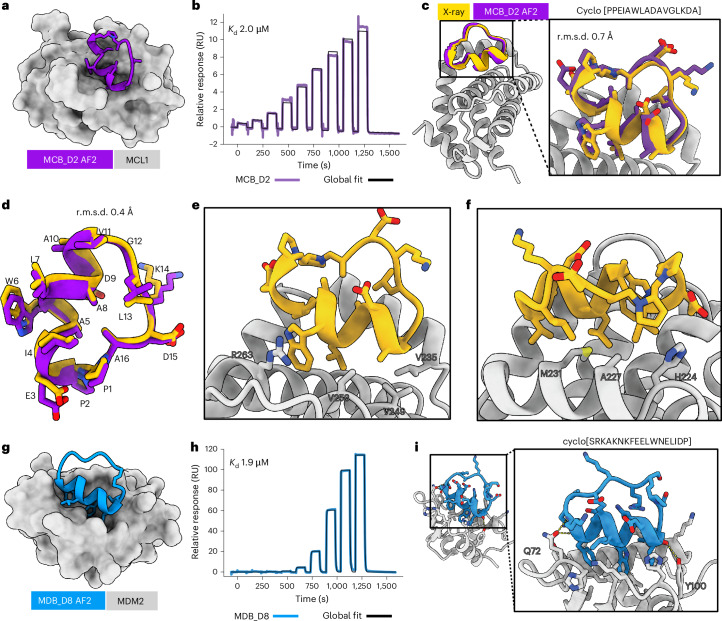
De novo design and characterization of macrocyclic binders to MCL1 and MDM2. **a**, AfCycDesign prediction of MCB_D2 (purple) bound to MCL1 (gray surface). MCB_D2 side chains are shown as sticks. **b**, Affinity determination of MCB_D2 using SPR. SPR sensorgram from a nine-point single-cycle kinetics experiment (twofold dilution, highest concentration: 20 µM). Experimental data are shown in purple and global fits are shown with black lines. The *K*_d_ is also shown on the plot. **c**, Experimentally determined complex structures closely match the design model. Overlap of the X-ray crystal structure (gold and gray) with the design model for MCB_D2 (purple). The Cα r.m.s.d. for the macrocycle is 0.7 Å when the experimental structure and design models are aligned by MCL1 residues. Close-up views demonstrate strong agreement between the side-chain rotamers of the design model and the X-ray structure. **d**, Overlay of the macrocycle model to the crystal structure shows a Cα r.m.s.d. of 0.4 Å with nearly identical backbones and side-chain rotamers. **e**, Close-up view of the macrocycle-bound MCL1 structure showing the cation–π interaction at the interface. **f**, Close-up view of the macrocycle-bound MCL1 structure showing the hydrophobic contacts at the interface. **g**, AfCycDesign prediction of MDB_D8 design (blue) in complex with MDM2 (gray) shown as cartoons with interacting side chains shown as sticks, bound to MDM2 shown as surface. **h**, Affinity determination of MDB_D8 using SPR. SPR sensorgram from a nine-point single-cycle kinetics experiment (fivefold dilution, highest concentration: 50 µM). Experimental data are shown in blue and global fits are shown with black lines. The *K*_d_ is also shown on the plots. **i**, Overall and close-up views of the AfCycDesign prediction of the MDB_D8 design model, highlighting key interactions with the MDM2. Source data

After strictly filtering the designed candidates on DL-based and physics-based metrics, we selected 27 designs for synthesis, biochemical and biophysical characterization. Despite specifying no hotspots to guide the generation process to a specific patch on the MCL1 structure, all selected designs bound to the functionally relevant MCL1–BH3 interaction site (Supplementary Fig. 5). While all selected designs include an α-helical segment, they feature different sequences, macrocycle placement and target interactions (Supplementary Fig. 5 and Supplementary Table 1a). In addition to the common helical motifs, the loop regions of the selected macrocycles also contribute extensive side-chain-mediated and backbone-mediated interactions to the binding interface. During the chemical synthesis using Fmoc-based solid-phase synthesis (Methods), the yields for the correctly cyclized product for 13 designs were low and insufficient for further characterization. We tested the remaining 14 macrocycles for binding to biotinylated MCL1 using surface plasmon resonance (SPR) single-cycle kinetics experiments (Supplementary Fig. 6). Three macrocycles showed binding to the MCL1, with the best binder, MCB_D2 (MCL1 binding design 2) (Fig. 2a), demonstrating a binding affinity of 2 µM (Fig. 2b). To confirm whether the designed macrocycle adopts the designed structure and engages MCL1 in the designed binding mode, we determined the X-ray crystal structure of MCB_D2 bound to MCL1 at 2.1 Å resolution. The crystal structure was nearly identical to the design model, with a root-mean-square deviation (r.m.s.d.) of 0.7 Å over all of the Cα atoms of the macrocycle with target chains aligned (Fig. 2c) and Cα r.m.s.d. of 0.4 Å within the macrocycles when aligned (Fig. 2d). The side-chain rotamers of the interacting residues in the crystal structure also closely matched the design model (Fig. 2d). The crystal structure also confirmed that the binding interactions are not restricted to the helix region of the designed macrocycle but are also contributed by the loop regions (Fig. 2e,f). While several hydrophobic interactions from the MCB_D2 helical segment are similar to those seen in natural MCL1 binders (for example, BH3 peptide), (Supplementary Fig. 7), the N-to-C orientation of the helix is flipped in the case of MCB_D2. The loop region of MCB_D2 makes additional hydrophobic contacts and a cation–π interaction with MCL1 (Fig. 2e and Supplementary Fig. 7d) that we did not observe in previously reported natural MCL1 binders and their analogs. All three hits with an observable binding signal at 100 µM featured this cation–π interaction.

Encouraged by the experimental validation of the MCL1 binding macrocycles, we next sought to design binders to MDM2, an E3 ligase that interacts with tumor suppressor protein p53 and has multiple critical roles in tumor growth and survival^25^. We generated 10,000 macrocycle backbones spanning diverse lengths amenable to chemical synthesis (16–18 residues) and designed four amino acid sequences for each generated backbone using iterative rounds of ProteinMPNN and Rosetta Relax protocols (Methods). Design models were filtered on the basis of the confidence metrics and similarity of the AfCycDesign predictions to the designed complexes and the interface quality metrics calculated using Rosetta (Supplementary Fig. 4). AfCycDesign predicted 7,495 of the 40,000 design models to bind MDM2 with high confidence (normalized iPAE < 0.3) (Supplementary Fig. 4). In contrast to our approach for MCL1, we chose not to do any additional filtering with RF2 as the results between AfCycDesign and RF2 were fairly consistent. We also adjusted the filter thresholds for in silico filters as their overall distribution differed substantially from the distribution observed for MCL1 (Supplementary Fig. 4). After filtering on interface metrics (Methods), we identified 17 designs with iPAE < 0.3, ddG < −50 kcal mol^−1^, CMS > 300 Å^2^ and SAP < 35. We selected 11 top-ranked designs by ddG for biochemical and biophysical characterization. The 11 selected designs had diverse sizes, shapes and sequences (Supplementary Fig. 8 and Supplementary Table 2); however, they were all predicted to bind the same site as the p53 transactivation domain (Supplementary Fig. 8). Three of the selected designs had poor yields during the cyclization step of the chemical synthesis, preventing further experimental characterization with them. We tested the remaining eight peptides for binding to the biotinylated MDM2 by SPR and identified three binders with observable binding signals at 100 µM (Supplementary Fig. 9). The best design, MDB_D8 (Fig. 2g), demonstrated a binding affinity of 1.9 µM in the SPR single-cycle kinetics experiment (Fig. 2h). The computational model for this design makes several key contacts at the interface that are similar to interactions observed in native MDM2–p53 complex structures (Fig. 2i and Supplementary Fig. 10)^25^. Despite different overall structures, all three hits from the SPR screen had a similar binding motif composed of phenylalanine, tryptophan and either leucine or methionine from the helical segment of the macrocycle. Together, these data highlight the promising accuracy of the RFpeptides pipeline to design diverse macrocyclic binders for selected targets of interest.

## De novo design of macrocyclic binders to γ-aminobutyric acid type A receptor-associated protein

**Fig. 3:**
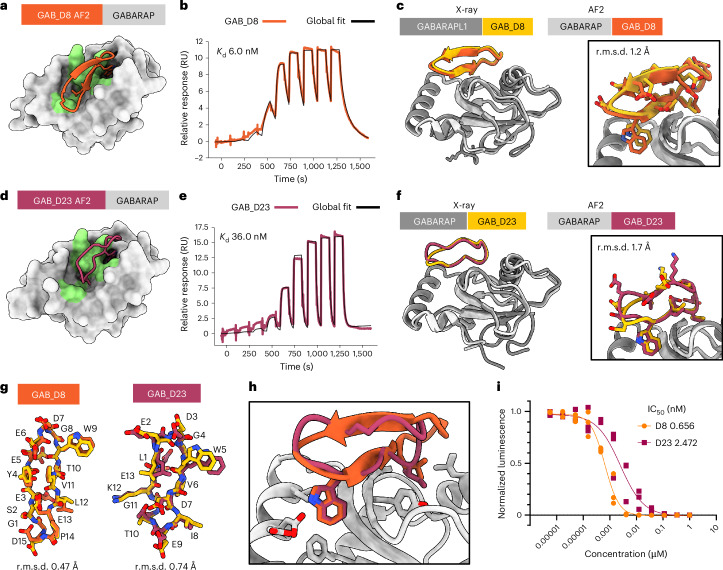
De novo design of high-affinity macrocycle binders to GABARAP. **a**, AfCycDesign predicted model for design GAB_D8 bound to GABARAP shown as surface, with hotspot residues highlighted in green. **b**, Affinity determination of GAB_D8 using SPR. SPR sensorgram from a nine-point single-cycle kinetics experiment (fivefold dilution, highest concentration: 20 µM). Experimental data are shown in orange and global fits are shown with black lines. The *K*_d_ is also shown on the plot. **c**, Superposition of chains E and F from the X-ray crystal structure of GAB_D8 bound to GABARAPL1 and the AfCycDesign model. **d**, AfCycDesign predicted model for design GAB_D23 bound to GABARAP shown as surface, with hotspot residues highlighted in green. **e**, Affinity determination of GAB_D23 using SPR. SPR sensorgram from a nine-point single-cycle kinetics experiment (fivefold dilution, highest concentration: 20 µM). Experimental data are shown in pink and global fits are shown with black lines. The *K*_d_ is also shown on the plot. **f**, Alignment of chains A and B from the X-ray crystal structure of GAB_D23 bound to GABARAP and the AfCycDesign model. **g**, Alignments of GAB_D8 and GAB_D23 macrocycle models to X-ray crystal structures show close matches. **h**, Comparison of GAB_D8 and GAB_D23 binding modes in the design models. **i**, Competitive AlphaScreen dose-response plot, IC_50_ from the average of three experiments. Donor and acceptor beads in the assay are bound to GABARAP and GABARAP-binding peptide K1, respectively. Source data

We next set out to design binders against a target with a binding site that is structurally different from MCL1 and MDM2, formed by a mix of α-helices and β-strands (in contrast to all α-helical pockets of MCL1 and MDM2). We selected γ-aminobutyric acid type A receptor-associated (GABARAP) as the target, a protein responsible for mediating autophagy through its role in autophagosome biogenesis and recruitment of cargo, resulting in lysosomal degradation of damaged or surplus proteins and organelles^26^. Peptide modulators against GABARAP could have therapeutic applications in the treatment of late-stage cancers^27^ or as chimeric peptides for autophagy-mediated targeted protein degradation^28^. Our target binding site for GABARAP, which is also the binding site for the native LC3-interacting region or Atg8-interacting motif^29^, is formed by a mix of β-strand and α-helix secondary structures (Fig. 3 and Supplementary Fig. 13). For designing macrocyclic binders against the human GABARAP, we used a similar pipeline as described above for MCL1 and MDM2 (Methods) but we doubled the number of generated designs and defined six hotspot residues (Lys46, Lys48, Tyr49, Leu50, Phe60 and Leu63) to guide the macrocycle backbone generation to a specific site on the target (Fig. 3a,d). We generated 20,000 macrocycle backbones and designed the amino acid sequences using ProteinMPNN and Rosetta Relax protocols. Of the resulting 80,000 design models, we selected 335 macrocyclic designs on the basis of AfCycDesign (iPAE < 0.13) and Rosetta (ddG < −30 kcal mol^−1^, SAP < 35 and CMS > 300 Å^2^) interface metrics (Supplementary Fig. 4). Instead of trying to synthesize and characterize all 335 cyclic peptides (which would have required substantial time and experimental resources), we clustered the 335 designs into 80 different clusters on the basis of their three-dimensional structures and selected representative designs from diverse clusters for further biochemical characterization. We selected 13 diverse macrocycles of 12–17 residues for synthesis and experimental validation (Supplementary Table 3 and Supplementary Fig. 11). Unlike the design candidates described above for MCL1 and MDM2, several of the selected macrocycles for GABARAP showed cyclic β-sheet structures with several edge–strand interactions with the target (Supplementary Fig. 11).

We successfully synthesized six designs with high purity (>90%) and tested them for binding to GABARAP using SPR (Supplementary Fig. 12). Two designs, GAB_D8 and GAB_D23, showed binding affinities of 6 nM and 36 nM, respectively (Fig. 3b,e). To further characterize the binding of GAB_D8 and GAB_D23, we tested the ability of these designs to disrupt the interaction of GABARAP with linear peptide K1 (a previously described binder to this site^30^) in AlphaScreen assays. GAB_D8 and GAB_D23 demonstrated a half-maximal inhibitory concentration (IC_50_) of 0.7 nM and 2.5 nM in the AlphaScreen assay, respectively (Fig. 3i). To our knowledge, GAB_D8 is the most potent macrocyclic GABARAP binder to date.

In crystallization trials, we did not obtain crystals of sufficiently high quality for GAB_D8 bound to GABARAP. We instead crystallized GAB_D8 bound to GABARAPL1, a homolog of GABARAP with 86% overall sequence identity and 100% sequence identity for residues within 5 Å of GAB_D8 in the design model. The X-ray crystal structure for GAB_D8 bound to GABARAPL1 matched very closely with the design model, with a Cα r.m.s.d. of 1.2 Å over the macrocycle when aligned by the target protein to the closest of the four copies in the asymmetric unit (Fig. 3c and Supplementary Fig. 13) and a Cα r.m.s.d. of 0.47 Å when aligned by macrocycle alone (Fig. 3g). Notably, the X-ray structure of the GAB_D8–GABARAPL1 complex showed two different bound conformations of GAB_D8, one that closely matched the design model and a second one that partially deviated from the design model (Supplementary Fig. 14), with a register shift nucleated by Thr10 from the macrocycle forming main-chain-mediated and side-chain-mediated hydrogen bonds with Lys48 on the target. GAB_D23 crystallized readily with GABARAP and also closely matched the design model with a Cα r.m.s.d. of 1.7 Å when aligned by the target (Fig. 3f) and Cα r.m.s.d. of 0.74 Å across the macrocycle alone (Fig. 3g). The X-ray crystal structure confirmed the key designed interactions, such as Trp5 and Ile8, with the main difference between the design model and the X-ray structure being the switch from a type I β-turn from Leu1 to Gly4 in the design model to a less regular conformation in the crystal structure, with a tendency for a type I′ β-turn from Glu2 to Trp5. While our original design models were predicted with single sequences as inputs to AF2, we retrospectively predicted the GAB_D8–GABARAPL1 and GAB_D23–GABARAP complex structures with multiple-sequence alignment (MSA) inputs. These MSA-based predictions of the designs matched even more closely with the X-ray crystal structures, with a Cα r.m.s.d. of 0.5 Å and 0.9 Å for the GAB_D8–GABARAPL1 and GAB_D23–GABARAP complexes, respectively, when aligned by the target structure (Supplementary Fig. 15). Overall, these data demonstrate the ability of our de novo design pipeline to identify high-affinity binders against targets with diverse pocket shapes and surfaces without requiring library-scale screening.

## Design of macrocyclic binders to predicted structures

**Fig. 4:**
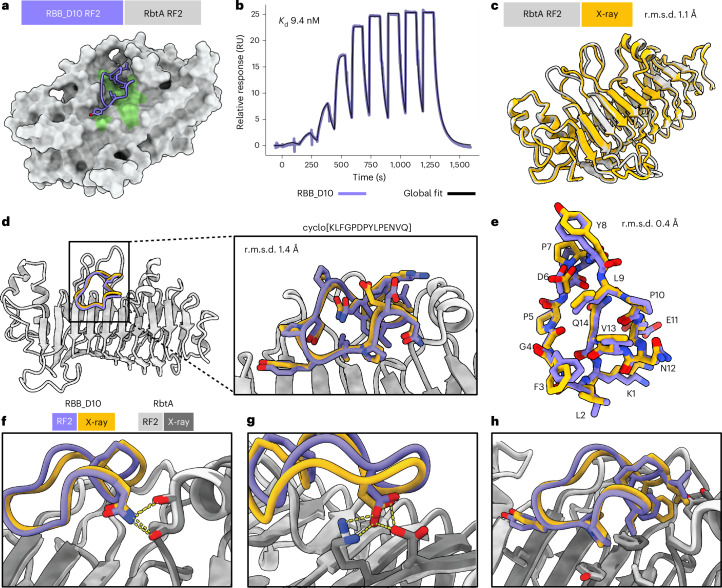
Accurate de novo design of a high-affinity cyclic peptide binder against the predicted structure of RbtA from *A.* *baumannii.* **a**, AfCycDesign prediction of design RBB_D10 (violet cartoon) bound to the AF2-predicted β-helix domain of RbtA shown as gray surface. Hotspot residues from RbtA used during the backbone design step are shown in green. **b**, SPR sensorgram from nine-point single-cycle kinetics experiment (fivefold dilution, highest concentration: 20 µM). The *K*_d_ determined from the SPR experiment is also denoted on the plot. **c**, Close agreement of the RF2-predicted structure of RbtA (gray) with the X-ray structure (gold) of the RbtA N-terminal domain determined here confirms the predicted structure of the target used for the macrocycle design calculations. **d**, Alignment of the design model of RbtA-bound RBB_D10 (violet and gray) to the X-ray structure (gold) shows a close match between the design model and the experimentally determined structure (Cɑ r.m.s.d. for macrocycle: 1.4 Å). Close-up view of the RbtA-bound RBB_D10 with side chains shown as sticks. **e**, Overlay of RBB_D10 design model (after the AfCycDesign prediction step) aligned to the X-ray structure without RbtA demonstrates a nearly identical match for backbone coordinates and side-chain rotamers (Cɑ r.m.s.d.: 0.4 Å). The design model and X-ray structure are shown in violet and gold, respectively. **f**, Close-up view of the macrocycle-bound RbtA structure and design model showing polar side chain-to-backbone interactions mediated by RBB_D10 residue Asn12 at the interface. **g**, Close-up view of the polar side chain-to-side chain interactions mediated by RBB_D10 residue Asp6 at the interface. **h**, Close-up view of the hydrophobic interactions between RbtA and RBB_D10 at the binding interface. Source data

Given the high accuracy and binding affinity of macrocycles designed against selected targets, we next set out to design macrocyclic binders against targets without any experimentally determined structure. We reasoned that the high accuracy of RFpeptides could mitigate the inherent risk of designing against a predicted target structure. We designed macrocycles against Rhombotarget A (RbtA), a recently identified cell surface protein from the ESKAPE pathogen, *Acinetobacter baumannii*. There are no experimentally determined structures available for this protein and sequence-based searches against the Protein Data Bank (PDB) did not return notable matches to other protein structures. We predicted the structure of the 617-aa full-length protein using AF2 and RF2; both methods predicted similar overall structures (Cα r.m.s.d. of 0.4 Å over 509 residues excluding the signal peptide and transmembrane domain) with high confidence (predicted local distance difference test (pLDDT) > 90) (Supplementary Fig. 16). AF2 and RF2 both predicted two distinct extracellular domains: an N-terminal β-helix domain and a C-terminal Ig-like domain (Supplementary Fig. 16). While there were some differences in the predicted structures from AF2 and RF2, we decided to focus our binder design calculations on regions that were predicted nearly identically and with high confidence by AF2 and RF2. On the basis of our preliminary design runs without hotspots to guide the diffusion, we identified a patch in the N-terminal domain to pursue in our large-scale design calculations against this target and defined hotspots Leu144, Phe202, Phe204, Tyr206, Val208, Leu231 and Ala269 for peptide backbone generation (Fig. 4a). In contrast to the concave pockets targeted for MDM2 and MCL1, this selected patch for RbtA is considerably flatter and difficult to target with conventional computational and experimental approaches (Supplementary Fig. 17). We generated 20,000 backbones for macrocycle binders and designed four amino acid sequences for each backbone using iterative rounds of ProteinMPNN and Rosetta Relax. Designs were filtered using AfCycDesign confidence metrics and Rosetta interface metrics, as described in earlier sections (Supplementary Fig. 4). On the basis of these in silico metrics, we selected 26 designs for biochemical and structural characterization with AfCycDesign iPAE < 0.4, ddG < –30 kcal mol^−1^, r.m.s.d. between the design model and AfCycDesign prediction < 1.5 Å and CMS > 300 Å^2^ (Supplementary Fig. 18). The selected designs covered diverse sizes (13–18 aa), sequences, shapes and secondary structures (Supplementary Fig. 18 and Supplementary Table 4). We expressed the Avi-tagged version of the RbtA N-terminal domain (residues 20–458) and used it for binding screens using SPR. Four of 11 designs that were synthesized in sufficient quantity and purity showed a binding signal at 100 µM in our screens (Supplementary Fig. 19). On the basis of further binding experiments with SPR, we determined the dissociation constant (*K*_d_) of the best binder, RBB_D10, to be 9.4 nM (Fig. 4b). The design model for RBB_D10 showed extensive contacts to the target with several side-chain-mediated polar contacts and hydrophobic interactions (Fig. 4f–h).

To confirm the structures of RbtA and RBB_D10 and the binding mode between them, we determined the high-resolution X-ray crystal structure of apo and macrocycle-bound RbtA using X-ray crystallography at 2 Å and 2.6 Å resolution, respectively. The apo structure of the RbtA N-terminal domain, which is also the first experimentally determined structure from this class of bacterial proteins, matched our AF2 and RF2 predictions for this target very closely, with an overall Cɑ r.m.s.d. of 1.2 Å and 1.1 Å between the X-ray structure of the RbtA N-terminal domain and the AF2-predicted and RF2-predicted structures, respectively (Fig. 4c). The complex structure also confirmed the structure and binding mode of our designed macrocycle, RBB_D10, with the X-ray structure matching the design model with an r.m.s.d. of 1.4 Å (Fig. 4d). Notably, the conformation adopted by the macrocycle in the X-ray structure, including the side-chain rotamers involved in interactions with the target, was almost identical to the design model with an r.m.s.d. of 0.4 Å (Fig. 4e–h). Together, these data highlight the high accuracy and success rates provided by RFpeptides even while designing macrocycles against targets without deep pockets or targets with no known structures.

Overall, these data show that RFpeptides can sample extensive structural and chemical diversity of macrocycles during the backbone and sequence generation steps against selected targets and, finally, select the shapes and sequences ideally suited for binding the target surface or pockets. The highest-affinity binders against each target are also predicted to fold into the bound conformations even in the absence of the target (Supplementary Fig. 20), suggesting that macrocycles are designed to fold into binding-competent conformations. For all four design campaigns described here, selected designs demonstrated good solubility in aqueous buffers despite not imposing any particular sequence constraints related to solubility during the sequence design step using ProteinMPNN^21^. Notably, combining DL-based and physics-based in silico filters helps to select medium to high-affinity binders. However, we note that the distribution of such metrics varies substantially across the four selected targets and adjustments to filtering thresholds were required on the basis of the shape and chemical composition of the target pocket. While in silico metrics enrich well for binders, the relative ranking within the selected designs does not perfectly match the experimental binding affinities. The highest-affinity binders for MDM2 and RbtA had the best or second-best iPAE values among the designs chosen for those targets (Supplementary Tables 2b and 4b); however, the hit peptides against MCL1 and GABARAP were not among the top three ranked designs (Supplementary Tables 1b and 3b). Integration with high-throughput methods in the future should enable testing of more designs and inform absolute threshold values and filtering schemes for the single-shot design of peptide binders to any arbitrary target.

## Discussion

Here, we describe RFpeptides, a generative DL pipeline for precise de novo design of macrocycle binders against a wide range of protein targets. The power of the approach is highlighted by the high affinities (*K*_d_ < 10 nM) of the designed macrocyclic binders to GABARAP and RbtA and the nearly identical X-ray crystal structures and design models of the macrocycle-bound MCL1, GABARAP and RbtA (Cα r.m.s.d. of 0.7 Å, 1.2 Å and 1.4 Å, respectively). The RFpeptides approach offers several advantages over traditional methods. Firstly, the design approach should enable faster and more efficient discovery of macrocyclic binders. Despite testing fewer than 20 designed candidates per target (in contrast to trillions of peptides tested in traditional library-based approaches), we achieved high-affinity binders for two targets without requiring any further experimental optimization; to our knowledge, this is a considerably higher success rate than achieved with any previous method. Secondly, in contrast to the untargeted nature of the random library-based approaches, RFpeptides can be used for designing custom binders to specific patches and sites, as demonstrated for GABARAP and RbtA. Lastly, the atomically accurate nature of the design models enables structure-guided optimization for properties beyond target binding (as well as further increases in affinity), bypassing the bottleneck of complex structure determination, which has hindered the optimization of leads from library screening. Combined with the design principles for membrane traversal, RFpeptides could enable the design of peptides simultaneously optimized for target binding and cell permeability or oral bioavailability.

RFpeptides also has considerable advantages over previous computational peptide design methods. Information on known ligands and/or binding partners is not required to initiate design. RFpeptides can design macrocycles completely de novo from just the structure or sequence (as in the case of RbtA) of the target, enabling design against molecular targets intractable with previous methods. RFpeptides is not limited to generating macrocycles with particular motifs or topologies; the diffusion process generates macrocycles with diverse shapes and sizes and selects the topologies appropriate for the protein being targeted. Among the four targets tested here, binders for MCL1 and MDM2 have helical motifs, binders for GABARAP have a β-sheet topology and binders for RbtA sample looplike conformations that make extensive contacts with the flat surface of this target.

We anticipate that RFpeptides will enable the rapid design of custom macrocyclic binders against a wide range of molecular targets, accelerating efforts to develop peptides for diverse functional applications. With the rapid advances in DL methods and frameworks, including the recent development of all-atom diffusion models, we aim to extend the approach to generative design of macrocycles with noncanonical amino acids, crosslinkers and cyclization chemistries.

## Methods

### Computational methods for cyclic peptide binder design

Macrocyclic peptide monomers and binders were designed with RFpeptides using a three-stage pipeline: backbone generation using RFdiffusion with the cyclic offset applied to the peptide chains, followed by sequence design using ProteinMPNN and, finally, structure prediction of the designed peptide–target complexes using either AfCycDesign and/or RoseTTAFold with the cyclic offset applied to the peptide. Designs were further filtered and downselected using Rosetta metrics and, in some cases, clustered on the basis of Cα r.m.s.d. Detailed computational methods, including example scripts, can be found in Supplementary Section 2.2.

### Peptide synthesis

Macrocyclic peptides described here were either purchased from Wuxi AppTec at greater than 90% purity or synthesized in-house using Fmoc-based solid-phase peptide synthesis. Peptides were typically synthesized on preloaded CTC resin. The resin was swollen in DCM followed by iterative deprotection with 20% piperidine in DMF and coupling with either HBTU (Sigma) or PyAOP (Novabiochem) and DIEA (Sigma). The linear peptides were cleaved from the resin using either 2% TFA in DCM or 20% HFIP (Oakwood Chemical) in DCM. The solvent was removed by rotary evaporation and linear protected peptides were cyclized in either DCM, DMF or a mixture of both depending on the solubility of the peptide, using two equivalents of PyAOP and five equivalents of DIEA overnight. The protecting groups were removed using a cocktail of 95:2.5:2.5, TFA, water and TIPS for 2.5 h. The crude peptides were precipitated using cold diethyl ether. The precipitate containing the crude cyclization reaction was dissolved in a mixture of water and acetonitrile for purification using reverse-phase high-performance liquid chromatography (LC). Peptide identities were confirmed by mass spectrometry (MS). Purities for all synthesized and tested macrocyclic peptides are also summarized in Supplementary Tables 7–10. The mass spectrograms and analytical LC chromatograms for all purified peptides are shown in Supplementary Section 4.

### Protein expression and purification

#### MDM2 and MCL1

The amino acid sequences of MCL1 (PDB 2PQK)^37^ and MDM2 (PDB 4HFZ)^38^ were retrieved from the PDB. The optimized genes were then cloned into a Novogen pRSF-DUET plasmid (Sigma, 71341-3), incorporating a 6xHis-tag at the N terminus, followed by an Avi-tag and a tobacco etch virus (TEV) protease cleavage site. The resulting constructs were codon-optimized for *Escherichia coli* expression and synthesized by Genscript. For propagation, the plasmids were transformed into *E.* *coli* NEBα cells (New England Biolabs, C2987); for protein expression, the plasmids were transformed into *E.* *coli* BL21(DE3) cells (New England Biolabs, C2527). A single sequence-verified colony was cultured in 50 ml of kanamycin (50 µg ml^−1^) selective Luria Broth (LB) medium. This culture was incubated at 37 °C with shaking at 200 rpm for 16 h overnight. Subsequently, 50 units of optical density at 600 nm (OD_600_) of the overnight culture were transferred to 1 L of fresh kanamycin (50 µg ml^−1^) selective LB medium. The culture was grown at 37 °C with shaking at 200 rpm for 2 h (until it reached an OD_600_ of 0.4–0.5), at which point the temperature was decreased to 20 °C. The culture was grown until an OD_600_ of 0.7–0.8; protein expression was induced by adding 1 mM IPTG and the culture was left to grow overnight for 14 h.

Cells were harvested by centrifugation at 5,000*g* for 10 min at 4 °C, resulting in a cell pellet with a density of 5 g L^−1^. The pellet was immediately flash-frozen and stored at −20 °C for later use. For lysis, the pellet was thawed on ice and resuspended in 5 ml of lysis buffer per gram of pellet. This lysis buffer contained 50 mM Tris-HCl, 300 mM NaCl and 10 mM imidazole and was supplemented with 1× BugBuster protein extraction reagent (Sigma-Aldrich, 70921), 200 µg ml^−1^ lysozyme (Sigma-Aldrich, L6876), 25 U per ml benzonase nuclease (Sigma-Aldrich, E8263) and 1× cOmplete EDTA-free protease inhibitor cocktail (Sigma-Aldrich, 11836170001). The buffer was filter-sterilized using a 0.2 µm filter before the addition of benzonase, mixed by inversion and kept on ice until use. Cells were completely resuspended in the lysis buffer using a homogenizer at low speed and incubated for 30 min at room temperature (22–25 °C). Following incubation, the suspension was sonicated using a Q500 Sonicator equipped with a four-tip probe. Sonication was conducted for 2–3 min using pulses of 10–15 s on followed by 10–15 s off at 70% amplitude. The lysate was clarified by centrifugation at 16,000*g* for 20 min.

Ni-NTA agarose resin (Qiagen, 30210) was equilibrated with 20 column volumes (CV) of ultrapure water, followed by 20 CV of equilibration buffer (50 mM Tris-HCl, 300 mM NaCl and 10 mM imidazole). Then, 4 ml of 50% resin suspended in equilibration buffer was used to bind His-tagged proteins from 25 ml of clarified lysate. All immobilized metal affinity chromatography (IMAC) steps were conducted at 4 °C. The lysate–resin mixture was incubated for 60 min on a rotary shaker set to a slow speed. After incubation, the resin was transferred to a 20-ml gravity column and allowed to completely settle. The resin was first washed with 20 CV of wash buffer 1 (20 mM Tris-HCl, 250 mM NaCl, 10 mM imidazole and 5 mM β-mercaptoethanol), followed by another 20 CV of wash buffer 2 (20 mM Tris-HCl, 500 mM NaCl and 35 mM imidazole). The bound proteins were then eluted with 8 ml of elution buffer (20 mM Tris-HCl, 250 mM NaCl, 350 mM imidazole and 2 mM DTT). Aliquots of the eluate were collected and analyzed using SDS–PAGE gels.

The eluate was loaded onto a pre-equilibrated Superdex 75 10/300 GL column (25 mM Tris-HCl, 250 mM NaCl and 2 mM DTT) and run at a flow rate of 0.6 ml min^−1^ using an ÄKTA pure system for size-exclusion chromatography (SEC). Then, 1 ml fractions were collected from the elution volume of 8–16 ml and those corresponding to peaks in the absorbance at 280 nm between an elution volume of 10 and 13 ml were assessed with SDS–PAGE gels. Fractions confirming the expected molecular weight were pooled and concentrated by centrifugation at 4,000*g* for 30 min at 4 °C using Amicon Ultra-4 concentrators with a 3 kDa cutoff (Millipore Sigma, UFC800308) to a final volume of 500 µl. The identity of the eluted proteins were confirmed by MS using an Agilent 6230 LC–MS time-of-flight system.

Verified protein samples were processed for further applications: biotinylation for SPR analysis or tag removal by TEV protease cleavage for crystallography. Biotinylation was performed using the BirA biotin–protein ligase standard reaction kit (Avidity, BirA-500) according to the manufacturer’s recommended conditions. The reaction was carried out at 4 °C overnight on a slowly shaking platform. For TEV protease cleavage, the proteins were treated with a 25:1 protein to TEVd enzyme ratio^39^. Similarly, the mixture was incubated at 4 °C overnight on a slowly shaking platform. Following these treatments, samples underwent a cleanup step using 1 ml of Ni-NTA resin per 20 mg of protein. The resin was pre-equilibrated with 10 CV of ultrapure water and 10 CV of a buffer containing 25 mM Tris-HCl, 250 mM NaCl and 10 mM imidazole. The pre-equilibrated resin was added to the protein mixture and incubated for 30 min on a rolling platform at 4 °C. Subsequently, the mixtures were filtered through a 0.45 µm PVDF centrifugal filtering unit to remove the Ni-NTA-bound substrates. The eluate was collected and dialyzed in 2 L of 25 mM Tris-HCl, 250 mM NaCl and 2 mM DTT using a Slide-A-Lyzer G3 dialysis cassettes with a 3.5 kDa molecular weight cutoff (Thermo Scientific, A52966) overnight for 18 h at 4 °C stirring. The dialyzed protein was concentrated to 0.2–0.5 ml (as required for downstream assays), using the Amicon ultra concentrators (as above), aliquoted and flash-frozen. Fractions were analyzed by mass spectroscopy for the efficacy of the biotinylation and TEV protease cleavage treatments, as previously described.

#### GABARAP for SPR

A synthetic complementary DNA was designed on the basis of the amino acid sequence of GABARAP (UniProt O95166) and optimized for expression in *E.* *coli* using Benchling software. The construct was devised to include an N-terminal Avi-tag and TEV protease cleavage site and was cloned into the Novogen pET-50b(+) plasmid. This plasmid configuration introduced a tandem arrangement of protein tags at the N terminus: a 6xHis-tag, followed by a NusA solubility tag, another 6xHis-tag and a human rhinovirus (HRV) 3C protease cleavage site. Therefore, the final construct sequence was as follows: 6xHis–NusA–6xHis–HRV 3C–Avi–TEV–GABARAP. NusA was specifically chosen as a solubility tag because of its known effectiveness in enhancing protein solubility in *E.* *coli*^40,41^. The construct was synthesized and cloned by Genscript.

As described above for MCL1 and MDM2 protein expression, the plasmids were introduced into *E.* *coli* NEBα cells and BL21(DE3) cells. A single sequence-verified colony was cultured in 50 ml of kanamycin (50 µg ml^−1^) selective LB medium for 16 h at 37 °C, shaking at 200 rpm. Then, 50 OD_600_ units of this culture were transferred to 1 L of fresh kanamycin (100 µg ml^−1^) selective autoinduction medium (TBM-5052: 1.2% (w/v) tryptone, 2.4% (w/v) yeast extract, 0.5% (v/v) glycerol, 0.05% (w/v) d-glucose, 0.2% (w/v) d-lactose, 25 mM Na_2_HPO_4_, 25 mM KH_2_PO_4_, 50 mM NH_4_Cl, 5 mM Na_2_SO_4_, 2 mM MgSO_4_, 10 μM FeCl_3_, 4 μM CaCl_2_, 2 μM MnCl_2_, 2 μM ZnSO_4_, 400 nM CoCl_2_, 400 nM NiCl_2_, 400 nM CuCl_2_, 400 nM Na_2_MoO_4_, 400 nM Na_2_SeO_3_ and 400 nM H_3_BO_3_). The culture was grown at 37 °C with shaking at 200 rpm for 2 h, at which point the temperature was decreased to 22 °C and the culture was left to grow for 16 h.

Cells were harvested, lysed and purified following the protocol outlined earlier for MCL1 and MDM2, with some modifications. The cultures yielded a cell pellet amounting to 15 g L^−1^. Lysis was completed using an IKA T18 microfluidizer at 450 psi, followed by lysate clarification by centrifugation at 16,000*g* for 15 min. All IMAC steps were conducted at 22 °C, except for the incubation of the lysate–resin mixture, which was performed at 4 °C. Proteins bound to the resin were eluted with 5 ml of elution buffer (50 mM Tris-HCl pH 8, 250 mM NaCl and 300 mM imidazole). SEC was then performed using a Superdex 200 Increase 10/300 GL column (Cytiva) equilibrated with TBS (50 mM Tris-HCl pH 8 and 250 mM NaCl). Fractions confirmed by SDS–PAGE were pooled and concentrated using Amicon Ultra-15 concentrators with a 30 kDa cutoff (Millipore Sigma, UFC9030) to a final volume of 1 ml. Downstream processing for SPR analysis was performed as described previously, with one modification. For biotinylation, the protein was first cleaved using HRV 3C protease with the reagents and protocol provided by the Pierce HRV 3C protease solution kit (Thermo Scientific, 88946). The digested samples were subsequently purified and verified, as outlined in earlier sections.

#### GABARAP and GABARAPL1 for crystallography

GABARAP and GABARAPL1 were expressed as glutathione *S*-transferase (GST) fusion proteins after transforming *E.* *coli* BL21(DE3) T1 cells with pGEX4T2-GABARAP and pGEX4T2-GABARAPL1 plasmids, respectively. Bacteria were cultivated in LB medium containing 100 µg ml^−1^ ampicillin; gene expression was induced with 1 mM IPTG at an OD_600_ of 0.6–0.8 and allowed to proceed for 20 h at 25 °C. Afterward, cells were harvested by centrifugation at 3,000*g* for 30 min at 4 °C. The bacterial pellet was washed with PBS (137 mM NaCl, 2.7 mM KCl, 1.8 mM KH_2_PO_4_ and 10 mM Na_2_HPO_4_) and resuspended in lysis buffer (PBS supplemented with 5% (v/v) glycerol, 0.01% (v/v) β-mercaptoethanol, 10 µg ml^−1^ DNase (AppliChem, A3778) and cOmplete EDTA-free protease inhibitor cocktail (Roche, 11836170001)) before application to the cell disruptor (Constant Systems, model TS1.1) for three cycles with 1.9 kbar at 4 °C. Lysates were cleared by centrifugation at 4 °C with 45,000*g* for 45 min. The GST fusion proteins were purified from the supernatant by affinity chromatography using glutathione Sepharose 4B (Cytiva, 1705605). Cleavage with thrombin (Sigma-Aldrich, 1.12374) during dialysis against 10 mM Tris-HCl and 150 mM NaCl (pH 7.0) at 4 °C overnight yielded 119 amino acid proteins carrying an N-terminal Gly-Ser extension in addition to the native residues of GABARAP and GABARAPL1. Subsequently, samples were applied to a Hiload 26/60 Superdex 75 preparatory-grade size-exclusion column (GE Healthcare) equilibrated with 10 mM Tris-HCl and 150 mM NaCl (pH 7.0). Protein purity was assessed by SDS–PAGE and Coomassie staining. Fractions containing the eluted proteins were concentrated to 3–5 mg ml^−1^ using Vivaspin 20 concentrators with a 3 kDa cutoff (Sartorius), flash-frozen in liquid N_2_ and kept at −80 °C for long-term storage.

#### RbtA β-helix domain

For heterologous expression of the β-helix domain of RbtA (residues A20–I459) in *E.* *coli*, the gene was amplified and fused with a SNAC tag (GSHHWGS) at the C terminus using the following primers: forward, GCTGCCCAGCCGGCGATGGCCATGGGCGCTGATATTGAAGTCACAACTAC; reverse, CAGTGGTGGTGGTGGTGGTGCTCGAGGCTGCCCCAATGATGGCTGCCGATATATTCAATTGCGCCTAAAT^42^. The fragment was inserted into NcoI-digested and XhoI-digested pET-22b(+) by Gibson assembly to generate a construct with a C-terminal 6xHis fusion. The construct was confirmed by sequencing and transformed into *E.* *coli* Rosetta (DE3) cells.

To purify the β-helix domain of RbtA, an overnight culture of Rosetta (DE3) cells carrying the construct was back-diluted 1:300 in 2× YT broth and grown at 37 °C with shaking at 200 rpm until the OD_600_ reached 0.4. The incubation temperature was reduced to 18 °C, IPTG was added to a final concentration of 0.3 mM and the culture was incubated for a total of 18 h. Cells were then collected by centrifugation and resuspended in lysis buffer containing 200 mM NaCl, 50 mM Tris-HCl pH 7.5, 10% glycerol (v/v), 5 mM imidazole, 0.5 mg ml^−1^ lysozyme and 1 mU of benzonase. Cells were then lysed by sonication and cellular debris was removed by centrifugation at 35,000*g* for 30 min at 4 °C. The protein was purified from lysates using a 1 ml HisTrap HP column on an ÄKTA fast protein LC (FPLC) system. Column-bound protein was eluted using a linear imidazole gradient from 5 to 500 mM. Protein purity was assessed by SDS–PAGE and Coomassie staining. The fractions with high purity were concentrated using a 30 kDa cutoff Amicon filter and then further purified by FPLC using a HiLoad 16/600 Superdex 200 preparatory-grade column (GE Healthcare) equilibrated with sizing buffer (500 mM NaCl, 50 mM Tris-HCl pH 7.5 and 10% glycerol (v/v)). The fractions with high purity were concentrated and used for evaluation of macrocyclic binders or determination of X-ray structure.

For determination of the X-ray crystal structure of RbtA, the C-terminal 6xHis-tag was removed by chemical cleavage at the SNAC tag. In brief, the buffer of the concentrated protein was exchanged to cleavage buffer (0.1 M CHES, 0.1 M NaCl, 0.1 M acetone oxime and 5 mM Fos-choline-12, pH 8.6). The protein solution was diluted to 1 mg ml^−1^, followed by the addition of 1 mM TCEP and 1 mM NiCl_2_. The mixture was vortexed and incubated at room temperature for 16 h. The precipitation was removed by centrifugation at 35,000*g* for 30 min at 4 °C. The supernatant was concentrated and exchanged to Tris buffer (50 mM Tris-HCl pH 7.5 and 200 mM NaCl). The protein solution was incubated with a 1 ml bed volume of Ni-NTA beads to extract the cleaved 6xHis-tag. The resulting fraction was concentrated and then further purified by FPLC using a HiLoad 16/600 Superdex 200 preparatory-grade column.

### Crystallization of protein–cyclic peptide complexes

#### MCL1 with cyclic peptide

MCL1 (18.5 mg ml^−1^) and macrocycle MCB_D2 were mixed in 1:2 molar ratio and incubated for 30 min at room temperature. Upon addition of the MCB_D2 to the protein, we observed some precipitation. This precipitant was removed by centrifugation before crystallographic screening. Crystallization experiments for the MCL1–MCB_D2 complex were conducted using the sitting-drop vapor diffusion method. Initial crystallization trials were set up in 200 nl drops using 96-well crystallization plates. Crystal drops were imaged using the UVEX crystal plate hotel system by JANSi. Diffraction-quality crystals for the complex appeared in 0.2 M sodium chloride, 0.1 M Bis–Tris pH 6.5 and 25% (w/v) polyethylene glycol 3350 (Hampton Research) in 2 weeks.

#### GABARAP and GABARAPL1 with cyclic peptides

Cyclic peptides GAB_D8 and GAB_D23 were dissolved in 10 mM Tris-HCl and 150 mM NaCl (pH 7.0) and each mixed with both GABARAP and GABARAPL1, targeting a peptide-to-protein molar ratio of 3:2. After incubation for 10 min at room temperature, any insoluble components were removed by centrifugation (10 min at 20,000*g* and 4 °C). The protein–peptide complexes were concentrated using Amicon Ultra-0.5 centrifugal filter units with a 3 kDa cutoff (Merck) until a final protein concentration of 6–8 mg ml^−1^ (GABARAPL1–GAB_D8) or 13–15 mg ml^−1^ (GABARAP–GAB_D23) was reached. Samples were once again cleared of particles by centrifugation (30 min at 20,000*g* and 4 °C) before application in crystallization experiments. Search for crystallization conditions was performed by the sitting-drop vapor diffusion method using robotic systems Freedom Evo (Tecan) and Mosquito LCP (SPT Labtech) with commercially available screening sets. Experiments were set up by combining 200 nl of protein–peptide complex with 100 nl (for GABARAPL1–GAB_D8) or 200 nl (for GABARAP–GAB_D23) of reservoir solution and plates were incubated at 20 °C. Crystals appeared for a number of conditions, which were subjected to optimization as appropriate. Diffraction-quality samples used for X-ray structure determination developed with reservoir solutions containing 0.17 M ammonium sulfate, 25.5% (w/v) PEG 4000 and 15% (v/v) glycerol for GABARAPL1–GAB_D8 and 0.1 M MES pH 5.0 and 30% (w/v) PEG 6000 in the case of GABARAP–GAB_D23. Diffraction data (https://doi.esrf.fr/10.15151/ESRF-DC-1966164200 and https://doi.esrf.fr/10.15151/ESRF-DC-1979522808 for GABARAPL1–GAB_D8 and GABARAP–GAB_D23, respectively) were collected at 100 K on beamline BM07 of the European Synchrotron Radiation Facility (ESRF) tuned to an X-ray wavelength of 0.9795 Å, using a Pilatus 6M detector (DECTRIS). Data processing was carried out with XDS and XSCALE^43^ and included reflections up to a diffraction limit of 1.5 Å for GABARAP–GAB_D23 and 2.5 Å for GABARAPL1–GAB_D8. The GABARAP–GAB_D23 structure featuring space group C2 was determined by molecular replacement (MR) using MOLREP^44^ with the structure of GABARAP from its K1 peptide complex (PDB 3D32)^30^ as a template. For the GABARAPL1–GAB_D8 complex, initial evaluation suggested tetragonal symmetry but with strong indications of twinning. Data integration in maximal translationengleiche subgroups followed by MR search using MoRDa^45^ revealed P2_1_2_1_2_1_ as the true space group, with near-perfect pseudomerohedral twinning accounting for apparent Laue group 4/mmm. To avoid bias in cross-validation, this pseudosymmetry of the data was explicitly accounted for in flag assignment. The solution obtained for GABARAPL1–GAB_D8 was subjected to a round of automated rebuilding in phenix.autobuild^46^. In either case, model refinement was performed with phenix.refine^47^, alternating with interactive rebuilding in Coot^48^, which included stepwise introduction of cyclic peptides GAB_D8 and GAB_D23. According to validation using MolProbity^49^ and the wwPDB validation system (https://validate-rcsb-2.wwpdb.org/), both models featured good geometry. Detailed statistics of data collection and refinement can be found in Supplementary Table 6.

#### RbtA with cyclic peptide and apo RbtA

RbtA (10 mg ml^−1^) and RBB_D10 were mixed in a 1:5 molar ratio and incubated for 30 min at room temperature. Initial crystallization trials were set up in 200 nl drops using 96-well crystallization plates and the experiments were conducted by the sitting-drop vapor diffusion method. Crystal drops were imaged using the UVEX crystal plate hotel system by JANSi. Diffraction-quality crystals for the RbtA–RBB_D10 complex appeared in 0.2 M lithium sulfate, 0.1 M Tris pH 8.5 and 40% (v/v) PEG 400 (JCSG Plus, Hampton Research). Additionally, we soaked the crystals in 22.32 mg ml^−1^ RBB_D10 for 5 min before flash-freezing. Crystals for RbtA alone (18.7 mg ml^−1^) were grown in 0.1 M Bis–Tris pH 6.5 and 20% (v/v) PEG 5,000 MME (SG1, Molecular Dimensions). All crystals were flash-cooled in liquid nitrogen before shipping to the synchrotron for data collection.

Diffraction data were collected at the NSLS2 beamline AMX/FMX (17-ID-1/17-ID-2). X-ray intensities and data reduction were evaluated and integrated by XDS^43^ and merged and scaled by Pointless and Aimless in the CCP4i2 program suite^50^. The X-ray crystal structure was determined by MR using the designed model for phasing by Phaser^51^. Next, the structure obtained from the MR was improved and refined by Phenix^47^. Model building was performed by Coot^48^ in between the refinement cycles. The final model was evaluated by MolProbity^49^. Data collection and refinement statistics are reported in Supplementary Table 5.

### SPR

SPR experiments were performed using a Cytiva Biacore 8K in HBS-EP+ buffer from Cytiva. Measurements were obtained by immobilization of biotinylated target protein using the biotin capture kit from Cytiva. Binding screens were performed by single-cycle kinetics experiments using the standard protocol in the Biacore 8K control software at 30 µl min^−1^ with serial injections of 10 nM, 100 nM, 1 µM, 10 µM and 100 µM, an association time of 60 s and a dissociation time of 120 s. For MCL1 designs, a dissociation time of 150 s was used. To evaluate the affinity of successful designs, a nine-point single-cycle kinetics experiment was performed with an association time of 90 s and dissociation time of 300 s. The dilution series for MCB_D2 was twofold starting at 20 µM, that for MDB_D8 was fivefold starting at 50 µM, and those for GAB_D8, GAB_D23 and RBB_D10 were fivefold starting at 20 µM. Reported measurements were analyzed using Biacore Insight evaluation software; sensorgrams were double-referenced and fit with a 1:1 binding kinetics fit model.

### AlphaScreen assay

We used the AlphaScreen assay as described by Leveille et al.^52^ to measure inhibition of the GABARAP–K1 interaction by the computationally designed macrocycles. K1 is a previously described GABARAP binder with a *K*_d_ of 10 nM (ref. ^27^). Biotin-labeled peptide K1 was used at a final concentration of 10 nM and incubated with 10 nM (final concentration) of 6xHis–GABARAP in a final reaction volume of 50 μl. Computationally designed inhibitor peptides were serially diluted with 1:3 dilutions using the highest final concentration of 50 μM and added to the reaction mixture. The buffer used was 25 mM HEPES pH 7.3, 150 mM NaCl, 0.01% Tween, 1 mg ml^−1^ BSA and 0.5% DMSO. The plate was covered in foil, centrifuged at 1,500 rpm for 2 min and incubated for 150 min at room temperature with shaking. Then, 20 μg ml^−1^ (final concentration) of the streptavidin donor beads and nickel chelate acceptor beads were added in the dark before incubating for another 45 min. Data were collected on a Tecan plate reader using excitation at 680 nm and emission at 520–620 nm. Data were normalized to 0% (buffer only) and 100% (protein and tracer peptide, no inhibitor) controls. IC_50_ values were obtained from curve fits using GraphPad Prism 9 software, using the equation $Y=\frac{100}{(1+{(\frac{X}{{{IC}}_{50}})}^{h})}$, where *X* is the concentration of inhibitor and *h* is the Hill coefficient. At least three independent replicates were used to calculate the average IC_50_ and the s.e.m.

### Statistics and reproducibility

No statistical method was used to predetermine sample size. One trial from the AlphaScreen that was used to determine the IC_50_ of GAB_D8 was repeated and the repeated value is what was used. All data are included in the Source Data. The experiments were not randomized. The investigators were not blinded to allocation during experiments and outcome assessment.

### Reporting summary

Further information on research design is available in the Nature Portfolio Reporting Summary linked to this article.

## Online content

Any methods, additional references, Nature Portfolio reporting summaries, source data, extended data, supplementary information, acknowledgements, peer review information; details of author contributions and competing interests; and statements of data and code availability are available at 10.1038/s41589-025-01929-w.

## Supplementary information

Supplementary Information Supplementary Sections 1–5, Figs. 1–59 and Tables 1–10.

Reporting Summary

## Source data

Source Data Fig. 1 Source data used in plots.

Source Data Fig. 2 Source data used in plots.

Source Data Fig. 3 Source data used in plots.

Source Data Fig. 4 Source data used in plots.

## Data Availability

The design models and sequences are available in Supplementary Information. Crystal structures of MCB_D2 bound to MCL1, GAB_D8 bound to GABARAPL1, GAB_D23 bound to GABARAP, RBB_D10 bound to RbtA and apo RbtA were deposited to the PDB under accession codes 9CDT, 9HGC, 9HGD, 9CDU and 9CDV, respectively. Source data are provided with this paper.

## Code availability

The code and scripts for running RFpeptides are available from Zenodo (10.5281/zenodo.15264344)^53^. The code and scripts for RFpeptides are also available from RFdiffusion GitHub repository (https://github.com/RosettaCommons/RFdiffusion).
