# Supplementary material for: Accurate de novo design of high-affinity protein-binding macrocycles using deep learning
Source: Nat Chem Biol. 2025 Jun 20;21(12):1948–56. doi: 10.1038/s41589-025-01929-w (PMC12643943; doi:10.1038/s41589-025-01929-w)
Supplement: Supplementary file 1 — Supplementary Sections 1–5, Figs. 1–59 and Tables 1–10. [file 41589_2025_1929_MOESM1_ESM.pdf]

nature chemical biology

Supplementary information https://doi.org/10.1038/s41589-025-01929-w

Accurate de novo design of high-affinity protein-binding macrocycles using deep learning

In the format provided by the authors and unedited

SUPPLEMENTARY INFORMATION

Accurate *de novo* design of high–affinity protein binding macrocycles using deep learning

Stephen A. Rettie[*,1,2,3], David Juergens[*,2,4], Victor Adebomi[*,1,2], Yensi Flores Bueso[1,2,5,6], Qinqin Zhao[7], Alexandria N. Leveille[8], Andi Liu[7], Asim K. Bera[2], Joana A. Wilms[9,10], Alina Üffing[9,10,14], Alex Kang[2], Evans Brackenbrough[2], Mila Lamb[2], Stacey R. Gerben[2], Analisa Murray[2], Paul M. Levine[2], Maika Schneider[1,2,11], Vibha Vasireddy[1,2], Sergey Ovchinnikov[12], Oliver H. Weiergräber[10], Dieter Willbold[9,10], Joshua A. Kritzer[8], Joseph D. Mougous[7,13], David Baker[#,2,5,13], Frank DiMaio[#,2,5], Gaurav Bhardwaj[#,1,2]

Author Affiliations:

1. Department of Medicinal Chemistry, University of Washington, Seattle, WA, USA
2. Institute for Protein Design, University of Washington, Seattle, WA, USA
3. Molecular and Cellular Biology Program, University of Washington, Seattle, WA, USA
4. Graduate Program in Molecular Engineering, University of Washington, Seattle, WA, USA
5. Department of Biochemistry, University of Washington, Seattle, WA, USA
6. Cancer Research @UCC, University College Cork, Cork, Ireland
7. Department of Microbiology, University of Washington, Seattle, WA, USA
8. Department of Chemistry, Tufts University, 62 Talbot Avenue, Medford, MA, USA
9. Heinrich–Heine–Universität Düsseldorf, Institut für Physikalische Biologie, Düsseldorf, Germany
10. Forschungszentrum Jülich, Institute of Biological Information Processing, Structural Biochemistry (IBI–7), Jülich, Germany
11. Department of Chemistry, University of Washington, Seattle, WA, USA
12. Department of Biology, Massachusetts Institute of Technology, Cambridge, MA, USA
13. Howard Hughes Medical Institute, University of Washington, Seattle, WA, USA
14. Current address: Molecular Cell Biology of Autophagy Laboratory, The Francis Crick Institute, 1 Midland Road, London NW1 1AT, UK

*These authors contributed equally. SAR, DJ, and VA agree that the order of their respective names on the author list may be changed for their professional interests.

#Corresponding authors: Gaurav Bhardwaj (gauravb@uw.edu), Frank DiMaio (dimaio@uw.edu), and David Baker (dabaker@uw.edu)

Table of Contents

1. Supplementary Figures 1 to 20 and Supplementary Tables 1 to 4
2. Materials and Methods
    - 2.1. Macrocycle monomer design with RFpeptides
        - 2.1.1. Implementation of the cyclic relative position encodings for RoseTTAFold and RFdiffusion
        - 2.1.2. Monomeric macrocycle prediction with RFpeptides
        - 2.1.3. Monomeric macrocycle design with RFpeptides
        - 2.1.4. Visualization and structural clustering of monomeric designs
    - 2.2. Macrocycle Binder Design with RFpeptides
        - 2.2.1. Macrocycle Backbone Generation
        - 2.2.2. Sequence Design
    - 2.3. Target–macrocycle complex structure prediction using AfCycDesign
    - 2.4. Filtering designs based on physics–based metrics of interface quality
    - 2.5. Structure–based clustering for binder design campaigns
    - 2.6. Prediction of RbtA Structure
3. Data Collection and Refinement Statistics
    - 3.1. Supplementary Tables 5 and 6
4. Peptide Analytical Characterization Data
    - 4.1. Supplementary Tables 7-10
    - 4.2. Supplementary Figures 21-59
5. Supplementary References

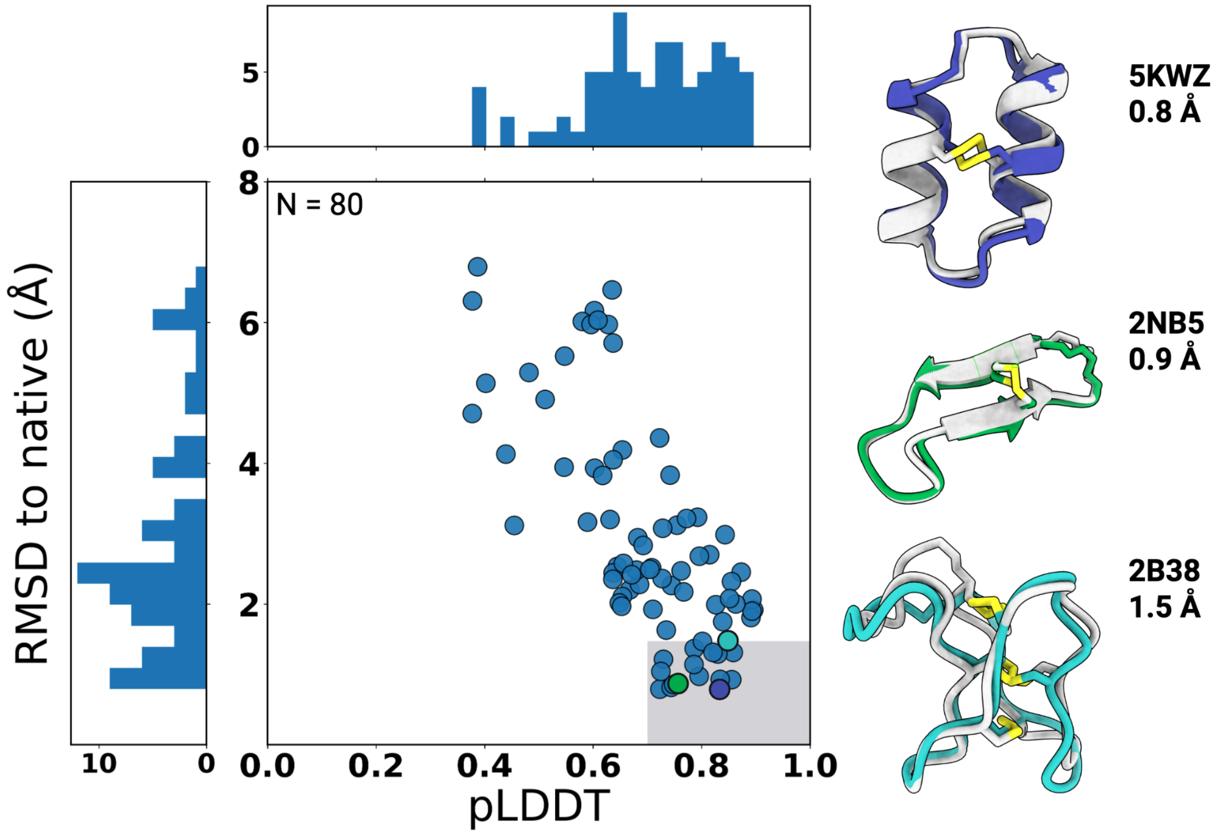

Supplementary Figure 1: Structure prediction of cyclic peptides using RoseTTAFold2 (RF2) modified with cyclic positional encoding.
Structures for 80 cyclic peptides from the Protein Data Bank were predicted using RF2 modified with cyclic positional encoding. Each PDB entry is an NMR structure. The lowest pLDDT prediction was compared to all members of the NMR ensemble and the lowest backbone RMSD is plotted. Three selected examples from the benchmark set are shown with the NMR structure in gray and the predicted structure colored corresponding to its position in the plot. The boxed gray area encloses 17 high–confidence and accurate designs that were predicted within 1.5 Å of the NMR structure and with pLDDT of 0.7 or above.

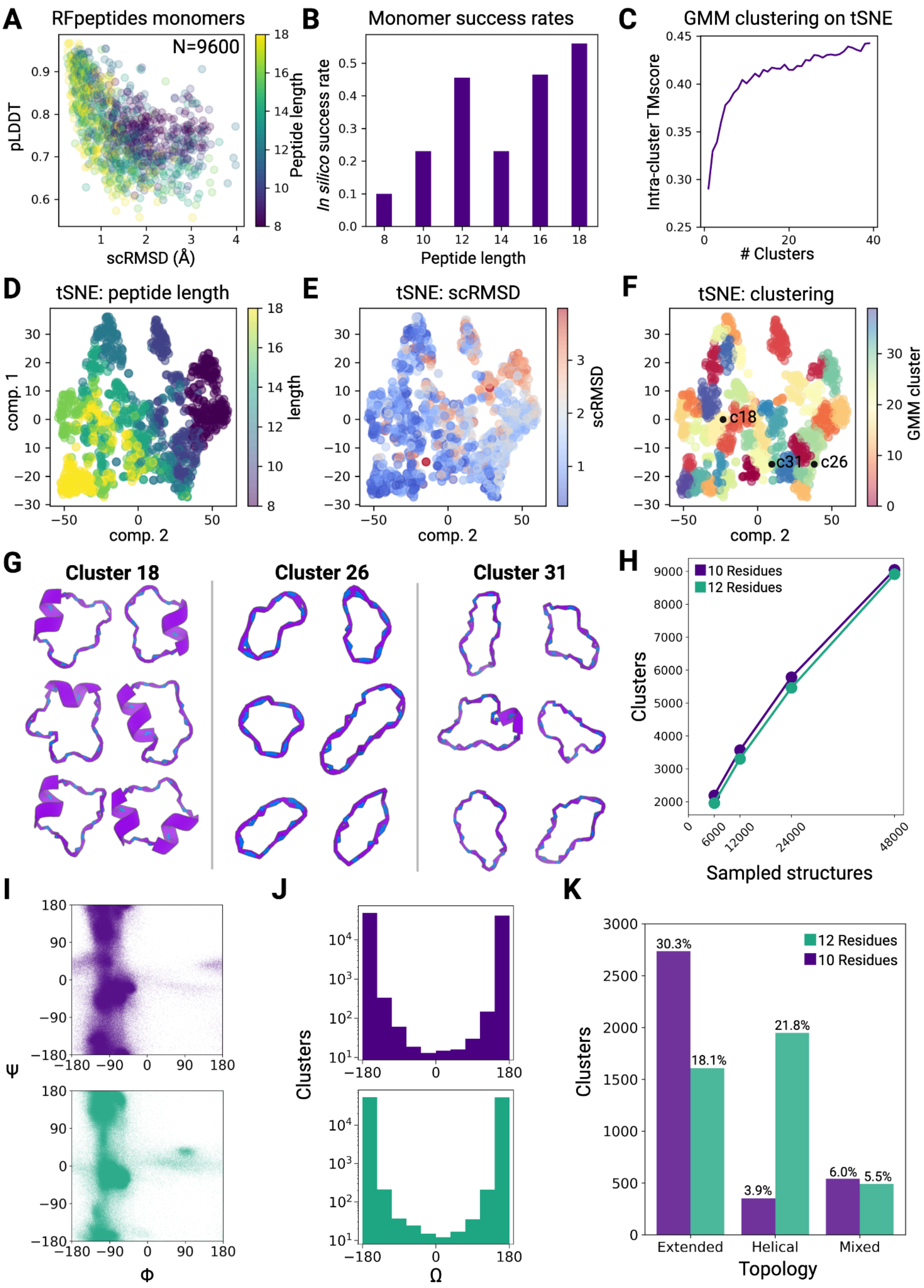

A
RFpeptides monomers
N=9600
pLDDT
scRMSD (Å)
Peptide length
B
Monomer success rates
In silico success rate
Peptide length
C
GMM clustering on tSNE
Intra-cluster TMscore
Clusters
D
tSNE: peptide length
comp. 1
comp. 2
length
E
tSNE: scRMSD
scRMSD
F
tSNE: clustering
GMM cluster
c18
c31
c26
G
Cluster 18
Cluster 26
Cluster 31
H
10 Residues
12 Residues
Clusters
Sampled structures
I
Ψ
Φ
J
Clusters
Ω
K
12 Residues
10 Residues
30.3%
18.1%
3.9%
21.8%
6.0%
5.5%
Extended
Helical
Mixed
Topology

Supplementary Figure 2: *In silico* analysis of monomeric cyclic peptide generation by RFpeptides. For each length in [8,10,12,14,16,18] amino acids, 200 cyclic peptide backbones were generated with RFpeptides. 8 amino acid sequences per backbone were then designed with LigandMPNN, and their structures predicted with AfCycDesign. (A) scRMSD ("self–consistency" RMSD) was calculated as the RMSD between the RFdiffusion output and AfCycDesign prediction (best of 8 LigandMNN sequences), and plotted against AfCycDesign pLDDT. (B) *In silico* success rates for peptides of varying length generated by the RFpeptides pipeline. A design is successful if its scRMSD < 2.0 Å and pLDDT > 0.8. This panel is reproduced with data from Figure 1 panel C for ease of reference and clarity. (C) Plot of intra–cluster mean TMscore versus number of clusters used when performing Gaussian mixture model (GMM) cluster assignment on 2D tSNE (see Methods 1.1.4 for more details). The TMscore metric reported here is the average of the query– and target–normalized TMscores output from TMalign. (D,E,F) tSNE components 1 and 2 plotted, and colored by (D) peptide length, (E) scRMSD and (F) cluster assignment after GMM clustering (reproduced from Fig 1B for ease of comparison). (G) Representative examples of generated backbones with < 2.0 Å scRMSD, cluster 18, cluster 26 and cluster 31. We note two observations on the panels (G): First, it can be seen that internally to a given cluster, the structures are qualitatively more similar to each other than to structures in other clusters. E.g., Samples from cluster 26 are mostly extended, occasionally strand-pairing peptides with no alpha–helical structure, while samples from cluster 18 and 0 have little beta character and often contain helices. Second, although intra–cluster similarity is higher than inter–cluster similarity, there is still a wide range of significantly differing structures within each cluster. We attribute the "coarseness" of these clusters to the low overall number of Gaussians used (40 total) in the Gaussian mixture model. As can be seen in Fig. S2 C, using more Gaussians in the GMM results in higher intra–cluster TMscore, suggesting that if more clusters were used (e.g., hundreds), the clusters would become increasingly structurally uniform. (H) To assess the diversity of the space that can be sampled by RFpeptides we performed a larger scale backbone generation experiment of 48,000 for two sizes, 10 and 12 residue peptides. We clustered these designs by Cα RMSD of 0.5 Å as previously described[1]. 10mer and 12mer backbone generation produced 9,045 and 8,913 unique backbones when clustered by this method, This suggests that there is significant diversity to be explored. (I) Phi (Φ) and psi (Ψ) angle distribution for all 48,000 structures. (J) Omega (Ω) angle distribution for cluster centers for 10 and 12 residue peptides. (K) Topological distribution of cluster centers as determined by DSSP[2]. To correct for cyclization, all cyclic permutations were calculated and combined to generate the DSSP string. Extended and helical

are defined as having at least one E or H respectively and none of the other in the combined string, mixed contain at least one of each.

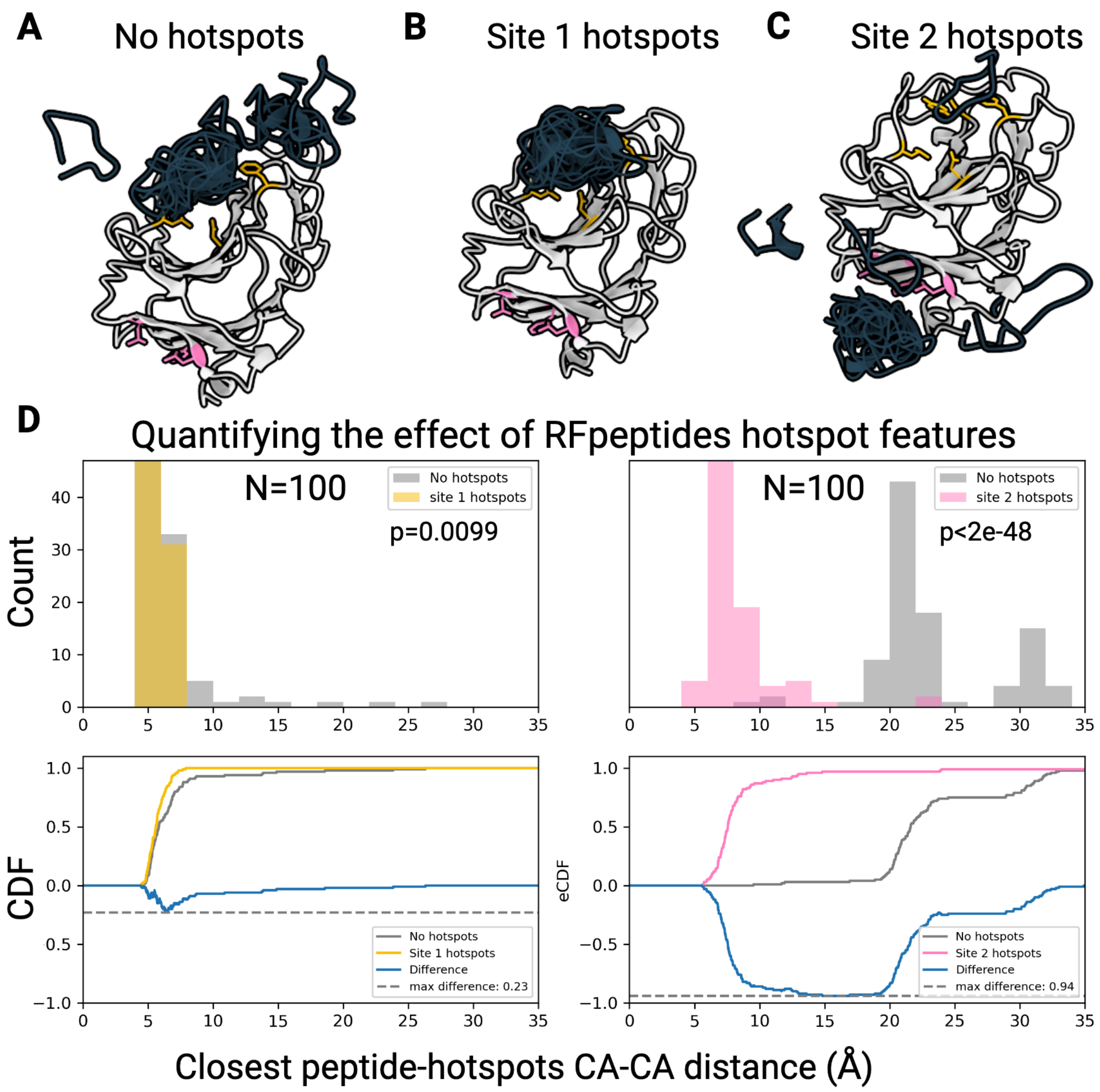

Supplementary Figure 3: Impact of specifying hotspot residues for cyclic peptide binder design

RFpeptides macrocyclic peptide diffusion was sampled N=100 times using (A) no hotspot features, (B) hotspot features targeting residues Asp77, Tyr50, Phe171, Leu93 (“site 1” in yellow), (C) hotspot features targeting residues Phe131, Val138, Leu150 (“site 2” in pink). (D) For each of sites 1–3, with and without hotspot features provided, we computed the distribution of minimum Cα–Cα distances between any residue in the generated peptide and either of the two residues in the site (top). A two–sided Kolmogorov–Smirnov test was performed for each site, testing the hypothesis that the addition of hotspot features changes the distribution of minimum distances between the generated peptides and the epitope to be targeted. The null hypothesis (“hotspot features have no effect on the minimum distance distribution”) is rejected with p–value 0.0099 for site 1, and $p < 2e^{-48}$ for site 2.

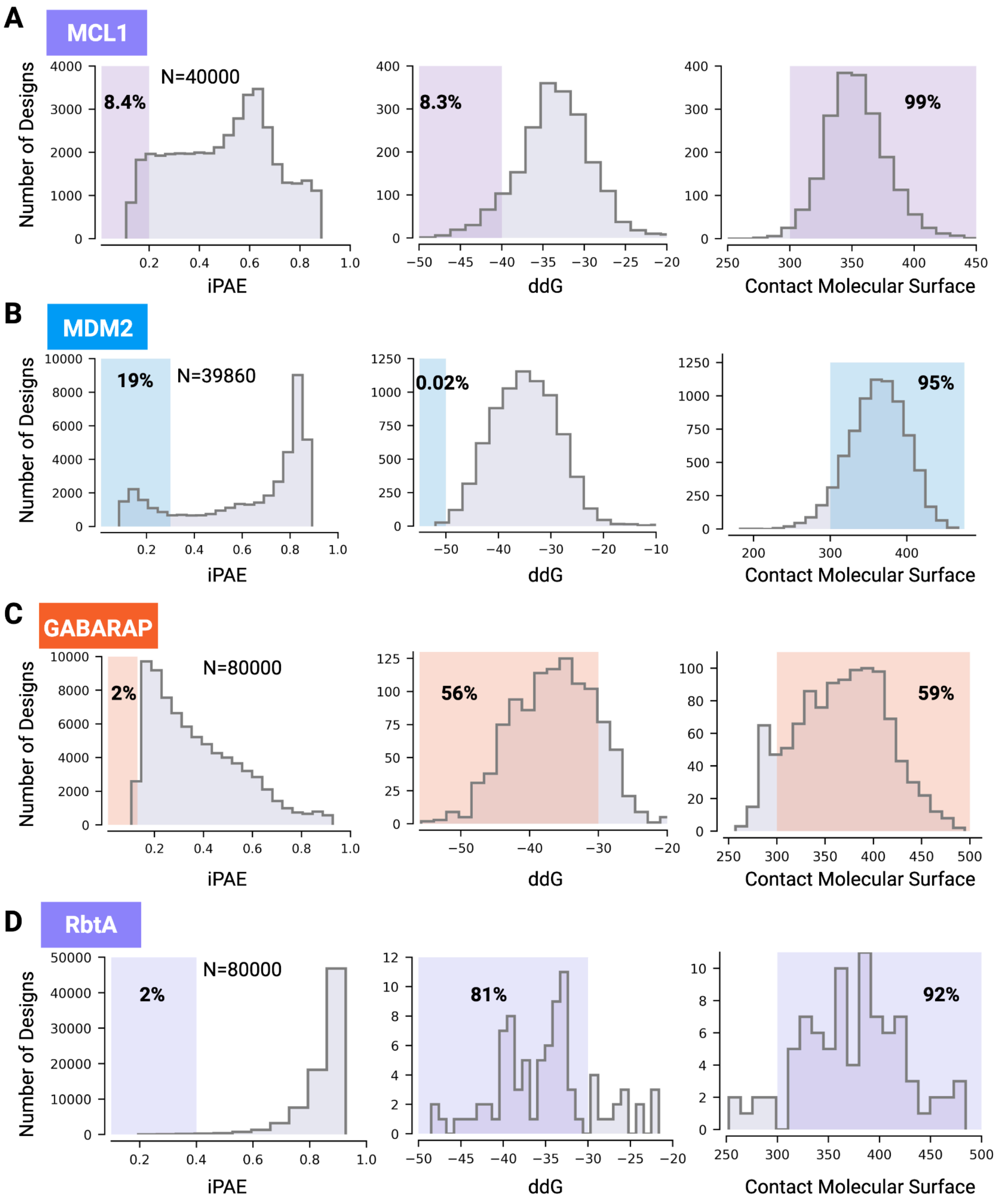

Supplementary Figure 4: Distribution of *in silico* scores used in downselection of design models

Distribution of the normalized interface PAE (iPAE) score from AfCycDesign predictions (left), Rosetta ddG (kcal/mol) (middle) and the contact molecular surface area (Å^2) (right) for the computationally generated macrocyclic binders against (A) MCL1, (B) MDM2, (C) GABARAP,

and (D) RbtA. For all four targets, designs were first filtered by normalized iPAE from the AfCycDesign prediction of the macrocycle–bound target complex, followed by further filtering using Rosetta ddG and the contact molecular surface area. The percentage of total designs passing the cutoff values (highlighted region) denoted in each plot.

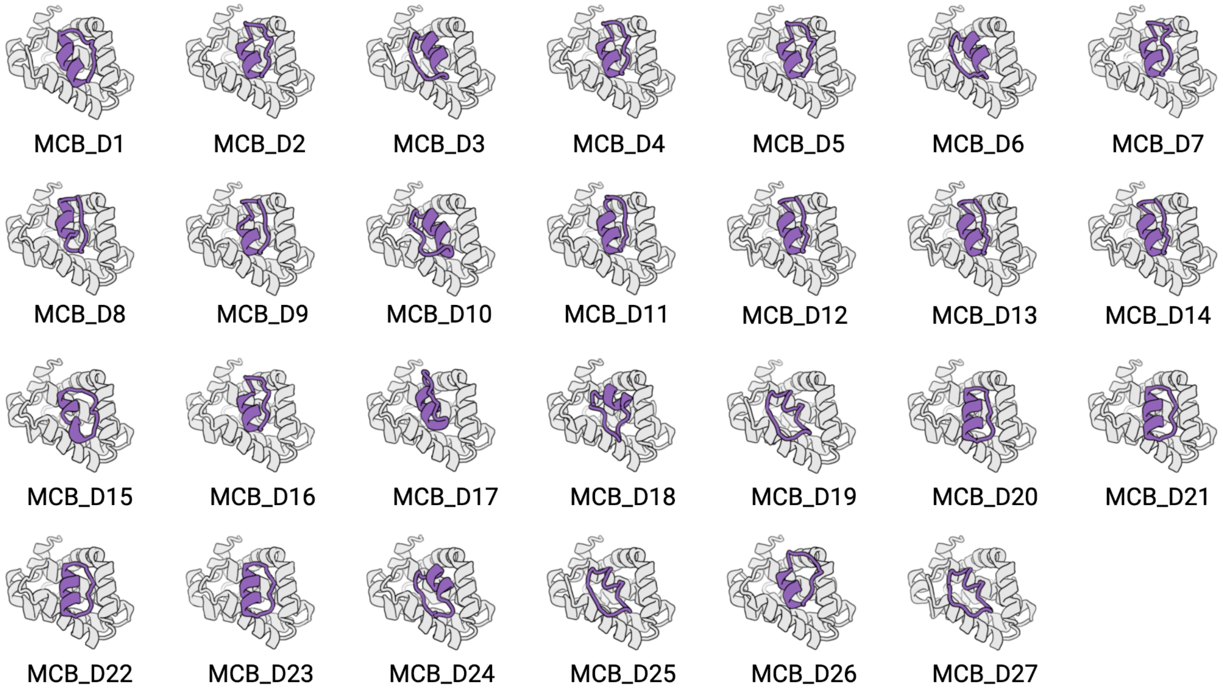

Supplementary Figure 5: Structural diversity of the selected MCL1 binding designs
Computational models for 27 putative MCL1–binding macrocycles selected after filtering using AfCycDesign (normalized iPAE < 0.2), RoseTTAFold (iPAE < 6 and Cα RMSD to the design model less than 1 Å) and Rosetta interface metrics (ddG < –40 kcal/mol, SAP < 35, CMS > 300 Å^2). Two rank–ordered lists of candidates were generated, one using Rosetta metrics and the other using iPAE < 0.13. The two lists were merged, leaving 27 designs spanning those with the best Rosetta metrics, those with the best AfCycDesign metrics, and those with both.

Supplementary Table 1a: Sequences and interacting residues for selected MCL1 designs

Design	Sequence	Length	Interacting Residues (< 4 Å)	Net Charge (pH 7.4)*	Hydrophobicity %**
MCB_D1	GAPEILKKMADMVGYE	16	H224,T226,A227,F228,G230,M231,R233,K234,L235,V249,M250,H252,V253,F254,D256,G257,N260,G262,R263,T266,L267,F270	-1.45	0.500
MCB_D2	PPEIAWLADAVGLKDA	16	V220,H224,A227,F228,G230,M231,K234,L235,V249,H252,V253,D256,R263,T266,L267,F270	-2.1	0.625
MCB_D3	GLETDDPVVKPLADAV	16	H224,A227,F228,M231,K234,L235,V249,H252,V253,D256,G257,R263,T266,L267,F270	-3.44	0.563
MCB_D4	APPEIKALADAVGLED	16	V220,H224,A227,F228,G230,M231,K234,L235,V249,H252,V253,R263,T266,L267,F270	-3.39	0.625
MCB_D5	APESIKWLADAVGLKD	16	H224,A227,F228,G230,M231,K234,L235,V249,H252,V253,D256,R263,T266,L267,F270	-1.39	0.500
MCB_D6	SLIKPLADAIGVETDD	16	H224,A227,F228,M231,K234,L235,V249,H252,V253,D256,R263,T266,L267,F270	-3.75	0.500
MCB_D7	SSTVAFLQEAVGMPVT	16	V220,H224,A227,F228,G230,M231,K234,L235,V249,H252,V253,D256,R263,T266,L267,F270	-1.75	0.563
MCB_D8	EAVGLESDSELIKKLA	16	V220,H224,A227,F228,G230,M231,K234,L235,V249,H252,V253,D256,G262,R263,V265,T266,L267,F270	-2.33	0.438
MCB_D9	KPLAEAVGVETDIPTV	16	V220,H224,A227,F228,G230,M231,K234,L235,V249,H252,V253,R263,T266,L267,F270	-2.44	0.563
MCB_D10	PAESELIAPLAEAVGI	16	H224,A227,F228,M231,K234,L235,V249,M250,H252,V253,S255,D256,R263,T266,L267,F270	-3.1	0.688
MCB_D11	LEVPDTIVKPLAEAIG	16	V220,H224,A227,F228,G230,M231,K234,L235,V249,H252,V253,G262,R263,V265,T266,L267,F270	-2.44	0.625
MCB_D12	SVDDPLIRKLAEAVGL	16	V220,H224,A227,F228,G230,M231,K234,L235,V249,H252,V253,D256,G262,R263,V265,T266,L267,F270	-1.75	0.563
MCB_D13	SIDDPLLKKLAEAVGL	16	V220,H224,A227,F228,G230,M231,K234,L235,V249,H252,V253,D256,G262,R263,T266,L267,F270	-1.75	0.563

MCB_D14	SIDDPLLRPLAEAVGL	16	H224,A227,F228,G230,M231,K234,L235,V249,H252,V253,G262,R263,T266,L267,F270	-2.74	0.625
MCB_D15	ADALKRLTVPKNLEEI	16	V220,H224,T226,A227,F228,G230,M231,K234,L235,V249,H252,V253,S255,D256,R263,T266,L267,F270	-0.39	0.500
MCB_D16	PPEVAFLADAVGLKDA	16	V220,H224,A227,F228,G230,M231,K234,L235,V249,H252,V253,R263,T266,L267,F270	-2.1	0.688
MCB_D17	ERDDIIGPLVKAILGE	16	V220,H224,A227,F228,G230,M231,K234,L235,V249,H252,V253,G262,R263,V265,T266,L267,F270,F319	-2.33	0.500
MCB_D18	KPQPDIDPAFAEIVGF	16	H224,A227,F228,G230,M231,K234,V249,H252,V253,S255,D256,R263,T266,L267,F270	-2.44	0.625
MCB_D19	LAKIVGVETDDPVFAD	16	H224,A227,F228,M231,K234,L235,L246,V249,M250,H252,V253,F254,D256,G257,R263,T266,L267,F270	-3.44	0.563
MCB_D20	EDTLEGIARGLLTGKV	16	A227,F228,G230,M231,K234,L235,S245,R248,V249,H252,V253,R263,T266,L267,F270	-1.33	0.375
MCB_D21	YDTEEGIAEGLLTGKV	16	H224,T226,A227,F228,G230,M231,K234,L235,S245,L246,R248,V249,H252,V253,R263,T266,L267,F270	-3.44	0.313
MCB_D22	ADTEEGIAKGLLTGKV	16	H224,T226,A227,F228,G230,M231,K234,L235,R248,V249,H252,V253,R263,T266,L267,F270	-1.39	0.375
MCB_D23	ADTIEGIAKGLLTGEV	16	H224,T226,A227,F228,G230,M231,K234,L235,S245,R248,V249,H252,V253,R263,T266,L267,F270	-2.39	0.438
MCB_D24	PEIAEDSRQIFGVDLD	16	H224,A227,F228,G230,M231,K234,L235,V249,M250,H252,V253,S255,D256,R263,T266,L267,F270	-4.09	0.438
MCB_D25	LAKIVGVETSDEKIED	16	H224,A227,F228,M231,K234,L235,L246,V249,M250,H252,V253,F254,D256,G257,R263,T266,L267,F270	-3.44	0.375
MCB_D26	GSPEIRWLMDAFGVDE	16	N223,H224,T226,A227,F228,G230,M231,R233,K234,L235,L246,V249,H252,V253,D256,R263,T266,L267,F270	-3.44	0.438
MCB_D27	LAKIVGVTDAPEEIAD	16	H224,A227,F228,M231,K234,L235,L246,V249,M250,H252,V253,F254,D256,G257,R263,T266,L267,F270	-3.44	0.563

*Net charge calculated using BioPython(Cock et al. 2009)
Bio.SeqUtils.ProtParam.charge_at_pH
**Percent of sequence that is A,V,I,L,M,F,P

Supplementary Table 1b: Metrics for selected MCL1 designs

Design	Contact Molecular Surface (Å^2)	ddG (kcal/mol)	SAP	iPAE
MCB_D1	347.474	-33.596	26.329	0.1298
MCB_D2	356.288	-42.945	28.76	0.1266
MCB_D3	344.933	-29.103	23.803	0.1141
MCB_D4	344.244	-38.251	23.976	0.1263
MCB_D5	362.657	-42.337	27.32	0.1157
MCB_D6	346.064	-31.909	23.8	0.1218
MCB_D7	364.17	-42.112	25.131	0.1221
MCB_D8	374.344	-35.356	24.323	0.1177
MCB_D9	334.056	-38.602	24.923	0.1204
MCB_D10	371.381	-31.084	32.415	0.1223
MCB_D11	371.957	-35.851	33.377	0.1184
MCB_D12	368.255	-35.467	25.654	0.1177
MCB_D13	348.625	-38.32	27.171	0.1213
MCB_D14	340.727	-36.487	29.204	0.1231
MCB_D15	394.557	-36.539	28.061	0.1290
MCB_D16	362.535	-42.683	29.823	0.1307
MCB_D17	394.018	-41.83	25.966	0.1432
MCB_D18	341.545	-40.808	27.107	0.1967
MCB_D19	395.401	-42.277	27.369	0.1913
MCB_D20	356.261	-41.452	24.586	0.1711
MCB_D21	382.798	-44.511	19.423	0.1540
MCB_D22	371.888	-42.217	19.119	0.1605
MCB_D23	387.222	-44.202	24.754	0.1791
MCB_D24	355.614	-40.979	22.236	0.1647
MCB_D25	399.575	-40.165	20.273	0.1602
MCB_D26	391.315	-44.576	25.259	0.1927
MCB_D27	390.125	-40.087	24.266	0.1699

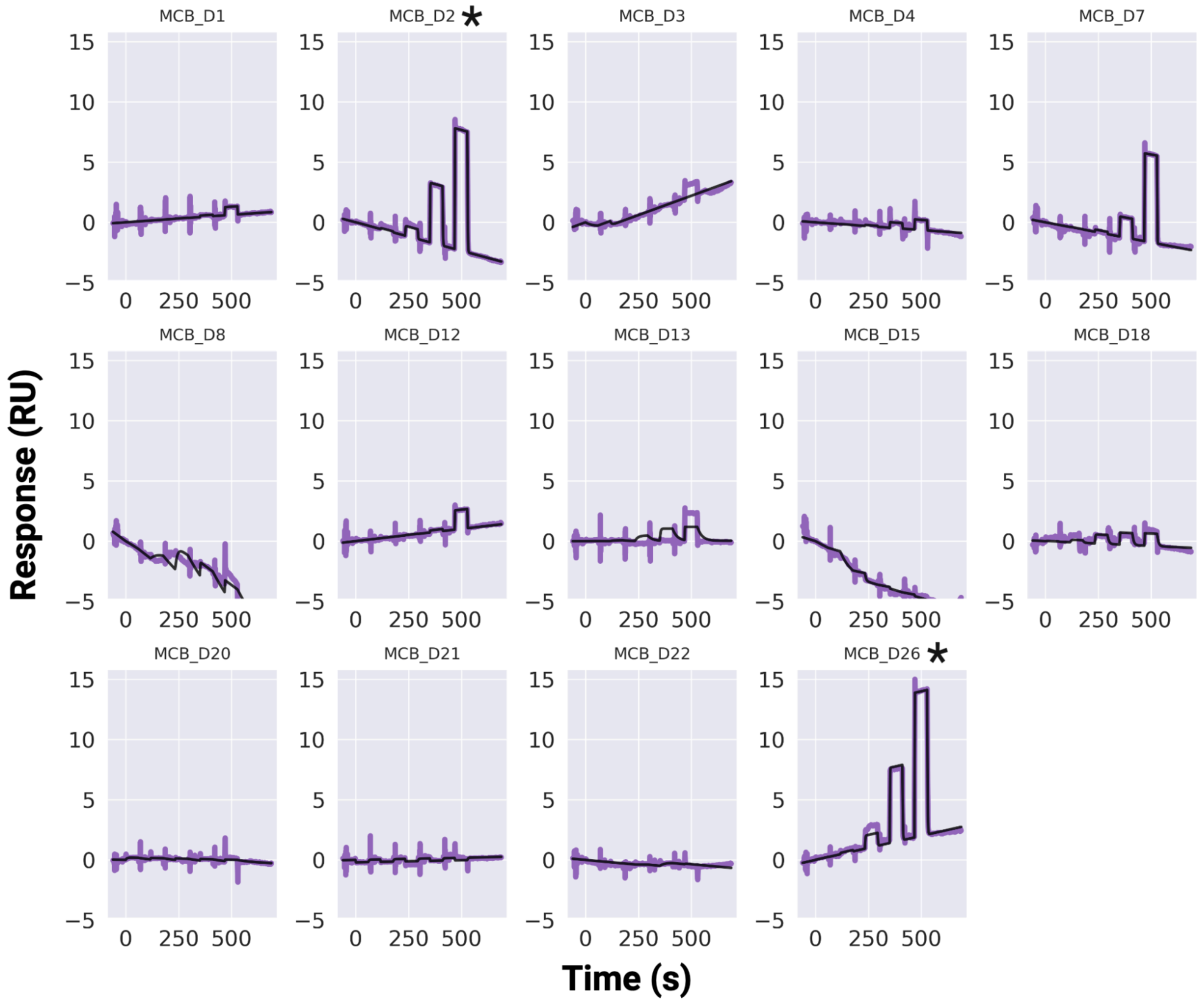

Supplementary Figure 6: SPR–based binding screen of the selected MCB designs against MCL1

Single–cycle kinetics SPR binding screen for selected MCB designs, applying a 5–point 10–fold dilution with 100 μM as the highest concentration. Three designs (MCB_D2, MCB_D7, and MCB_D26) demonstrate detectable binding signals at or below 100 μM. MCB_D2 and MCB_D26 denoted with * were chosen for further characterization.

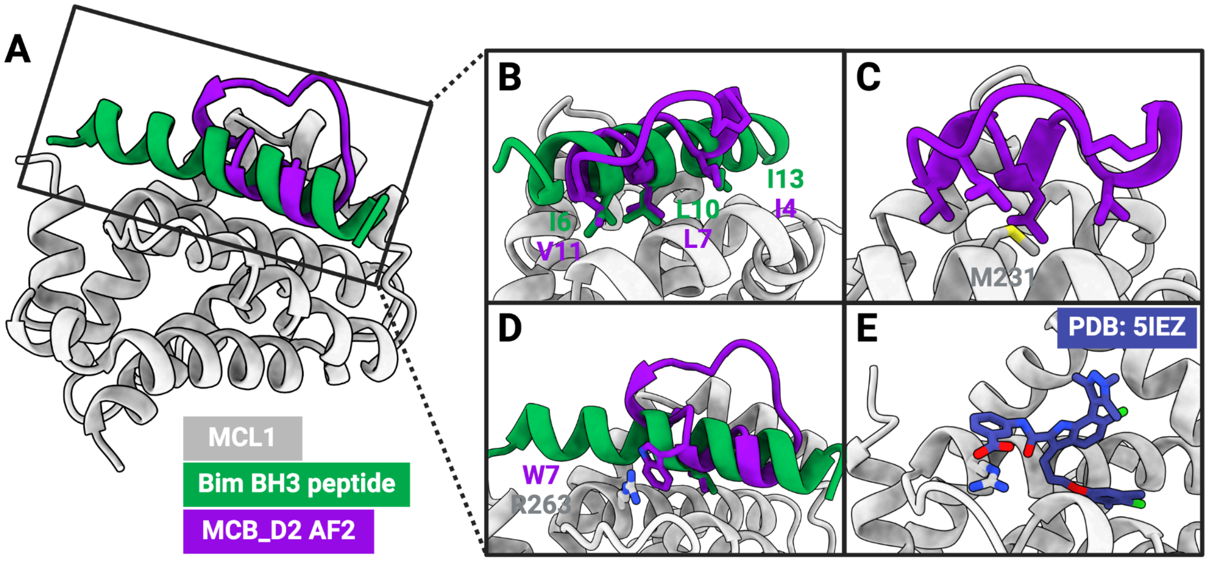

Supplementary Figure 7: Comparison between the MCL1–MCB_D2 interactions and interactions made by previously described MCL1 binders

(A) Alignment of Bim–BH3 (PDB: 2PQK[3]) to the design model for MCB_D2 demonstrates that the direction of the interacting helix is opposite across the two cases. (B) Some of the side chain placements at the interface of the Bim–BH3–MCL1 complex are mimicked in the design model of MCB_D2. (C) The helix and loop regions of MCB_D2 make hydrophobic contacts with MCL1 (D) Cation–π interaction between W7 of MCB_D2 and R263 of MCL1 not present in native Bim–BH3. (E) A small–molecule ligand bound to MCL1 (PDB: 5IEZ[4]) shows a similar cation–π interaction with R263.

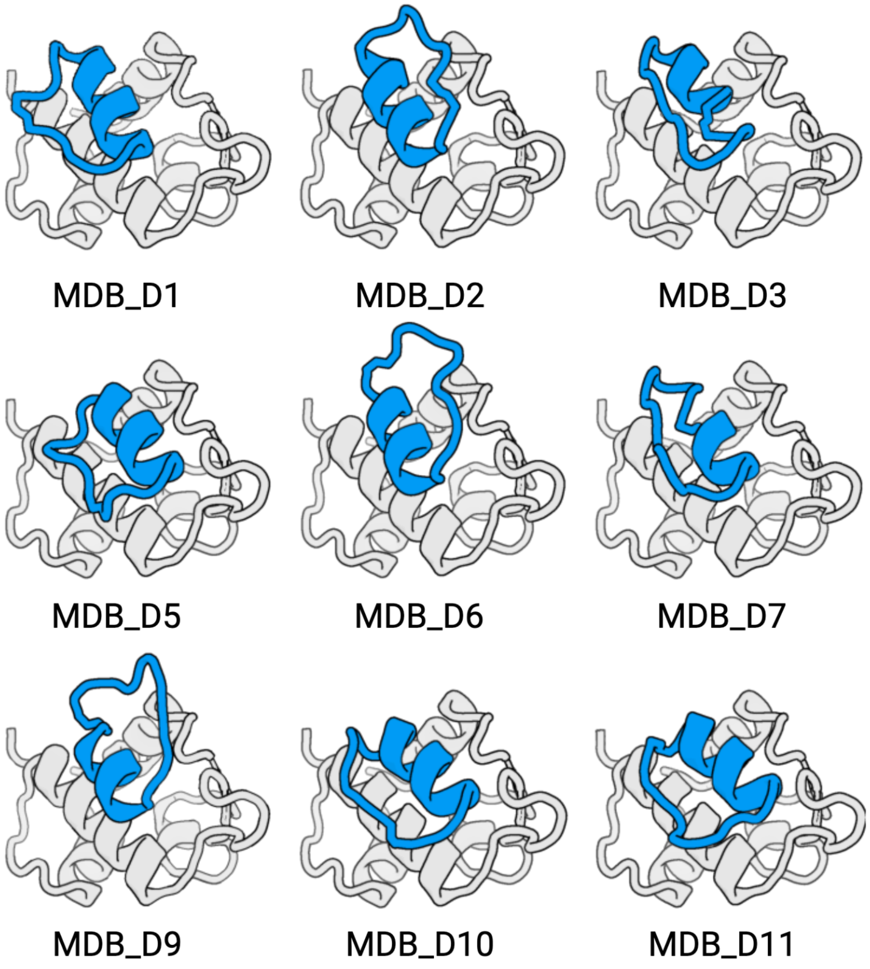

Supplementary Figure 8: Structural diversity of the selected MDM2 binding designs
Computational models for 11 putative MDM2–binding macrocycles selected after filtering using AfCycDesign (normalized iPAE < 0.3) and Rosetta interface metrics (ddG < –50 kcal/mol, SAP < 35, CMS > 300 Å^2). The designs that passed the computational filters were sorted by ddG, and the top 11 designs were chosen for synthesis.

Supplementary Table 2a: Sequences and interacting residues for selected MDM2 designs

Design	Sequence	Length	Interacting Residues (< 4 Å)	Net Charge (pH 7.4)*	Hydrophobicity %**
MDB_D1	KKYNWMVDELVSMVGKPE	18	M50,K51,L54,F55,L57,G58,Q59,I61,M62,Y67,Q72,H73,V75,F91,V93,K94,H96,I99,Y100,I103	-0.45	0.389
MDB_D2	EESEDEDFRVFERVLGI	17	M50,K51,L54,F55,L57,G58,Q59,I61,M62,Y67,Q72,H73,V75,F91,V93,K94,H96,I99,Y100,I103	-5.33	0.353
MDB_D3	RELMEMVGEKYDQNFIL	17	M50,K51,L54,F55,L57,G58,Q59,I61,M62,Y67,Q72,H73,V75,F91,V93,K94,H96,I99,Y100,I103	-2.44	0.412
MDB_D4	IESDDDFKVLTDVMGLD	17	M50,K51,L54,F55,L57,G58,Q59,I61,M62,Y67,Q72,H73,V75,F91,V93,K94,H96,I99,Y100,I103	-5.44	0.412
MDB_D5	DGSEFSKIWKSLGGDQDL	18	K51,L54,F55,L57,G58,Q59,I61,M62,Y67,Q72,H73,V75,F86,F91,V93,K94,H96,I99,Y100,I103	-2.45	0.222
MDB_D6	EDYQVLHDVLGLPLEFDD	18	M50,K51,L54,F55,L57,G58,Q59,I61,M62,Y67,Q72,H73,V75,F91,V93,K94,H96,I99,Y100,I103	-6.29	0.444
MDB_D7	MAMVGEELEGADFVLREL	18	M50,K51,L54,F55,L57,G58,Q59,I61,M62,Y67,Q72,H73,V75,F91,V93,K94,H96,I99,Y100,I103	-4.71	0.556
MDB_D8	SRKAKNKFEELWNELIDP	18	K51,L54,F55,L57,G58,I61,M62,Y67,Q72,H73,V75,F91,V93,K94,H96,I99,Y100	-0.75	0.333
MDB_D9	FKVLDEVLGVDVEDADEL	18	M50,K51,L54,F55,L57,G58,Q59,I61,M62,Y67,Q72,H73,V75,F91,V93,K94,H96,I99,Y100,I103	-6.44	0.500
MDB_D10	PGTPFAKEWSKMAGGAPL	18	K51,L54,F55,L57,G58,Q59,I61,M62,Y67,Q72,H73,V75,F86,F91,V93,K94,H96,I99,Y100,I103	0.9	0.500
MDB_D11	GLDLETDSEFAKEWQKMV	18	K51,L54,F55,L57,G58,Q59,I61,M62,Y67,Q72,H73,V75,F91,V93,K94,H96,I99,Y100,I103	-3.44	0.333

*Net charge calculated using BioPython(Cock et al. 2009)

Bio.SeqUtils.ProtParam.charge_at_pH

**Percent of sequence that is A,V,I,L,M,F,P

Supplementary Table 2b: Metrics for selected MDM2 designs

Design	Contact Molecular Surface ($Å^2$)	ddG (kcal/mol)	SAP	iPAE
MDB_D1	444.635	-51.979	29.699	0.1784
MDB_D2	452.577	-51.904	27.833	0.1316
MDB_D3	443.107	-51.644	29.691	0.1174
MDB_D4	458.674	-51.585	28.002	0.1333
MDB_D5	437.787	-50.908	32.035	0.1018
MDB_D6	428.573	-50.636	33.978	0.1571
MDB_D7	439.16	-50.596	28.72	0.0983
MDB_D8	403.172	-50.556	28.27	0.1013
MDB_D9	405.265	-50.53	32.888	0.1509
MDB_D10	440.422	-50.392	31.369	0.1314
MDB_D11	420.361	-50.272	33.345	0.1506

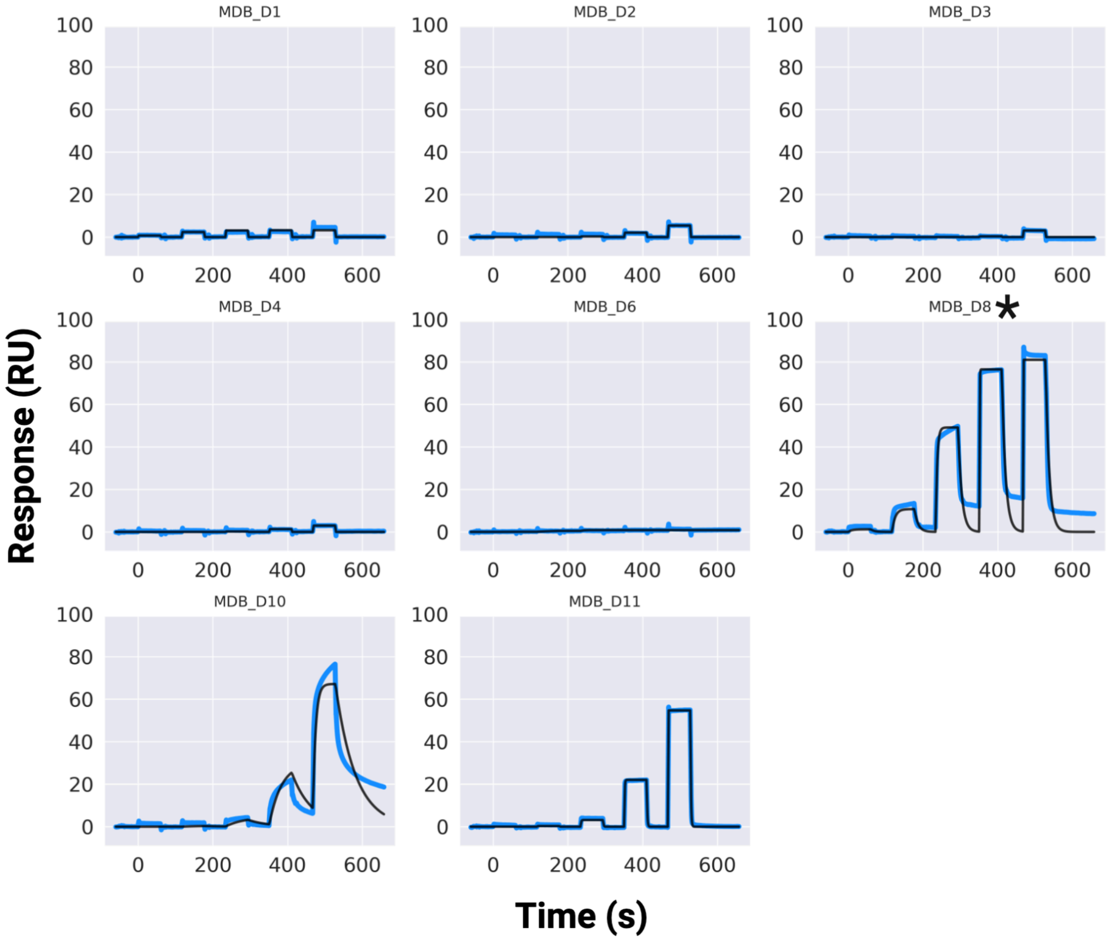

Supplementary Figure 9: SPR–based binding screen of the selected MDB designs against MDM2

Sensorgrams from an SPR single–cycle kinetics screen for the selected MDB designs, applying a 5–point 10–fold dilution with 100 μM as the highest concentration. Three designs (MDB_D8, MDB_D10, and MDB_11) show detectable binding at or below 100 μM. MDB_D8 denoted with * chosen for further characterization.

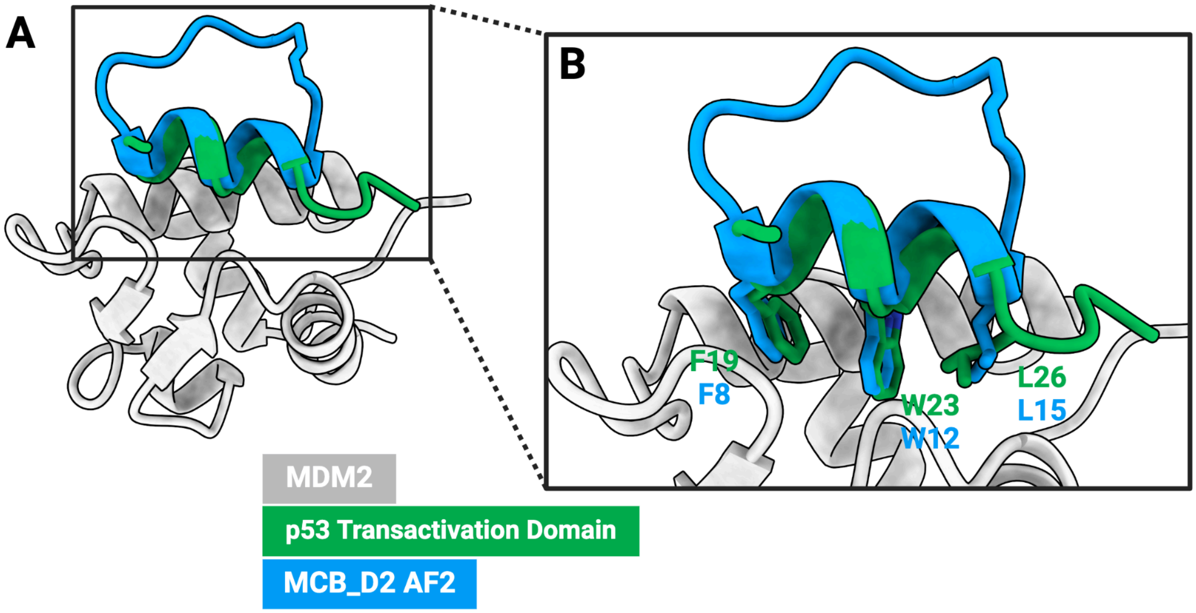

Supplementary Figure 10: MDB_D8 mimics p53 interactions

(A) Alignment of the MDB_D8 design model to the crystal structure of p53 bound to MDM2 (PDB: 1YCR[5]). (B) Positions and identities of interfacial side chains in MDB_D8 match those in the native ligand.

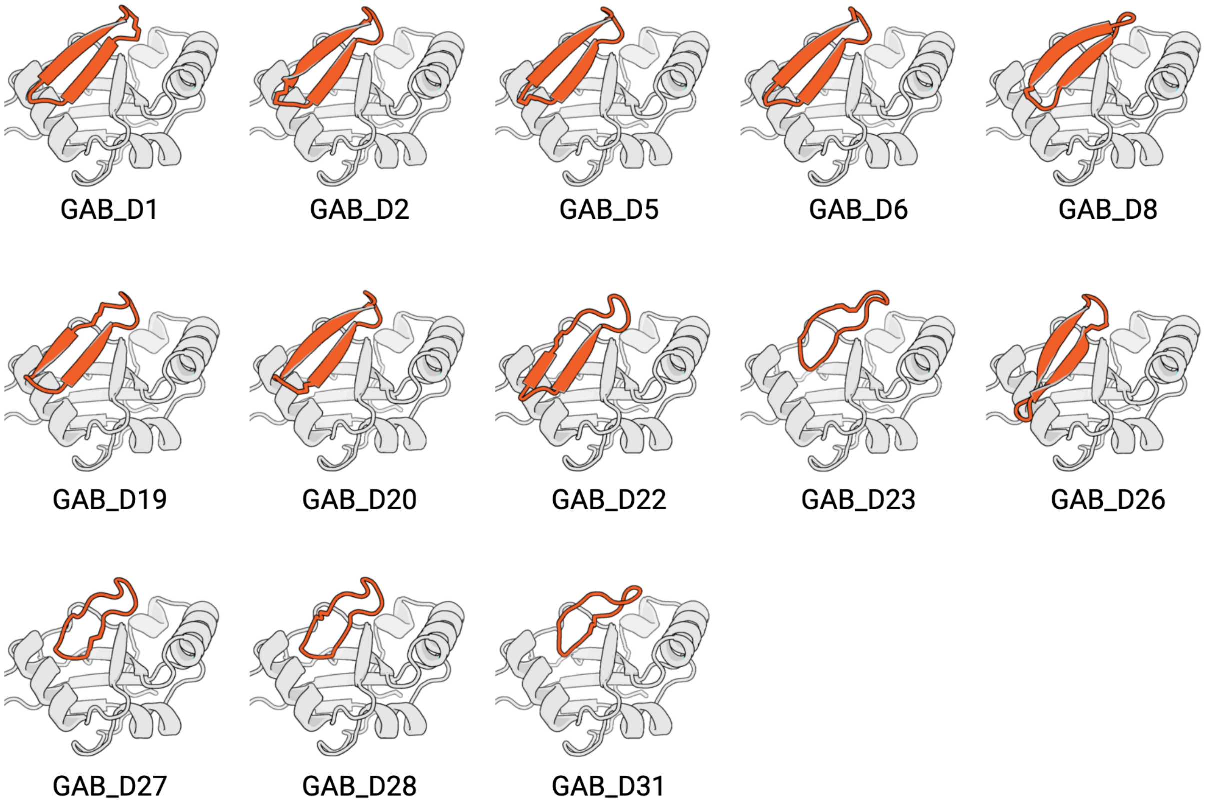

Supplementary Figure 11: Structural diversity of the selected GABARAP binding designs
Computational models for 13 putative GABARAP–binding macrocycles selected after filtering using AfCycDesign (normalized iPAE < 0.13 Å) and Rosetta interface metrics (ddG < –30 kcal/mol, SAP < 35, CMS > 300 Å^2). Post filtering, designs were clustered and 13 different designs from diverse clusters were selected for synthesis and experimental screening.

Supplementary Table 3a: Sequences and interacting residues for selected GABARAP designs

Design	Sequence	Length	Interacting Residues (< 4 Å)	Net Charge (pH 7.4)*	Hydrophobicity %**
GAB_D1	ETGEVENIDGVEIIYP	16	I23,R26,K48,K50,Y51,L52,V53,P54,F64,L65,K68	-5.33	0.375
GAB_D2	DGVTIIDIDTGEEEVL	16	I23,R26,K48,K50,Y51,L52,P54,L57,F64,L65,K68,R69	-6.44	0.375
GAB_D5	GIEIIELETGEVVVID	16	I23,R26,K48,K50,Y51,L52,P54,L57,L65,K68,R69	-5.44	0.500
GAB_D6	GEVEVIDGVEIIDLET	16	I23,R26,K48,K50,Y51,L52,P54,L57,L65,K68,R69	-6.44	0.438
GAB_D8	GSEYEEDGWTVLEPD	15	I23,K26,Y27,R30,K48,K50,Y51,L52,P54,Q61,F62,L65,R69,F102	-6.44	0.200
GAB_D19	KEKWDENIEIINIETGE	17	E19,I23,R30,K48,K50,Y51,L52,P54,L57,L65,K68	-4.44	0.235
GAB_D20	IEIIDLDTGEVEVWSPD	17	I23,R30,K48,K50,Y51,L52,P54,L57,F62,L65,R69	-6.44	0.412
GAB_D22	MELDGVKVLDIDTEEVV	17	I23,K26,Y27,R30,K48,K50,Y51,L52,V53,P54,L57,F64,L65,R69	-5.71	0.471
GAB_D23	LEDGWVDIETGKE	13	E19,I23,K26,Y27,R30,P32,V33,K48,K50,Y51,L52,V53,P54,F62,F106	-4.44	0.231
GAB_D26	LDTGEVYKAPNGQEVIE	17	I23,R30,K48,K50,Y51,L52,V53,P54,D56,L57,Q61,F64,L65,R69	-3.44	0.353
GAB_D27	IDIDTEEEVMPGV	13	I23,K26,R30,K48,K50,Y51,L52,V53,P54,L65	-5.44	0.462
GAB_D28	VMPGIIDIDTEEE	13	I23,K26,R30,K48,K50,Y51,L52,P54,F62,L65	-5.47	0.462
GAB_D31	DIVTDEELDGYV	12	E19,K26,Y27,R30,K48,K50,Y51,L52,V53,P54,F62,R69,F106	-5.44	0.333

*Net charge calculated using BioPython(Cock et al. 2009)

Bio.SeqUtils.ProtParam.charge_at_pH

**Percent of sequence that is A,V,I,L,M,F,P

Table 3b: Metrics for selected GABARAP designs

Design	Contact Molecular Surface (Å^2)	ddG (kcal/mol)	SAP	iPAE
GAB_D1	376.681	-33.816	21.883	0.1261
GAB_D2	392.525	-36.869	24.106	0.1294
GAB_D5	432.437	-50.38	31.061	0.1189
GAB_D6	429.527	-44.355	24.915	0.1196
GAB_D8	410.558	-37.819	12.733	0.1189
GAB_D19	414.294	-47.124	23.709	0.1290
GAB_D20	403.652	-44.449	30.475	0.1198
GAB_D22	441.999	-47.1	24.904	0.1109
GAB_D23	370.742	-46.269	16.574	0.1242
GAB_D26	456.39	-40.053	20.99	0.1106
GAB_D27	353.841	-33.906	20.753	0.1063
GAB_D28	358.797	-36.358	23.583	0.1066
GAB_D31	390.976	-44.605	17.368	0.1176

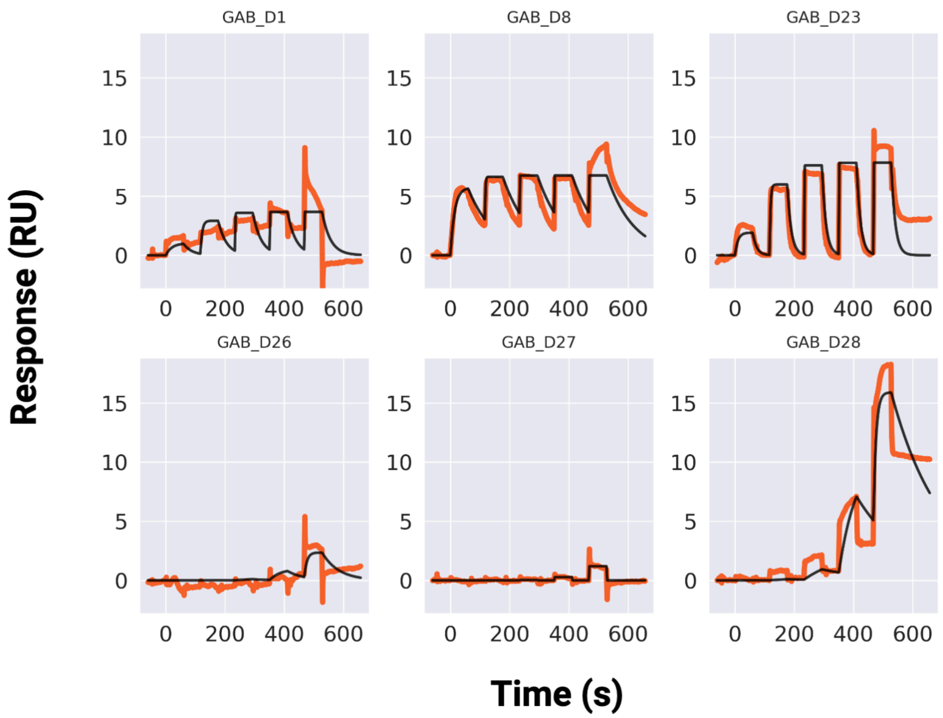

Supplementary Figure 12: SPR–based screening of the selected GAB designs against GABARAP

Sensorgrams from an SPR single–cycle kinetics screen for the selected GAB designs, applying a 5–point 10–fold dilution with 100 μM as the highest concentration. Four designs (GAB_D1, GAB_D8, GAB_D23, and GAB_D28) show detectable binding at or below 100 μM. GAB_D8 and GAB_D23 were chosen for further characterization.

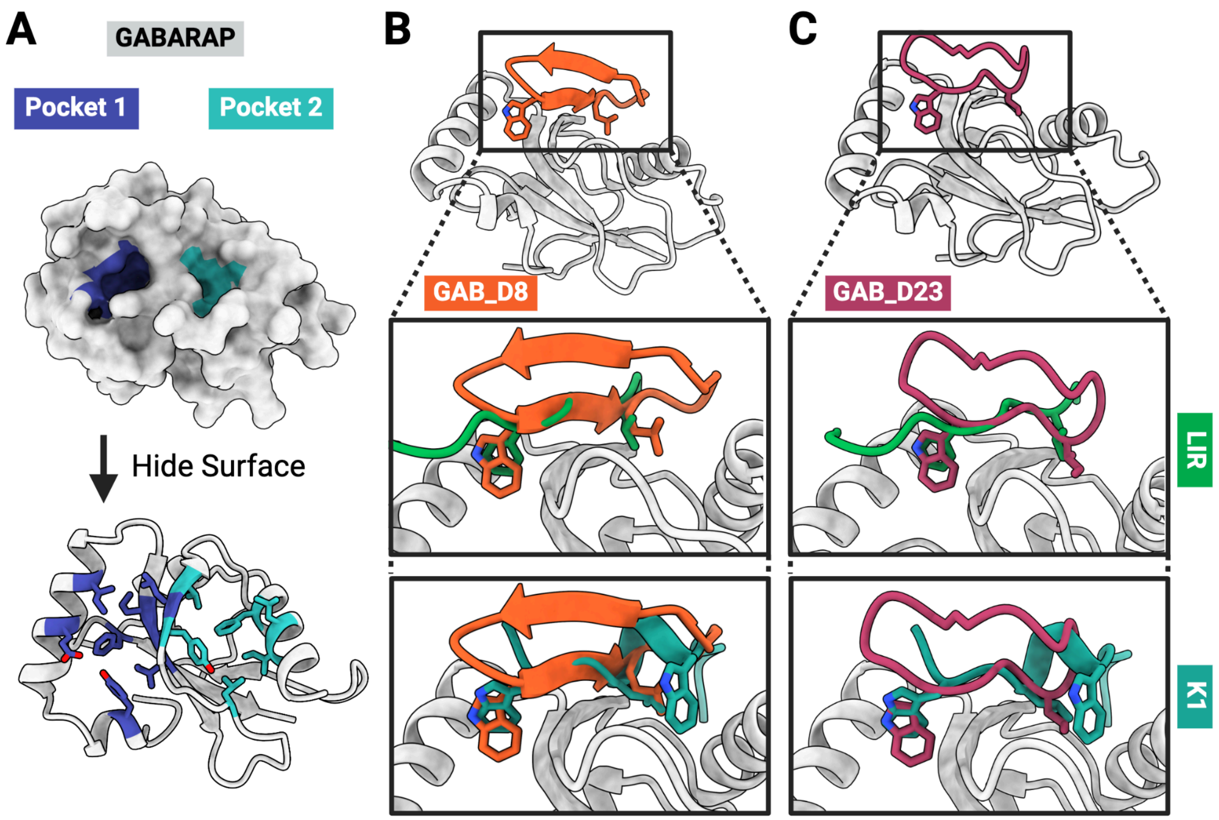

Supplementary Figure 13: GAB_D8 and GAB_D23 span both known pockets on GABARAP

(A) GABARAP has two hydrophobic pockets that are targeted by typical LIR peptides (exemplified by PDB entry 5LXI[3]) as well as the artificial ligand K1 (PDB: 3D32[6]). Both GAB_D8 (B) and GAB_D23 (C) interact with pocket 1 via an aromatic residue (tryptophan) and with pocket 2 via an aliphatic residue (leucine and isoleucine, respectively), thus mimicking the canonical binding mode found in native ligands. In contrast, the K1 peptide engages in the reverse orientation and inserts a second tryptophan side chain, in addition to a leucine, into pocket 2.

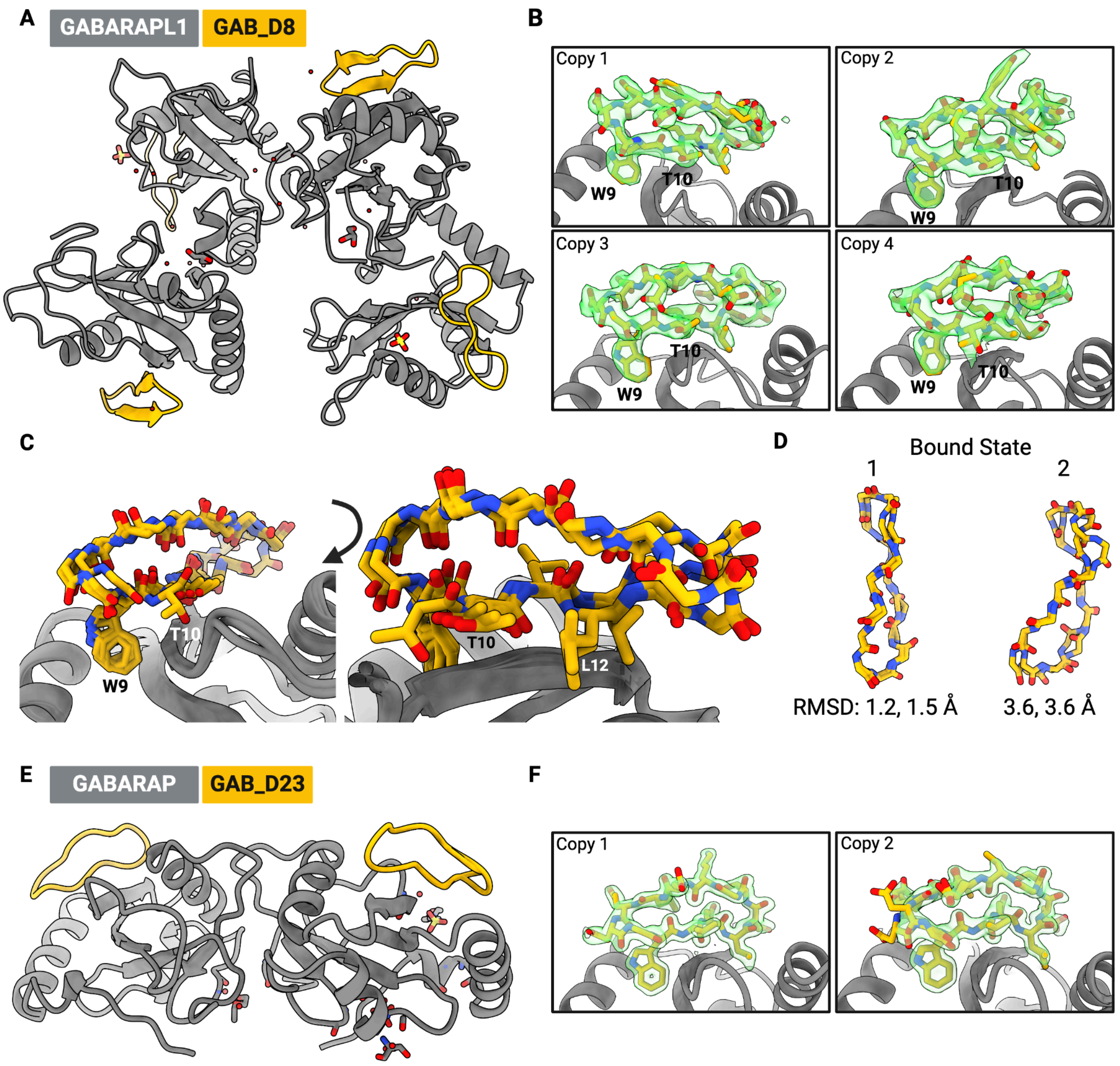

Supplementary Figure 14: GAB_D8 and GAB_D23 crystal structures display secondary conformations.

(A) The asymmetric unit contains four copies of GAB_D8 bound to GABARAPL1. (B) Electron densities (2Fo–Fc map at 1 σ contour level, green) are shown for each copy of the peptide. Note that copy 4 deviates from the other copies, presumably as a result of lattice restraints. Specifically, Thr10 and Val11 bulge out, establishing a new side chain–to–backbone hydrogen bond and a hydrophobic contact, respectively, with GABARAPL1 and causing a register shift in the strand pairing. (C) Alignment of all four models. The difference at Thr10 for copy 4 can be seen, as well as the register shift leading to Leu12 moving to the other side of the strand. (D)

The four copies can be grouped into two bound states. Both state 1 copies closely match the design with Cα RMSDs < 2 Å, but state 2 differs from the designed fold and contains copy 4 with its alternate binding mode. (E) The asymmetric unit contains two copies of GAB_D23 bound to GABARAP. (F) Electron densities (2Fo–Fc map at 1 σ contour level, green) are shown for each copy of the peptide. Note that copy 2 contains a second conformer to more completely account for the features of the electron density.

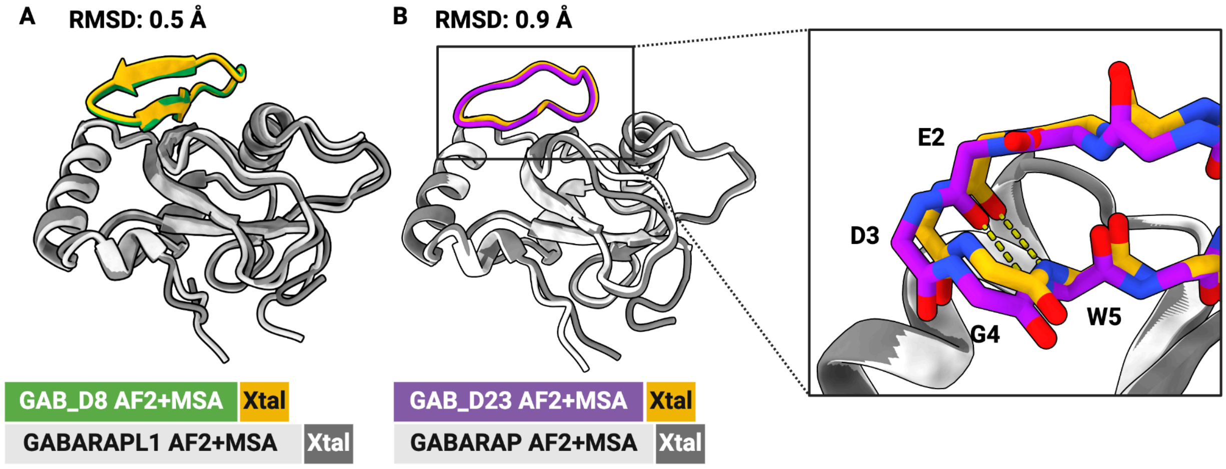

Supplementary Figure 15: Reprediction of GAB_D8 with GABARAPL1 and GAB_D23 with GABARAP using multiple sequence alignment (MSA) improves accuracy of the design models

(A) The GAB_D8–GABARAPL1 complex predicted with MSA aligned to the crystal structure. Cα RMSD calculated for the peptide aligned by the target chain. (B) The GAB_D23–GABARAP complex predicted with MSA aligned to the prevailing conformer found in the crystal structure. Cα RMSD calculated for the peptide aligned by the target chain. The close–up view shows capture of the type I' β–turn from the crystal structure in the MSA prediction.

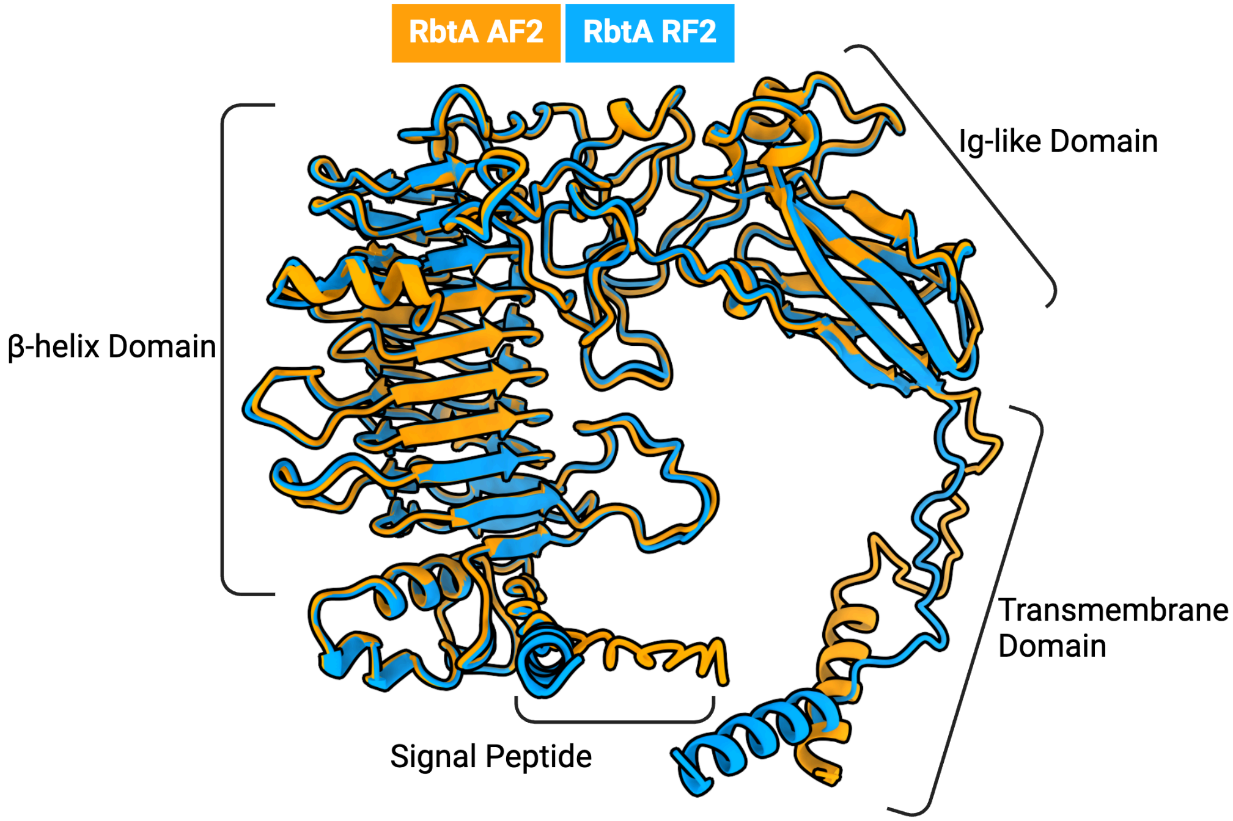

Supplementary Figure 16: Alignment of the RbtA structures predicted by RF2 and AF2
Alignment of RF2 (blue) and AF2 (orange) predictions of full length RbtA from *Acinetobacter baumannii.* The predicted structures from AF2 and RF2 are very similar, with a Cα RMSD excluding the transmembrane domain and signal peptide of 0.5 Å.

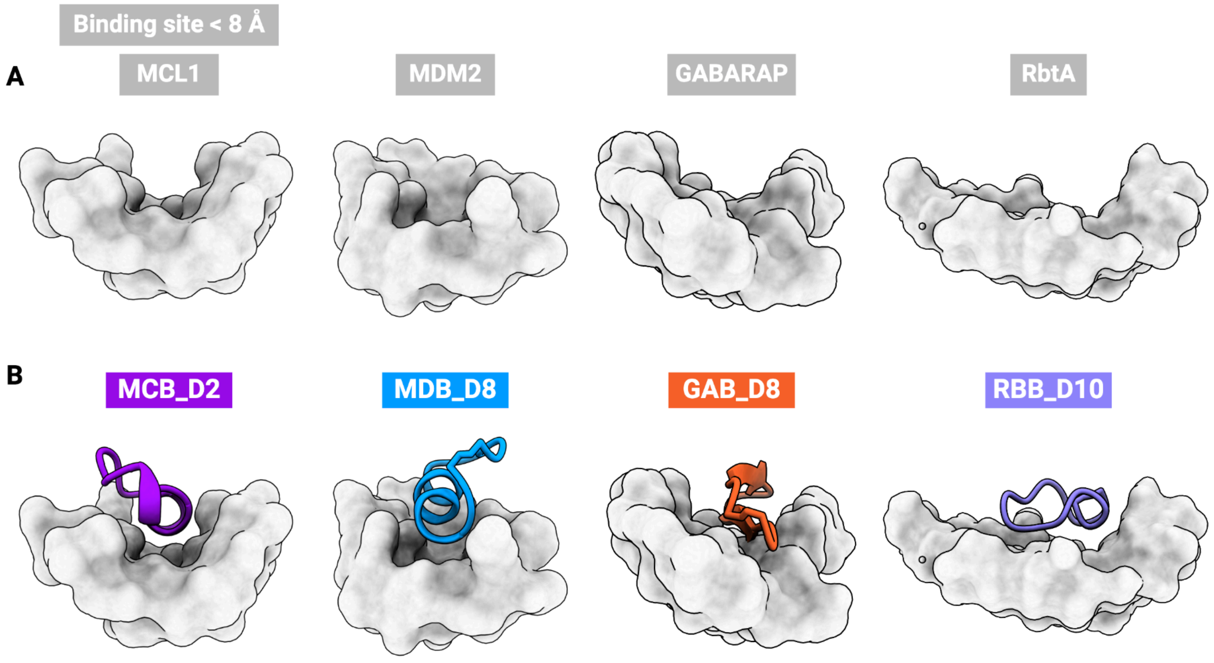

Supplementary Figure 17: Macrocycles designed by RFpeptides engage diverse target pockets with with appropriately shape–matched macrocycle comformations
(A) Atoms within 8 Å of the binder represented as surface for each of the targets described in this work. MCL1 and MDM2 have deep concave pockets, GABARAP has a relatively more open binding site, and RbtA has a very flat surface at the binding site. (B) Computationally–designed macrocycle binders with highest affinity shown in cartoon mode. The deep concave pockets of MCL1 and MDM2 are engaged by helix–containing macrocycles, while the more open, flat surfaces of GABARAP and RbtA are engaged by macrocycles with β–sheet and loop–like conformations, respectively.

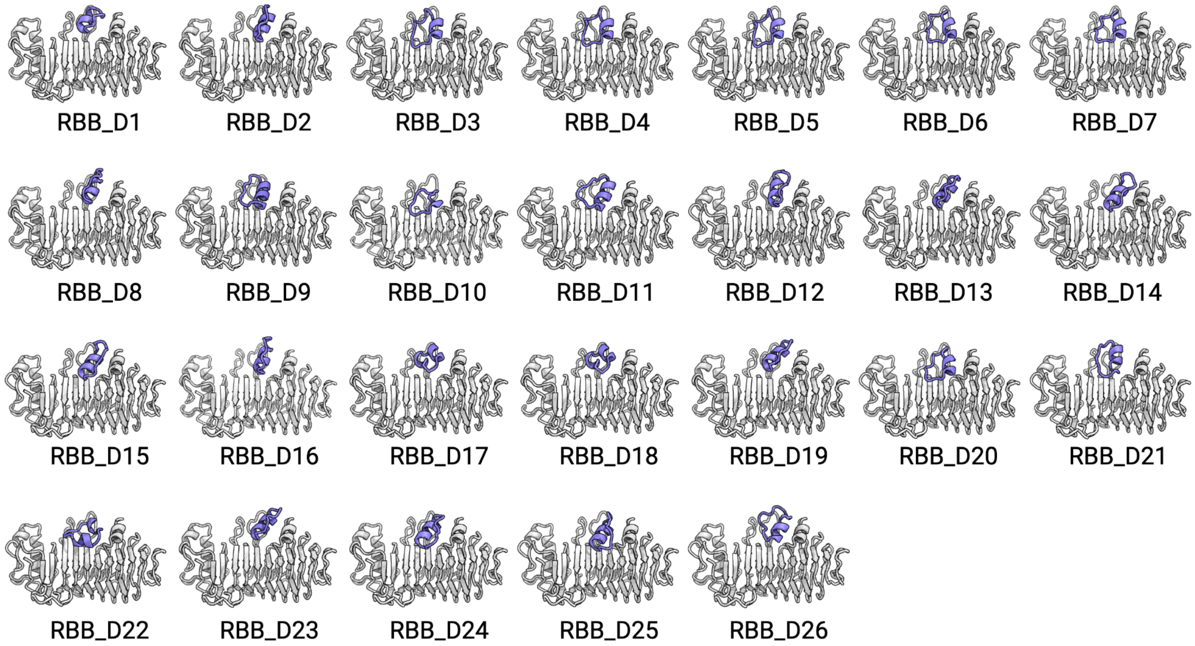

Supplementary Figure 18: Structural diversity of the selected RbtA binding designs
We diffused macrocyclic peptides against RbtA with hotspots L144, F202, F204, Y206, V208, L231, and A269 (based on NCBI GenBank entry PXA52927.1). Of the 80,000 design models, 147 were predicted to fold and bind as designed with high confidence by AfCycDesign (normalized iPAE < 0.4 and 1.5 Å Cα RMSD to the design model). We next used Rosetta to calculate binding affinity (ddG), spatial aggregation propensity (SAP) of the designed macrocycle, and the molecular surface area of the interface contacts (CMS). We generated two lists of candidates, one using Rosetta metrics ddG < –40 kcal/mol, SAP < 35, CMS > 300 Å^2, and the other of designs with iPAE < 0.28. The two lists were merged leaving 26 designs spanning those with the best Rosetta metrics, those with the best AfCycDesign metrics, and those with both.

Supplementary Table 4a: Sequences and interacting residues for selected RbtA designs

Design	Sequence	Length	Interacting Residues (< 4 Å)	Net Charge (pH 7.4)*	Hydrophobicity %**
RBB_D1	SSLPEYVRDVLGV	13	A139,S140,K170,A195,S196,K197,D199,R200,F202,F204,L229,D258,S260,E263,Y264,Q267,A269,I270	-1.75	0.462
RBB_D2	GKPGDSPWEKIESVVFG	17	S140,K170,A195,S196,K197,D199,R200,F202,F204,Y206,L229,L231,S260,E263,Y264,Q267,R268,A269,I270	-1.44	0.353
RBB_D3	PDPAIPENVQKVFKEHML	18	A139,S140,S142,L144,K170,T172,A195,K197,R200,F202,F204,Y206,L229,L231,E263,Y264,Q267,R268,A269,I270	-1.06	0.556
RBB_D4	PDPAFPENVQKVFKDKIE	18	A139,S140,S142,L144,K170,L171,T172,A195,K197,R200,F202,F204,Y206,L229,L231,D258,E263,Y264,Q267,R268,A269,I270	-1.1	0.500
RBB_D5	PDPAFPESIHKVFKDKII	18	A139,S140,S142,L144,K170,L171,T172,A195,S196,K197,R200,F202,F204,Y206,L229,L231,D258,E263,Y264,Q267,R268,A269,I270	-0.07	0.556
RBB_D6	FSKYEPVDDSYPEGIRKV	18	A139,S140,S142,K170,L171,T172,A195,S196,K197,R200,T201,F202,F204,Y206,L229,L231,D258,S260,E263,Y264,Q267,A269,I270	-1.45	0.333
RBB_D7	FEKYDPMDESYPENIRKV	18	A139,S140,S142,K170,L171,T172,A195,S196,K197,R200,T201,F202,F204,Y206,L229,L231,F256,D258,S260,E263,Y264,Q267,R268,A269,I270	-2.45	0.333
RBB_D8	VEGNDKGSDIYEFIRDK	17	A139,S140,K170,A195,S196,K197,R200,T201,F202,G203,F204,L229,L231,F256,D258,S260,E263,Y264,Q267,A269,I270	-2.48	0.235
RBB_D9	VDDAKEQVLAWAKDPTVV	18	A139,S140,K170,A195,S196,K197,D199,R200,F202,F204,L229,L231,S260,E263,Y264,Q267,R268,A269,I270	-2.48	0.500
RBB_D10	KLFGPDPYLPENVQ	14	T97,S142,L144,K170,T172,F2	-1.45	0.500

			02,F204,Y206,L229,L231,E263,Y264,Q267,R268,A269,I270		
RBB_D11	SISGEPEVFKKVYSIAGI	18	A139,S140,S142,K170,L171,T172,A195,S196,K197,D199,R200,T201,F202,F204,Y206,L229,L231,S260,E263,Y264,Q267,R268,A269,I270	-0.75	0.444
RBB_D12	VEGKKATSKDPWKVLEDY	18	S140,K170,A195,S196,K197,D199,R200,F202,F204,Y206,Q227,L229,L231,F256,D258,S260,E263,Y264,Q267,R268,A269,I270	-0.49	0.278
RBB_D13	VEASWRAREVLEFLGKS	17	S142,K170,L171,T172,A195,S196,K197,D199,R200,F202,F204,Y206,L229,L231,F256,D257,D258,S260,E263,Y264,Q267,R268,A269,I270	-0.48	0.412
RBB_D14	SVAKEIAEWIGIPSKVPP	18	S142,V143,K170,L171,T172,A195,F202,F204,Y206,L229,L231,E263,Y264,V266,Q267,R268,A269,I270	-0.75	0.556
RBB_D15	SLAKEIADWIGIPSSVPP	18	S142,V143,K170,L171,T172,A195,F202,F204,Y206,L229,L231,F256,E263,Y264,Q267,R268,A269,I270	-1.75	0.556
RBB_D16	VRGEKTTPDPWEFIYNL	17	A139,S140,K170,A195,S196,K197,S198,R200,T201,F202,F204,L229,L231,F256,D258,S260,E263,Y264,Q267,A269,I270	-1.48	0.353
RBB_D17	FAPVFAKYDPNLAD	14	A139,S140,F141,K170,A195,S196,K197,R200,T201,F202,F204,Y206,L229,L231,D258,S260,E263,Y264,Q267,A269,I270	-1.45	0.643
RBB_D18	FAPVFAKYDPNLAE	14	A139,S140,F141,K170,T172,A195,S196,K197,R200,T201,F202,F204,Y206,L229,L231,D258,S260,E263,Y264,Q267,A269,I270	-1.44	0.643
RBB_D19	FEASSEVAKQLGEVVG	16	S140,K170,A195,S196,K197,R200,F202,F204,L229,F256,D258,S260,E263,Y264,Q267,A269,I270	-2.44	0.438

RBB_D20	FDKYDPLDDLYPENIRKV	18	A139,S140,S142,L144,K170,L171,T172,A195,S196,K197,R200,T201,F202,F204,Y206,L229,L231,F256,D258,S260,E263,Y264,Q267,R268,A269,I270	-2.45	0.389
RBB_D21	FRLIDKKLGIEDNDPIEA	18	A139,S140,S142,K170,L171,T172,A195,S196,K197,D199,R200,F202,F204,Y206,L229,L231,S260,E263,Y264,Q267,A269,I270	-2.44	0.444
RBB_D22	RKPVEEILKKNEITYEDV	18	P95,A139,S140,F141,S142,K170,T172,A195,S196,K197,R200,F202,F204,Y206,L229,L231,D258,S260,E263,Y264,Q267,R268,A269	-1.45	0.333
RBB_D23	KPPASSEVARKVMEVFG	17	S140,K170,A195,S196,K197,D199,R200,F202,F204,L229,L231,F256,D258,S260,E263,Y264,Q267,R268,A269,I270	0.55	0.529
RBB_D24	KAKNWFTEQLSEVIPGI	17	A139,S142,K170,T172,A195,S196,K197,D199,R200,F202,F204,Y206,L229,L231,F256,S260,E263,Y264,Q267,R268,A269,I270	-0.45	0.412
RBB_D25	SGNPVLQIAAELDPNVKG	18	S140,S142,K170,L171,T172,A195,K197,F202,F204,Y206,L229,L231,T233,F256,S260,E263,Y264,Q267,R268,A269,I270	-1.75	0.500
RBB_D26	EFARLLAKGDFEGWKDSK	18	A139,S140,K170,A195,S196,K197,D199,R200,F202,F204,L229,F256,D258,S260,E263,Y264,Q267,A269,I270	-0.34	0.333

*Net charge calculated using BioPython(Cock et al. 2009)

Bio.SeqUtils.ProtParam.charge_at_pH

**Percent of sequence that is A,V,I,L,M,F,P

Supplementary Table 4b: Metrics for selected RbtA designs

Design	Contact Molecular Surface (Å^2)	ddG (kcal/mol)	SAP	iPAE
RBB_D1	343.196	-33.718	31.238	0.2616
RBB_D2	380.027	-38.673	29.894	0.2698
RBB_D3	407.954	-39.265	31.102	0.2327
RBB_D4	392.468	-34.502	25.634	0.2486
RBB_D5	415.948	-35.904	37.245	0.2387
RBB_D6	437.807	-40.145	30.59	0.2568
RBB_D7	467.614	-43.384	26.253	0.2242
RBB_D8	383.769	-38.72	24.318	0.2739
RBB_D9	366.257	-29.498	23.354	0.2406
RBB_D10	362.074	-32.855	33.742	0.1949
RBB_D11	429.721	-45.643	35.096	0.2036
RBB_D12	417.54	-39.329	24.265	0.2775
RBB_D13	456.631	-40.242	24.246	0.2525
RBB_D14	387.477	-44.656	41.066	0.2296
RBB_D15	336.422	-37.087	43.253	0.2612
RBB_D16	405.978	-38.653	36.337	0.2385
RBB_D17	474.038	-47.363	32.549	0.2636
RBB_D18	480.282	-48.513	32.567	0.2618
RBB_D19	370.075	-40.002	24.45	0.2075
RBB_D20	484.346	-47.842	34.434	0.3589
RBB_D21	392.999	-41.735	34.205	0.3193
RBB_D22	464.31	-40.435	24.141	0.3152
RBB_D23	425.067	-42.157	25.105	0.3260
RBB_D24	407.163	-40.406	34.046	0.3935
RBB_D25	401.154	-42.823	22.151	0.3517
RBB_D26	357.573	-40.61	24.136	0.3666

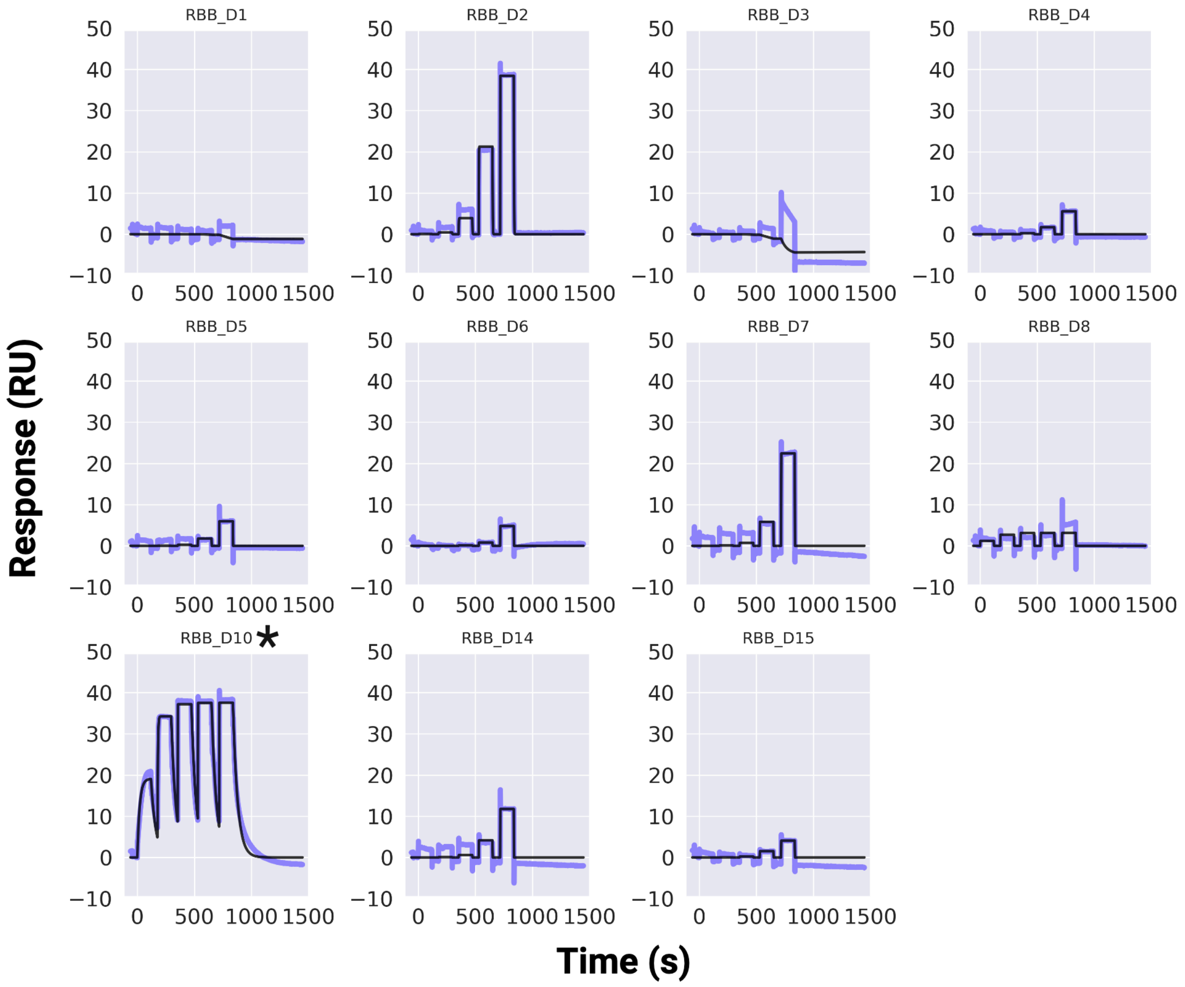

Supplementary Figure 19: SPR–based screening of the selected RBB designs against RbtA

Sensorgrams from an SPR single–cycle kinetics screen for the selected RBB designs, applying a 5–point 10–fold dilution with 100 μM as the highest concentration. Four designs (RBB_D2, RBB_D7, RBB_D10, and RBB_D14) show detectable binding at or below 100 μM. RBB_D10 denoted with * chosen for further characterization.

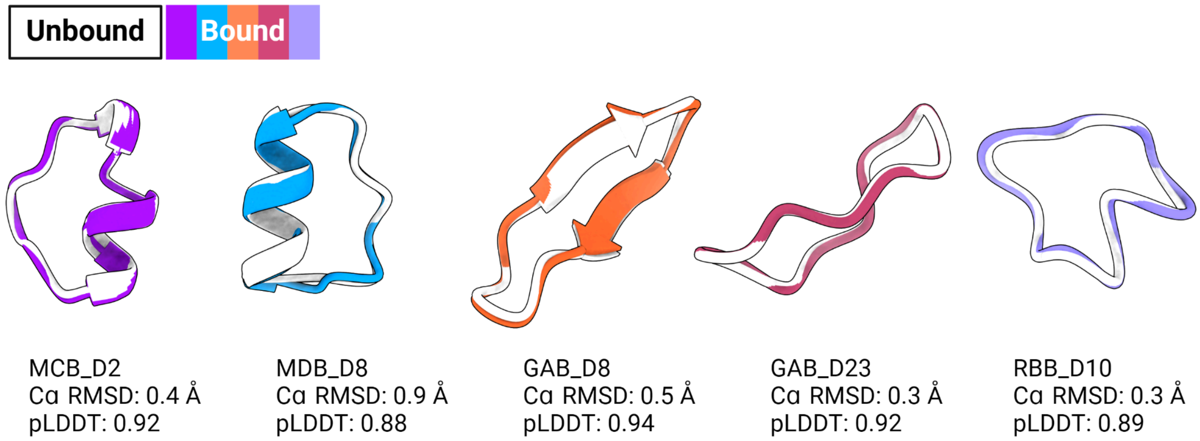

Supplementary Figure 20: Comparison of bound and unbound predictions for successful designs.

Successful designs with crystal structures from this work were compared to AfCycDesign predictions without the respective target protein present (white). All designs are predicted to be pre–folded in their bound state with high confidence.

2. Materials and Methods

2.1.1. Implementation of the cyclic relative position encodings for RoseTTAFold and RFdiffusion

While conceptually similar to cyclic positional encodings in AfCycDesign[7], our implementation of the cyclic relative position encodings differs in two ways. First, as shown in Figure 1A (e.g., first row), the relative position encoding for a starting residue ‘i’, and its increasingly distant neighbors ‘j’ going in the N→C direction increments by positive 1 at every next position. This positive increment occurs until the difference between i and j is such that j is more than halfway around the peptide from i, at which point the relative position encoding switches sign. With the switched sign, residue j is now seen by residue i to be to the “left” (N–terminal from i) instead of to the right (C–terminal from i). Upon switching the sign, the relative position encoding continues to be incremented by +1, encoding an increasingly close distance from j→i approaching from the left/N–term. When applied to all residues in the forward pass of the network, this manifests a polypeptide chain in which there are no termini and the backbone is cyclized. The second, more practical, difference between the relative position encodings demonstrated in Rettie et. al.[7] and those shown here is the way in which they are introduced to the neural network. In addition to introducing the cyclized offsets into the network pairwise representation, as in Rettie et. al., we include the same offsets into the edge features of the graph fed into the 3D track of RoseTTAFold. This then enables RF’s SE3Transformer to also see that the peptide should be cyclized.

All the required changes for implementing RFpeptides are now incorporated into the RFdiffusion codebase. The latest version of RFdiffusion can be downloaded from https://github.com/RosettaCommons/RFdiffusion.

2.1.2. Monomeric macrocycle prediction with RFpeptides

With cyclic relative positional encoding implemented in RoseTTAFold we used the same set of 80 NMR structures from the ProteinDataBank that was used in our previous work to validate predictions of diverse cyclic structures[7]. PDB entries were identified by searching for monomeric peptides less than 40 amino acids in length, containing only L amino acids, and with CONECT records between the terminal N and C atoms.

1bh4, 1df6, 1hvz, 1jbl, 1qvk, 1qvl, 1r1f, 1ski, 1skk, 1vb8, 1za8, 1znu, 2atg, 2b38, 2eri, 2gj0, 2k7g, 2kch, 2knm, 2kuk, 2kux, 2kvx, 2lam, 2ll5, 2lur, 2lws, 2lwt, 2lwu, 2lwv, 2lye, 2lyf, 2lzi,

2m77, 2m78, 2m79, 2m9o, 2mh1, 2mn1, 2mso, 2mt8, 2mw0, 2n07, 2nb5, 2ndl, 2ndm, 2ndn, 2ns4, 2otq, 2ox2, 2po8, 5h1h, 5h1i, 5kwz, 5kx1, 5wov, 5wow, 6dny, 6pin, 6pio, 6pip, 6u7q, 6u7r, 6u7s, 6wpv, 7f32, 7k7x, 7l53, 7l54, 7l55, 7lhc, 7m25, 7m27, 7m28, 7m29, 7m2a, 7m2b, 7m2c, 7m3u, 7rn3, 7s55

Multiple sequence alignments were generated by

```
python RoseTTAFold/examples/make_protein_msa.sh example.fasta
```

and the resulting `example.a3m` used for the input for prediction using RoseTTAFold

```
python RoseTTAFold/network/predict.py -inputs example.a3m -n_recycles
48 -cyclize
```

The outputs from RoseTTAFold are the highest pLDDT models for each input sequence and MSA. To determine their backbone heavy atom RMSD to the native structure the following RosettaScript was used with Rosetta v2024.16.

Example RosettaScript for calculating backbone heavy atom RMSD

```
<ROSETTASCRIPTS>
<SIMPLE_METRICS>
      <RMSDMetric name="RMSD"
            rmsd_type="rmsd_protein_bb_heavy"
            use_native="true"
            super="true"/>
</SIMPLE_METRICS>
<MOVERS>
      <PeptideCyclizeMover name="pcm"/>
      <RunSimpleMetrics name="run_metric" metrics="RMSD"/>
</MOVERS>
<PROTOCOLS>
      <Add mover="close" />
```

```
    <Add mover="run_metric" />
</PROTOCOLS>
</ROSETTASCRIPTS>
```

In cases where the native structure was an NMR ensemble, all states of the NMR structures were compared to the predicted model and the lowest RMSD was plotted in Supplementary Figure S1.

2.1.3. Monomeric macrocycle design with RFpeptides

To design a monomeric ordered macrocycle (i.e., not binder design) we simply add the cyclic positional encoding to RFdiffusion as described in 1.1.1 during an otherwise standard execution of RFdiffusion. Then, we use a sequence design model, such as ProteinMPNN or LigandMPNN to design the sequences. Finally, AfCycDesign[7] is used for structure prediction of the designed peptides.

For the experiment shown in Figures 1 B,C and S2, we first generated 200 backbones for macrocycles of length 8, 10, 12, 14, 16, 18. An example of the execution to generate the peptide backbones is:

```
python rf_diffusion/run_inference.py
inference.output_prefix=/path/to/folder/file_prefix
inference.num_designs=200 contigmap.contigs=[\"8-8\"]
inference.cyclic=True inference.cyc_chains="a" diffuser.T=50
```

The new flags `inference.cyclic=True` and `inference.cyc_chains=”a”` trigger the addition of cyclized offset features to the network. For each of these backbones we then used LigandMPNN with sampling temperature of 0.1 to design 8 sequences per backbone. AfCycDesign was then used to predict the structure of each of these sequences.

2.1.4. tSNE dimensionality reduction and gaussian mixture model clustering

For the experiment described in the section 2.1.3, we computed an all–by–all (1200x1200) matrix of TMscores, in which the (i,j) entry is the average of the target– and query–normalized TMscores output from TMalign[8] between peptides i and j. We then used tSNE[9] implemented in Scikit–Learn[10] to reduce the dimensionality of this matrix to 1200x2. We then plotted this matrix,

colored by various quantities of interest (e.g., peptide length, scRMSD; Fig S2 D,E). We clustered the points in this matrix using Scikit–Learn's Gaussian mixture model clustering implementation[10]. To ensure meaningful clustering assignments, we computed intra–cluster mean TMscores as a function of the number of clusters being fit to the data (Fig S2 C). Intuitively, as the peptides are split into more and more clusters, the intra–cluster mean TMscore rises.

2.1.5 Measuring self–consistency of designs and MaxCluster clustering

A useful benchmark for polypeptide backbone diffusion models is a measurement of the frequency with which a sampled polypeptide can be assigned an amino acid sequence (usually with ProteinMPNN[11]) that, when "re–folded" using a protein structure prediction network (such as AlphaFold[12], ESMFold[13] or RoseTTAFold[14]), recapitulates the original sampled backbone with high confidence. Such computational filtering criteria have been shown to yield experimentally active designs for a variety of functions[15,16], and is commonly known as the "self–consistency" test. Furthermore, it is useful to quantify the number of *unique* protein or peptide "folds" produced by a generative model that achieve the aforementioned metrics. This additional criteria separates models which consistently produce a single or few unique solutions to a problem from models which produce a multitude of high quality solutions.

To compute this benchmark for RFpeptides, we employed the following pipeline: Using the structures and sequences generated in section 2.1.3 above, we computed the fraction of backbones at each length which have at least 1 of the 8 LigandMPNN sequences re–fold with AfCycDesign to within 2.0 Å RMSD and with at least 0.8 (out of 1.0) pLDDT. In Figure 1C, this statistic is denoted in the panel legend as "all" in blue. Additionally, to quantify the rate of unique structural clusters at each length which are self–consistent, we used MaxCluster[17,18] to structurally cluster all 1200 (6 lengths, 200 structures each) backbones sampled. We then redundancy–reduced the set of passing backbones on a per–length basis according to MaxCluster structural cluster assignments, so as not to double count multiple successes from the same peptide structural family. This new statistic is at most equivalent to the aforementioned "raw" success rate (but likely lower) and is denoted in the Figure 1C panel legend as "unique @ TM 0.5" in orange. It can be interpreted as the probability that a backbone sampled from RFpeptides will be a structurally unique, self–consistent design when sampling a total of 200 backbones at a given length. We used a TMscore cutoff of 0.5 and a hierarchical clustering strategy, executed on the command line as follows:

```
/path/to/maxcluster -l $list_of_pdbs -in -TM -C 2 -Tm 0.5
```

2.2. Macrocycle Binder Design with RFpeptides

Generally, designing macrocyclic binders to selected targets with RFpeptides is a four–step process. First, we generate macrocyclic peptide backbones with diverse sizes (e.g., sampling a number of designs from length 8 to 18 uniformly) against the selected target protein. This backbone process can also be guided towards a specified list of 'hotspot' residues on the target. Next, amino acid sequences are designed for each generated backbone using iterative rounds of ProteinMPNN[11] and Rosetta FastRelax[19,20]. Third, the designed sequences are re–predicted using AfCycDesign and RF2 to confirm if the designed sequences fold and bind in the designed conformations. Finally, the downselected designs from AfCycDesign and RF2[7,21] are further filtered using Rosetta–based interface metrics.

2.2.1 Macrocycle Backbone Generation

To prepare the target structure for macrocycle backbone generation, the PDB files corresponding to the selected target were downloaded from the Protein Data Bank and stripped of all water and ligands, leaving just the target protein atoms. For medium to large–sized target proteins (> 300 amino acids), the target protein can also be truncated to keep just the relevant target domains to reduce the computational costs. An example script for macrocycle backbone generation is shown below. It generates 400 macrocyclic peptides (controlled by the `inference.num_designs)` ranging from 12 to 18 amino acids against residues 1–113 of the target protein defined as chain A. The `contigmap.contigs` option defines the size range as 12–18 residues for the diffused cyclic peptide against residues 1–113A of a target protein as chain A from `inference.input_pdb`. The `inference.cyclic` option implements cyclic relative positional encoding and `inference.cyc_chains='a'` applies it just to the diffused peptide that will be chain A in the output pdb file. The number of time steps are controlled using `diffuser.T=50` setting them to 50. The `ppi.hotspot_res` option defines the target hotspot residues to guide the macrocycle generation. In the example below, the macrocycles are generated to bind around the residue numbers 68 and 70 of the target chain A. This line can be omitted if no hotspots are to be used. The final output will have a diffused cyclic peptide 12–18 residues as chain A and the residues 1 to 113 of the input target protein shifted to chain B.

Example script for generating macrocyclic binders against a target protein

```
prefix=./outputs/diffused_binder_cyclic
pdb='./target.pdb'
num_designs=400
script="rf_diffusion/run_inference.py"
python $script --config-name base \
      inference.output_prefix=$prefix \
      inference.num_designs=$num_designs \
      contigmap.contigs=[\'12-18,0\ A1-113,0\'] \
      inference.input_pdb=$pdb \
      inference.cyclic=True \
      diffuser.T=50 \
      +inference.cyc_chains='a' \
      +ppi.hotspot_res=[\'A68\',\'A70\'] \
```

For MCL1 and MDM2 designs, we generated 10,000 backbones without defining any hotspot residues. For targeting GABARAP and RbtA, we generated 20,000 backbones with hotspots specified. For designing against GABARAP, we defined six hotspot residues: Tyr51, Leu52, Lys50, Lys48, Phe62, and Leu65 (residue numbering the same as PDB ID: 7ZKR[22]). For targeting RbtA, we used the following seven hotspot residues: Leu144, Phe202, Phe204, Tyr206, Val208, Leu231, and Ala269 (residue numbering based on NCBI GenBank entry PXA52927.1). We explored macrocycles of length 16 residues against MCL1, lengths 16–18 residues for MDM2, and 12–18 for GABARAP and RbtA. Example scripts and files are available as Supplementary files.

2.2.2. Sequence Design

Amino acid sequences for the designed backbones were designed using four iterative rounds of ProteinMPNN[11] followed by Rosetta FastRelax[20]. ProteinMPNN can be downloaded from https://github.com/dauparas/ProteinMPNN. An example Rosetta FastRelax script is shown below and is also available in Supplementary Files. This example uses the output from the example script for generating macrocyclic binders against a target protein, `diffused_binder_cyclic_1.pdb`

.

We used the following ProteinMPNN options:

```
--pdb_path ./diffused_binder_cyclic_1.pdb
--pdb_path_chains A
--temperature 0.0001
--backbone_noise 0
--omit_AAs C
--num_seq_per_target 1
--path_to_model_weights vanilla_model_weights/v_48_020.pt
```

We chose to omit cysteine from our designs to streamline synthesis and validation of the peptides as disulfides or singular cysteines would require additional steps in synthesis. After designing the sequence with ProteinMPNN, the input model was updated with the new sequence, and the structure was minimized with FastRelax. During the relax step, distance, angle, and dihedral constraints were applied to maintain the terminal bond using Rosetta PeptideCyclizeMover as described previously[1,23]. The relax step was implemented as a PyRosetta4[24] (Version 2022.41) script that imports the PeptideCyclizeMover and FastRelax as XmlObjects from the following RosettaScripts.

An example RosettaScripts fast_relax.xml used for PeptideCyclizeMover and FastRelax movers

---

```
<ROSETTASCRIPTS>
<SCOREFXNS>
<ScoreFunction name="beta" weights="beta_nov16">
       <Reweight scoretype="metalbinding_constraint" weight="1.0" />
       <Reweight scoretype="arg_cation_pi" weight="3" />
              <Reweight scoretype="approximate_buried_unsat_penalty" weight="5”/>
              <Set approximate_buried_unsat_penalty_burial_atomic_depth="3.5" />
              <Set approximate_buried_unsat_penalty_hbond_energy_threshold="-0.5" />
              <Reweight scoretype="coordinate_constraint" weight="1" />
              <Reweight scoretype="atom_pair_constraint" weight="1" />
              <Reweight scoretype="dihedral_constraint" weight="1" />
              <Reweight scoretype="angle_constraint" weight="1" />
       </ScoreFunction>
<RESIDUE_SELECTORS>
       <Chain name="chA" chains="A"/>
       <Neighborhood name="int" selector="chA" distance="8"/>
```

```
		<Not name="target" selector="chA"/>
		<And name="target_int" selectors="target,int" />
		<Not name="not_target_int" selector="target_int" />
		<And name="non_interface_target" selectors="not_target_int,target"/>
</RESIDUE_SELECTORS>
<TASKOPERATIONS>
		<OperateOnResidueSubset name="move_interface" selector="target_int" >
			<RestrictToRepackingRLT/>
		</OperateOnResidueSubset>
		<OperateOnResidueSubset name="hold_non_interface" selector="non_interface_target" >
			<PreventRepackingRLT/>
		</OperateOnResidueSubset>
</TASKOPERATIONS>
<MOVERS>
		<PeptideCyclizeMover name="pcm" residue_selector="chA"/>
		<FastRelax name="full_relax_complex"
			repeats="3"
			scorefxn="beta"
			min_type="dfpmin_armijo_nonmonotone"
			ramp_down_constraints="false"
			task_operations="move_interface,hold_non_interface">
			<MoveMap name="specifics">
				<Jump number="1" setting="true" />
				<ResidueSelector selector="non_interface_target" chi="0" bb="0" />
				<ResidueSelector selector="target_int" chi="1" bb="0" />
			</MoveMap>
		</FastRelax>
</MOVERS>
<PROTOCOLS>
		<Add mover="pcm" />
		<Add mover="full_relax_complex"/>
</PROTOCOLS>
<OUTPUT />
</ROSETTASCRIPTS>
```

PyRosetta script to relax the macrocycle–bound target protein

---

```
from pyrosetta import *
init('-beta_nov16')
```

```
xml = 'fast_relax.xml'
objs = protocols.rosetta_scripts.XmlObjects.create_from_file(xml)
fr = objs.get_mover('full_relax_complex')
pcm = objs.get_mover('pcm')
pose = pose_from_pdb('diffused_binder_cyclic_1_mpnn.pdb')
pcm.apply(pose)
fr.apply(pose)
pcm.apply(pose)
pose.dump_pdb('diffused_binder_cyclic_1_mpnn1.pdb')
```

After relaxing the design with the ProteinMPNN generated sequence, the new model was designed again using ProteinMPNN, and this cycle was repeated. Together, this process generated four sequences for each backbone generated by the diffusion step. Overall, for MCL1 and MDM2, we generated 39,860 and 40,000 designs, respectively. 80,000 designed sequences were generated for GABARAP and RbtA.

2.3. Target–macrocycle complex structure prediction using AfCycDesign

We used AfCycDesign version 1.1.1 to predict the structure of designed cyclic peptides in complex with their target. An example script to run AfCycDesign for macrocycle–bound target is described below and can also be run using ColabDesign at https://colab.research.google.com/github/sokrypton/ColabDesign/blob/main/af/examples/af_cyc_design.ipynb

In the example below, the target protein is chain A. No templates were used for the designed macrocycle. The structure of the target chain in the input model was used as a template while predicting the co–complex structure. The outputs from this step is a pdb file of the predicted binding mode of the designed peptide, and a score file score.sc containing the interface predicted aligned error (iPAE) and Cα RMSD of the peptide to the design model. Normalized iPAE (iPAE/32) cutoff values for filtering were chosen based on the iPAE distribution per target. 0.3 and 0.2 for MDM2 and MCL1, 0.13 and 0.4 for GABARAP and RbtA respectively. iPAE values over 0.4 were often nonoptimal interfaces or the peptide not interacting with the target in the designed conformations.

An example script for target–macrocycle complex structure prediction using AfCycDesign

```
pdb = 'diffused_binder_cyclic_1_mpnn1'
model = mk_afdesign_model('binder')
model.prep_inputs(f'{pdb}.pdb',
     binder_chain='B',
     target_chain='A',
     use_binder_template=False,
     use_multimer=True,
     use_initial_guess=True)
add_cyclic_offset(model, offset_type=2)
model.set_seq(mode='wildtype')
model.set_opt(num_recycles=1)
model.predict(models=[0,1], verbose=False)
model.save_pdb(f'diffused_binder_cyclic_1_mpnn1_prediction.pdb')
rmsd = model.aux['losses']['rmsd']
ipae = model.aux['all']['losses']['i_pae'][0]
with open(f'score.sc','a') as outfile:
     outfile.write(f'{pdb},{ipae},{rmsd}\n')
```

2.4. Filtering designs based on physics–based metrics of interface quality

After filtering design models based on the confidence metrics from DL–based structure prediction of design models (AfCycDesign and RF2), we used Rosetta (version 2021.12) to calculate the binding affinity (ddG), spatial aggregation propensity (SAP), and the contact molecular surface area (CMS) of the designed target macrocycle complexes. In the example below, target is defined as chain A and macrocycle is defined as chain B.

An example script for calculating physics–based interface metrics using Rosetta

```
<ROSETTASCRIPTS>
```

```
<SCOREFXNS>
	<ScoreFunction name="sfxn_cart" weights="beta_nov16_cart" >
		<Reweight scoretype="coordinate_constraint" weight="1" />
		<Reweight scoretype="atom_pair_constraint" weight="1" />
		<Reweight scoretype="dihedral_constraint" weight="1" />
		<Reweight scoretype="angle_constraint" weight="1" />
	</ScoreFunction>
</SCOREFXNS>
<RESIDUE_SELECTORS>
	<Chain name="chainA" chains="1"/>
	<Chain name="chainB" chains="2"/>
	<Neighborhood name="interface_chA" selector="chainB" distance="14.0" />
	<Neighborhood name="interface_chB" selector="chainA" distance="14.0" />
	<And name="AB_interface" selectors="interface_chA,interface_chB" />
	<Not name="Not_interface" selector="AB_interface" />
</RESIDUE_SELECTORS>
<TASKOPERATIONS>
	<ProteinInterfaceDesign name="pack_long"
		design_chain1="0"
		design_chain2="0"
		jump="1"
		interface_distance_cutoff="15"/>
	<OperateOnResidueSubset name="restrict_to_interface" selector="Not_interface">
		<PreventRepackingRLT/>
	</OperateOnResidueSubset>
</TASKOPERATIONS>
<MOVERS>
	<PeptideCyclizeMover name="pcm" residue_selector="chain%%chain%%"/>
	<TaskAwareMinMover name="minimize_interface"
		scorefxn="sfxn_cart"
		tolerance="0.01"
		cartesian="true"
		task_operations="restrict_to_interface"
		jump="0" />
	<TaskAwareMinMover name="min"
		scorefxn="sfxn"
		bb="0"
		chi="1"
		task_operations="pack_long" />
<FILTERS>
	<Ddg name="ddg"
		threshold="50"
		jump="1"
		repeats="5"
		repack="1"
		relax_mover="min"
		confidence="0"
		scorefxn="sfxn"
```

```
            extreme_value_removal="1" />
        <ContactMolecularSurface name="contact_molecular_surface"
            distance_weight="0.5"
            target_selector="chainA"
            binder_selector="chainB"
            confidence="0" />
<SIMPLE_METRICS>
        <SapScoreMetric name="sap_score" score_selector="chainB" />
</SIMPLE_METRICS>
<PROTOCOLS>
        <Add mover="pcm" />
        <Add mover="minimize_interface" />
        <Add mover="pcm" />
        <Add filter="ddg" />
        <Add metrics="sap_score" />
        <Add filter="contact_molecular_surface" />
    </PROTOCOLS>
</ROSETTASCRIPTS>
```

2.5. Structure–based clustering for binder design campaigns

To cluster structurally similar designed macrocycle binders, we first extracted them from their complexes. Then, each design was circularly permuted to generate all possible sequence representations of the macrocycle. At an arbitrarily selected example in the list of all designs to be clustered, Cα RMSD was calculated against all members of the list and their cyclic permutations. If Cα RMSD was less than 0.5 Å then that member and its permutations were removed from the list and clustered with the design currently looping through the list. This process was repeated until all members were placed into their respective clusters.

2.6. Prediction of RbtA Structure

The structure of RbtA was predicted using both RoseTTAFold2 and AlphaFold2. For both prediction methods, MSAs were originally generated following the procedure from the original RoseTTAFold publication[14], where hhblits was used to search both the Uniprot and BFD protein sequence databases at increasing e value cutoffs until at least 10000 sequences are found[12,25]. The resulting MSAs were then used as inputs to RoseTTAFold2 and AlphaFold2 (using CollabFold)[12,21,26]. In both cases, 5 models were generated (all 5 AlphaFold2 weights were used, and the single RoseTTAFold weights were used with 5 different random starting configurations); the model of the five with highest predicted accuracy (pLDDT) was chosen as the design target.

Both AlphaFold2 and RoseTTAFold2 yielded models with pLDDTs > 0.9, indicating very high confidence; the models were in very good agreement with one another.

3.3. Data Collection and Refinement Statistics
Supplementary Table 5: MCL–1–MCB_D2, RbtA–RBB_D10 and RbtA

	**MCL–1–MCB_D2** PDB ID: 9CDT	**RbtA–RBB_D10** PDB ID: 9CDU	**RbtA** PDB ID: 9CDV
Resolution range (Å)	33.43 – 2.1 (2.21 – 2.1)	34.56 – 2.61 (2.75 – 2.61)	34.09 – 2.00 (2.05 – 2.00)
Space group	$P\ 2_1\ 2_1\ 2$	$P\ 2_1\ 2_1\ 2$	$P\ 1\ 2_1\ 1$
Unit cell	52.48, 75.36, 43.37; 90, 90, 90	102.78, 169.84, 59.47; 90, 90, 90	57.16, 106.48, 71.31; 90.00, 107.04, 90.00
Unique reflections	10245 (1470)	32591 (4662)	55105 (4079)
Multiplicity	7.8 (7.7)	4.6 (4.8)	7.0 (7.2)
Completeness (%)	97.25 (99.93)	99.9 (100.0)	100.0 (100.0)
Mean I/sigma(I)	11.85 (2.30)	6.6 (1.0)	6.2 (1.1)
Wilson B–factor ($Å^2$)	41.81	58.62	26.72
R–merge	0.093 (0.860)	0.151 (1.478)	0.208 (1.616)
R–pim	0.035 (0.324)	0.088 (0.844)	0.092 (0.728)
$CC_{1/2}$	0.998 (0.850)	0.994 (0.409)	0.994 (0.559)
Reflections used in refinement	10241 (1470)	32432 (2262)	55054 (2928)
R–work	0.2164 (0.3240)	0.2123 (0.3198)	0.1954 (0.2942)
R–free	0.2719 (0.4208)	0.2587 (0.3860)	0.2344 (0.3416)
Number of non–hydrogen atoms	1350	6901	7217

macromolecules	1319	6774	6715
solvent	31	78	485
Protein residues	166	893	884
RMS(bonds)	0.003	0.002	0.002
RMS(angles)	0.490	0.490	0.510
Ramachandran favored (%)	96.30	93.39	94.04
Ramachandran allowed (%)	3.70	6.39	5.96
Ramachandran outliers (%)	0.00	0.23	0.00
Average B–factor (Å^2)	55	67	34
macromolecules	55	67	34
solvent	49	55	35

Supplementary Table 6: GABARAPL1–GAB_D8 and GABARAP–GAB_D23

	**GABARAPL1–GAB_D8** PDB ID: 9HGC	**GABARAP–GAB_D23** PDB ID: 9HGD
**Data collection**		
Resolution range (Å)	58.42–2.52 (2.59–2.52)	20.02–1.50 (1.54–1.50)
Space group	P 2_1 2_1 2_1	C 1 2 1
Unit cell: a, b, c (Å) α, β, γ (°)	51.29, 116.81, 116.83; 90.0, 90.0, 90.0	108.63, 43.79, 67.52; 90.0, 128.38, 90.0
Unique reflections	24336 (1803)	39046 (2844)
Multiplicity	13.3 (13.8)	6.85 (6.62)

Completeness (%)	99.4 (99.8)	97.0 (95.5)
Mean I/sigma(I)	10.01 (0.73)	13.34 (0.58)
Wilson B–factor (Å^2)	58.53	32.70
R–merge	0.229 (3.146)	0.062 (3.089)
R–pim	0.065 (0.871)	0.026 (1.280)
$CC_{1/2}$	0.996 (0.291)	0.999 (0.346)
**Refinement**		
Resolution range (Å)	58.42–2.52 (2.63–2.52)	18.42–1.50 (1.54–1.50)
No. reflections	24328 (2996)	38949 (2685)
R–work	0.2147 (0.3228)	0.1878 (0.4255)
R–free	0.2586 (0.3257)	0.2220 (0.4350)
Number of non–hydrogen atoms	4542	2714
polypeptides	4498	2519
ligands	22	35
solvent	22	160
Polypeptide residues	536	264
RMSD bonds (Å)	0.002	0.006
RMSD angles (°)	0.410	0.836
Ramachandran favored (%)	95.95	96.88

Ramachandran allowed (%)	3.85	3.12
Ramachandran outliers (%)	0.19	0.00
Avg. B–factor (Å^2)	58	38
polypeptides	58	38
ligands	68	53
solvent	50	44

Values for the highest–resolution shell are shown in parentheses.

4. Peptide Analytical Characterization Data

Supplementary Table 7: Purity of the chemically synthesized MCB designs

Design	Sequence	Purity (%)
MCB_D1	GAPEILKKMADMVGYE	87.12
MCB_D2	PPEIAWLADAVGLKDA	97.30
MCB_D3	GLETDDPVVKPLADAV	96.50
MCB_D4	APPEIKALADAVGLED	96.10
MCB_D7	SSTVAFLQEAVGMPVT	99.10
MCB_D8	EAVGLESDSELIKKLA	91.23
MCB_D12	SVDDPLIRKLAEAVGL	92.65
MCB_D13	SIDDPLLKKLAEAVGL	85.24
MCB_D15	ADALKRLTVPKNLEEI	97.10
MCB_D18	KPQPDIDPAFAEIVGF	96.00
MCB_D20	EDTLEGIARGLLTGKV	96.87
MCB_D21	YDTEEGIAEGLLTGKV	92.38
MCB_D22	ADTEEGIAKGLLTGKV	96.84
MCB_D26	GSPEIRWLMDAFGVDE	96.32

Supplementary Table 8: Purity of the chemically synthesized MDB designs

Design	Sequence	Purity (%)
MDB_D1	KKYNWMVDELVSMVGKPE	95.04
MDB_D2	EESEDEDFRVFERVLGI	97.00
MDB_D3	RELMEMVGEKYDQNFIL	88.06
MDB_D4	IESDDDFKVLTDVMGLD	95.14
MDB_D6	EDYQVLHDVLGLPLEFDD	91.19
MDB_D8	SRKAKNKFEELWNELIDP	95.09
MDB_D10	PGTPFAKEWSKMAGGAPL	90.64
MDB_D11	GLDLETDSEFAKEWQKMV	96.51

Supplementary Table 9: Purity of the chemically synthesized GAB designs

Design	Sequence	Purity (%)
GAB_D1	ETGEVENIDGVEIIYP	99.00
GAB_D8	GSEYEEDGWTVLEPD	97.15
GAB_D23	LEDGWVDIETGKE	99.46
GAB_D26	LDTGEVYKAPNGQEVIE	99.00
GAB_D27	IDIDTEEEVMPGV	95.87
GAB_D28	VMPGIIDIDTEEE	92.15

Supplementary Table 10: Purity of the chemically synthesized RBB designs

Design	Sequence	Purity (%)
RBB_D1	SSLPEYVRDVLGV	93.77
RBB_D2	GKPGDSPWEKIESVVFG	92.43
RBB_D3	PDPAIPENVQKVFKEHML	92.43
RBB_D4	PDPAFPENVQKVFKDKIE	98.04
RBB_D5	PDPAFPESIHKVFKDKII	93.22
RBB_D6	FSKYEPVDDSYPEGIRKV	92.11
RBB_D7	FEKYDPMDESYPENIRKV	94.63
RBB_D8	VEGNDKGSDIYEFIRDK	90.11
RBB_D10	KLFGPDPYLPENVQ	97.44
RBB_D14	SVAKEIAEWIGIPSKVPP	98.08
RBB_D15	SLAKEIADWIGIPSSVPP	94.52

MCB peptides Mass Spec and HPLC

Supplementary Figure 21: Analytical UPLC and mass spectrum of MCB_D1

Sequence: cyclo[APEILKKMADMVGYEG]

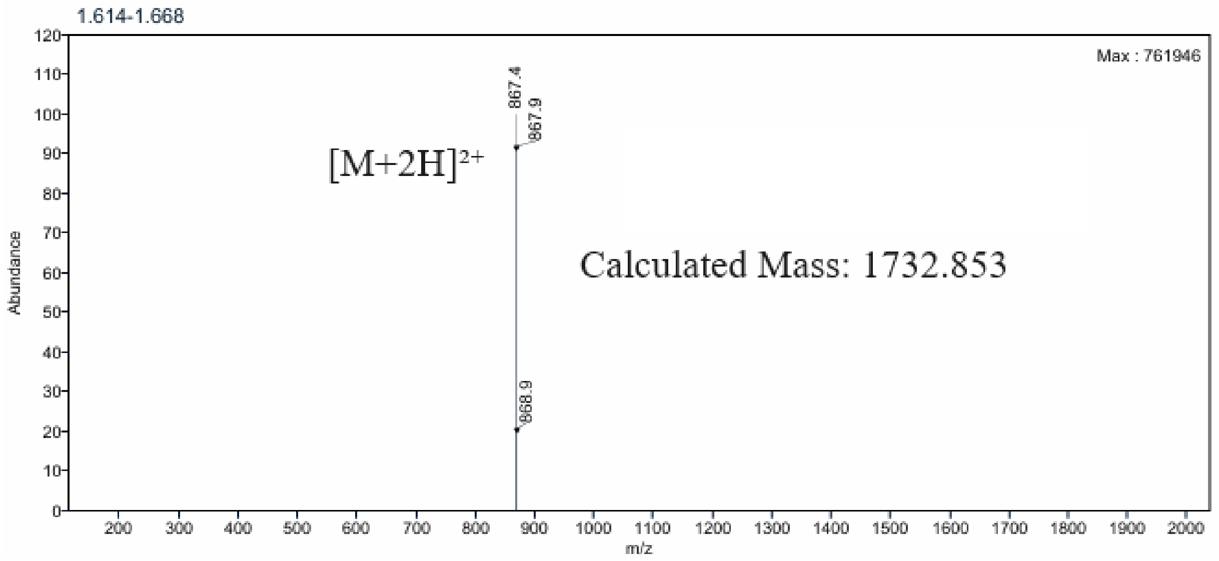

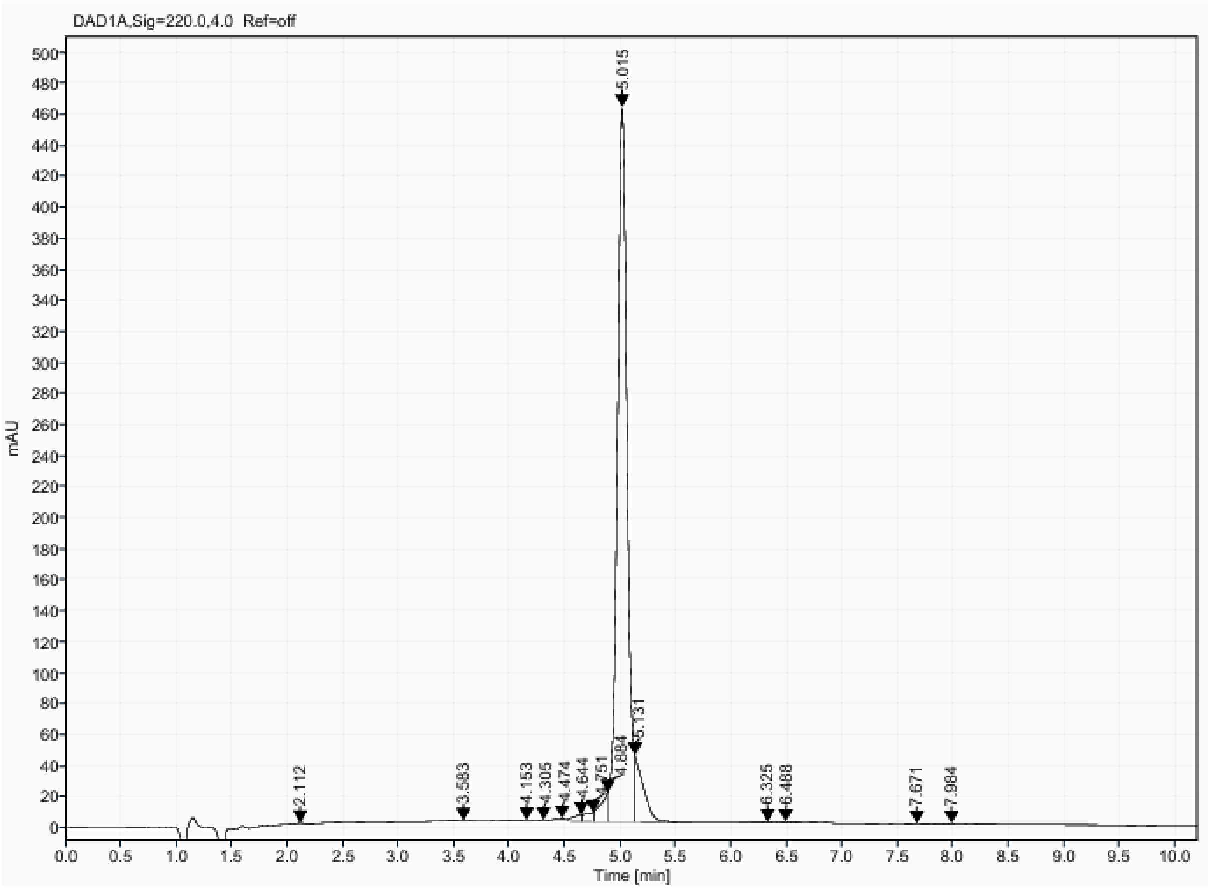

Supplementary Figure 22: Analytical UPLC and mass spectrum of MCB_D2
Sequence: cyclo[LKDAPPEIAWLADAVG]

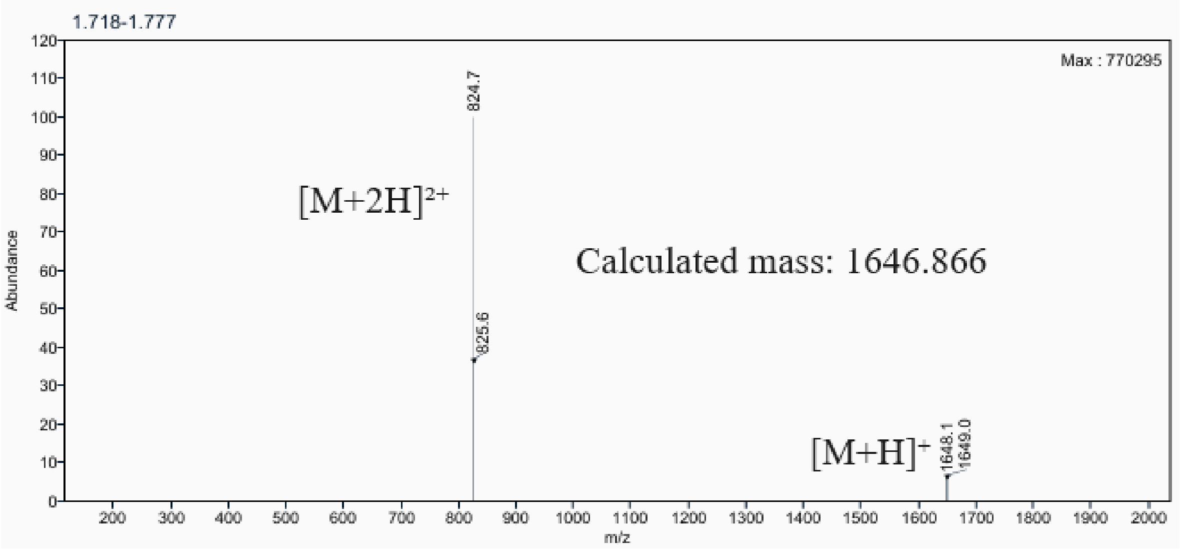

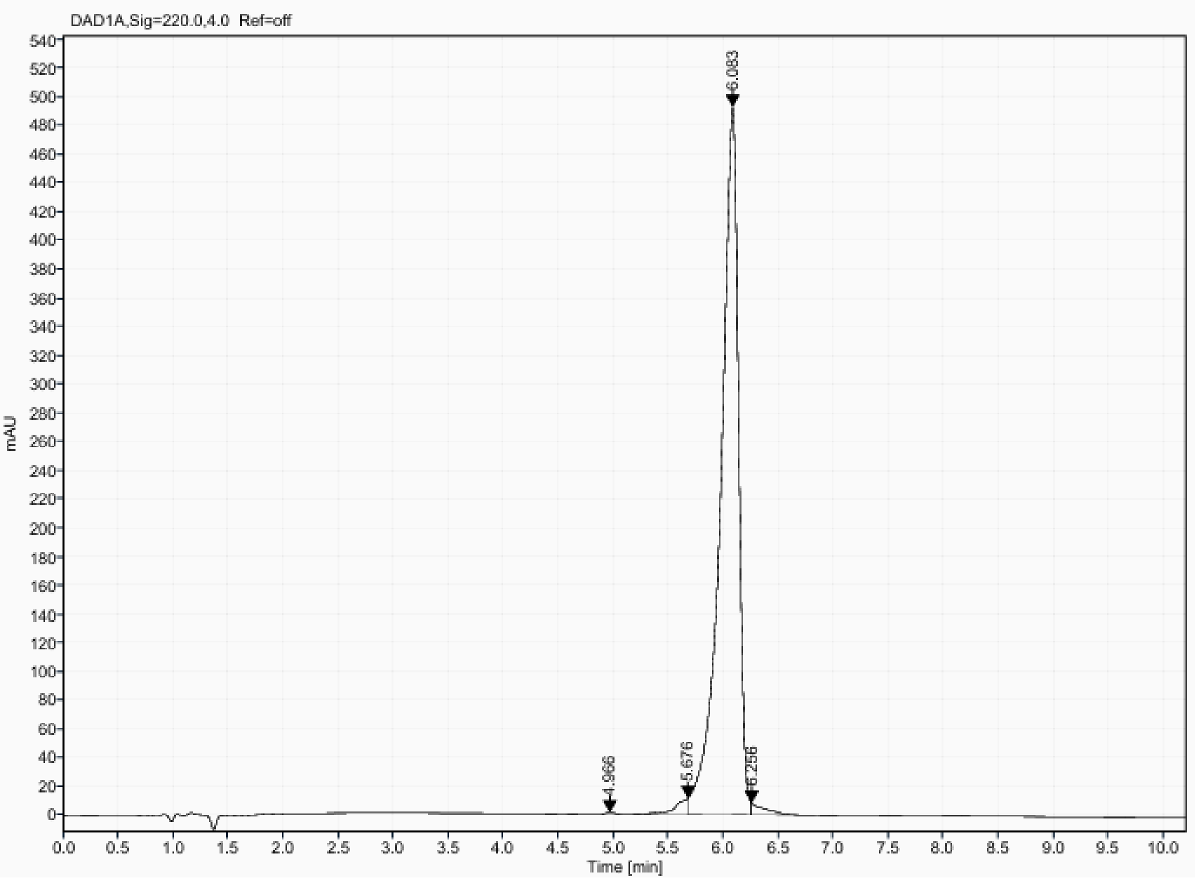

Supplementary Figure 23: Analytical UPLC and mass spectrum of MCB_D3
Sequence: cyclo[LETDDPVVKPLADAVG]

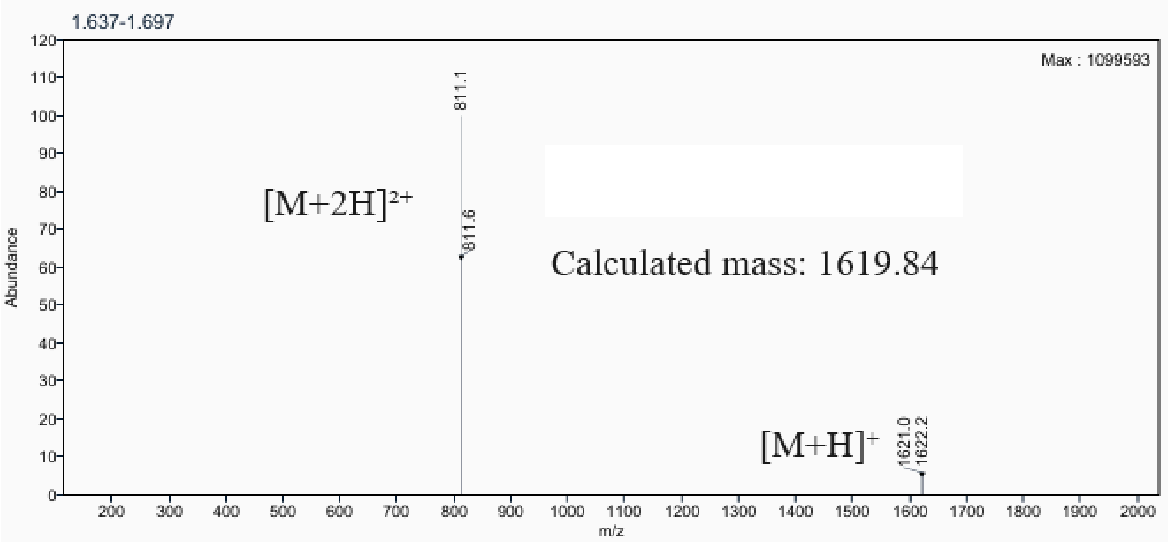

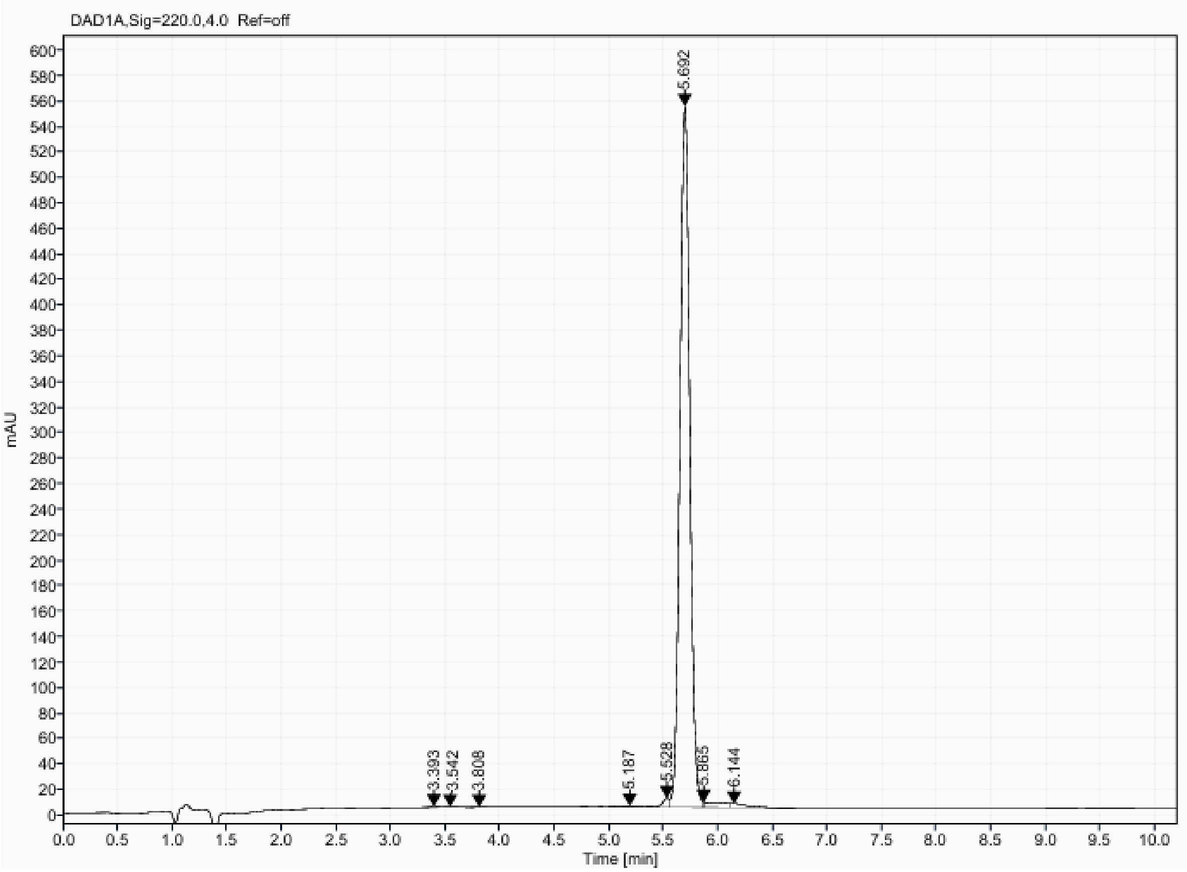

Supplementary Figure 24: Analytical UPLC and mass spectrum of MCB_D4
Sequence: cyclo[LEDAPPEIKALADAVG]

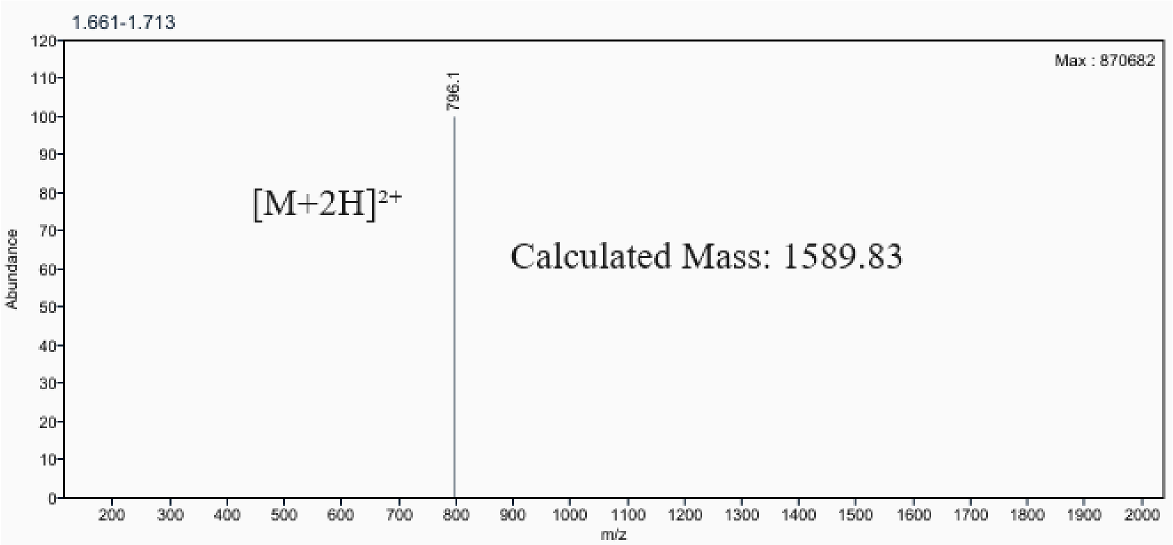

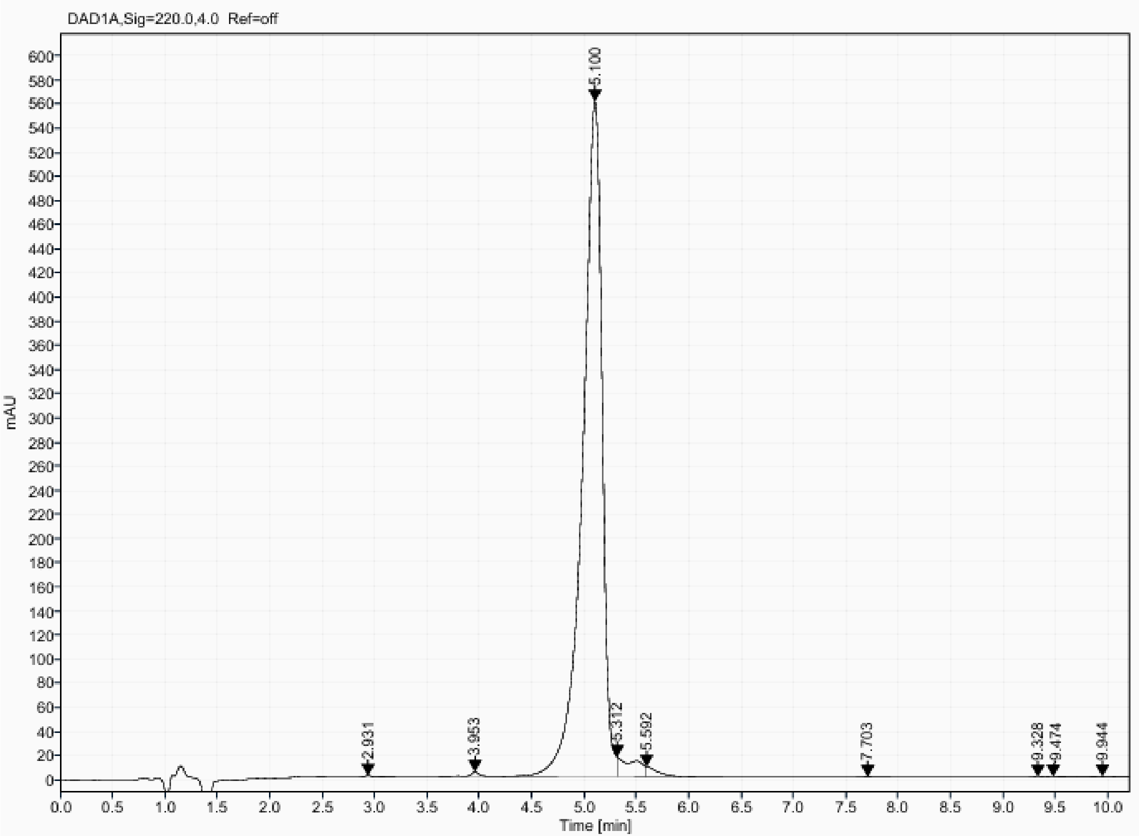

Supplementary Figure 25: Analytical UPLC and mass spectrum of MCB_D7
Sequence: cyclo[MPVTSSTVAFLQEAVG]

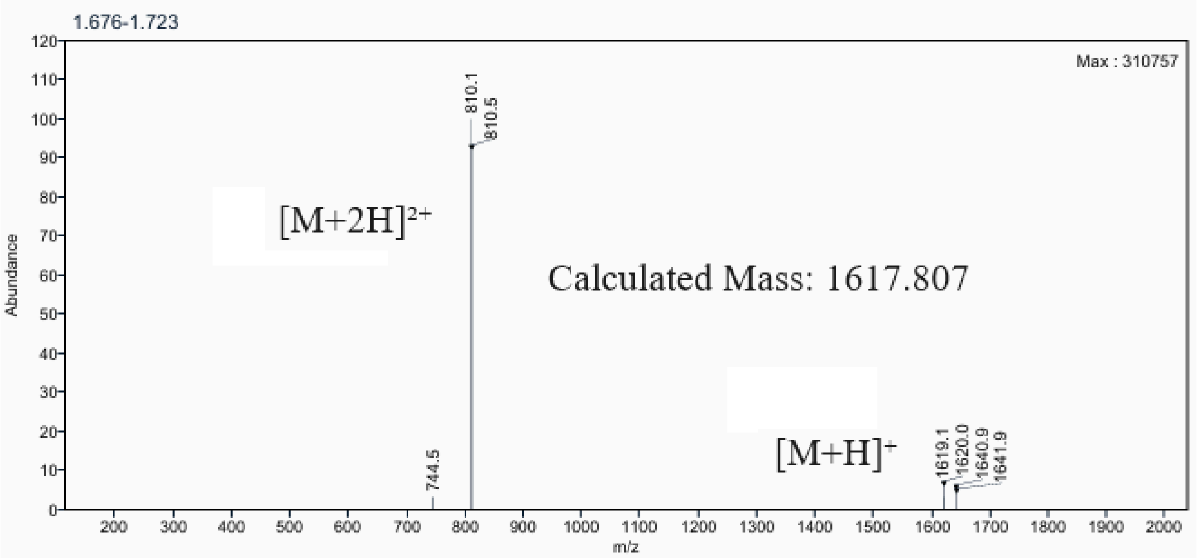

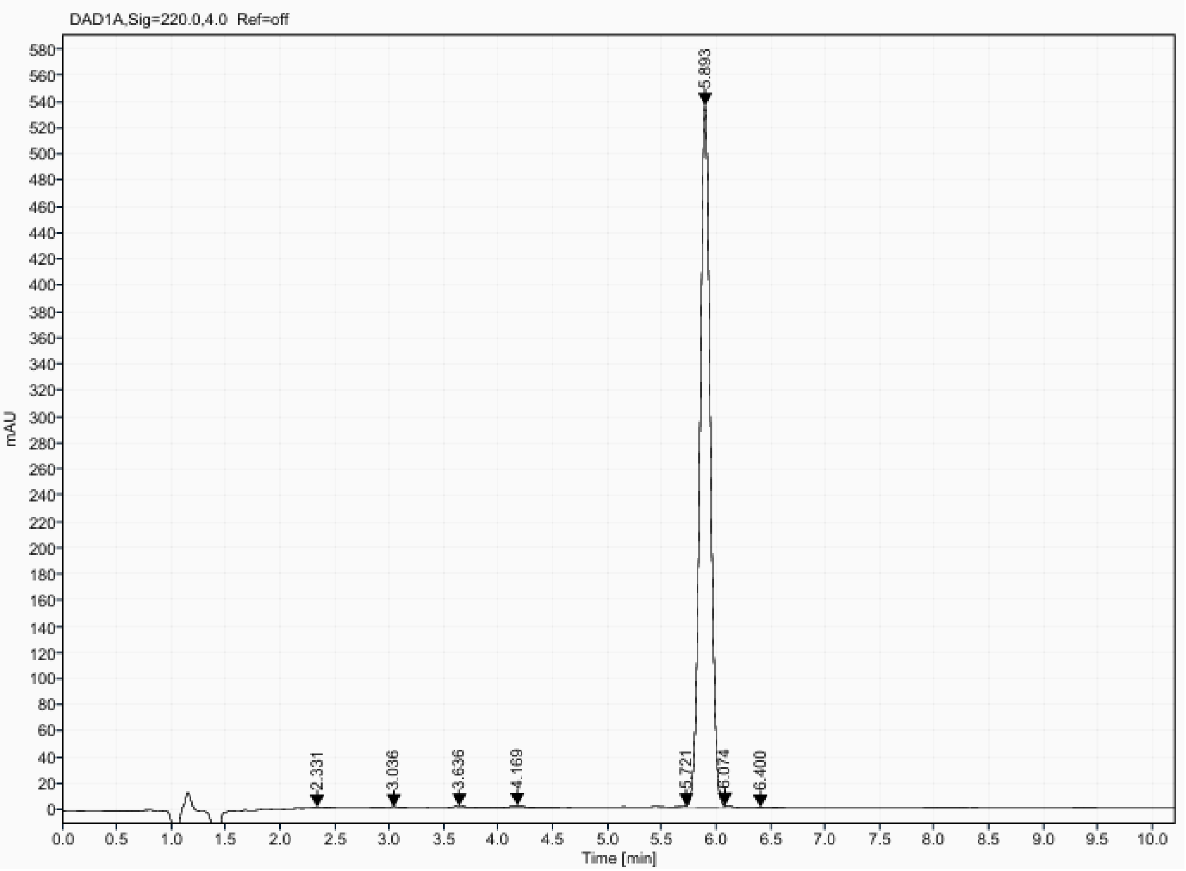

Supplementary Figure 26: Analytical UPLC and mass spectrum of MCB_D8
Sequence: cyclo[LESDSELIKKLAEAVG]

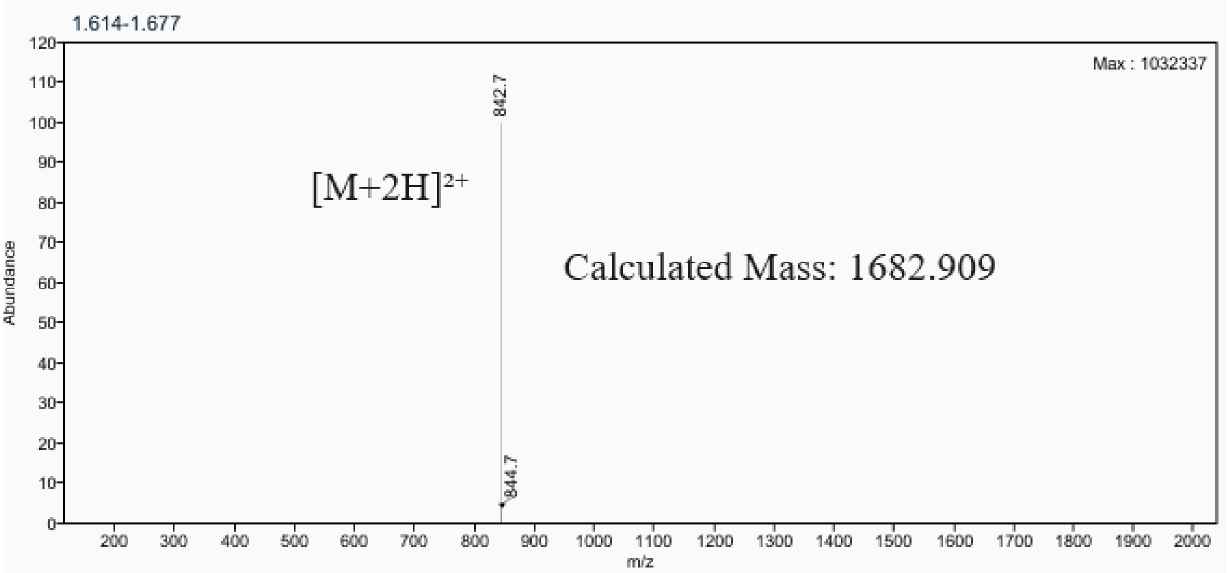

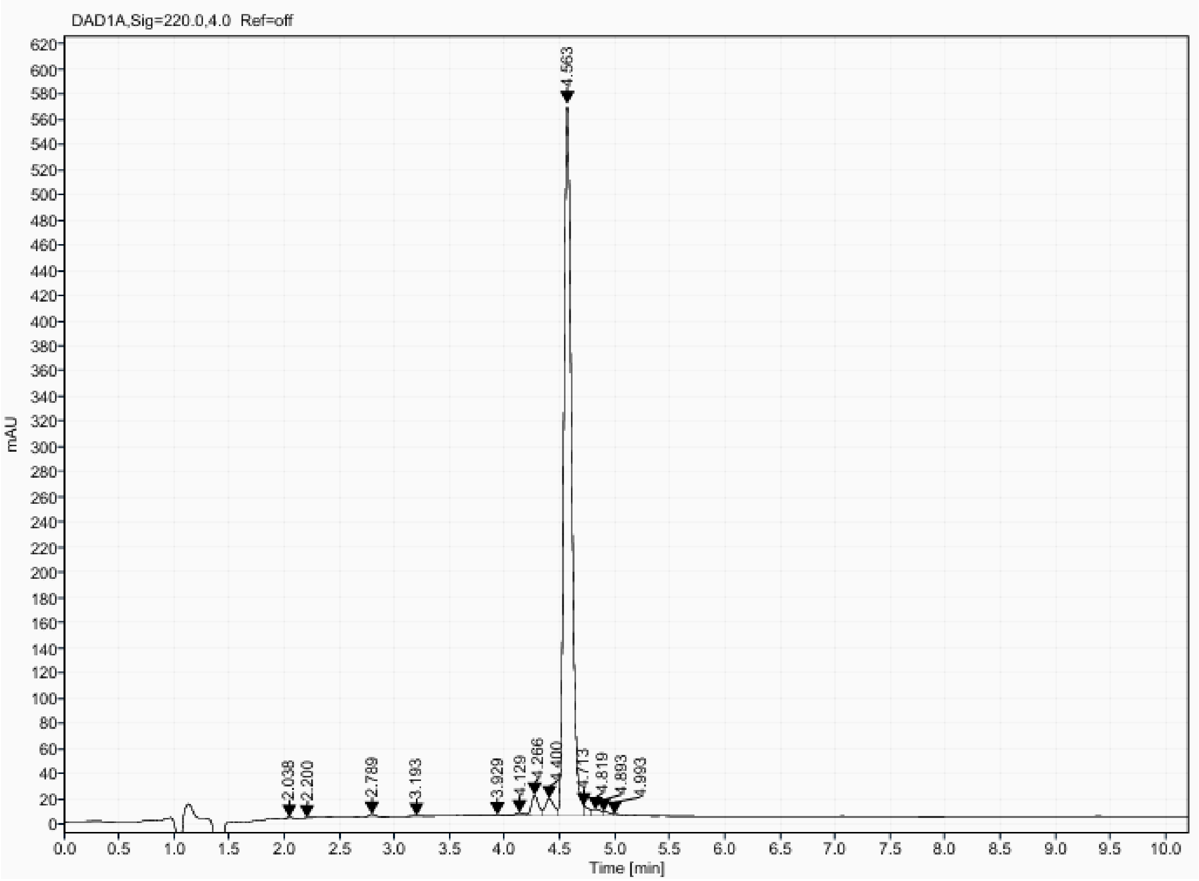

Supplementary Figure 27: Analytical UPLC and mass spectrum of MCB_D12
Sequence: cyclo[LSVDDPLIRKLAEAVG]

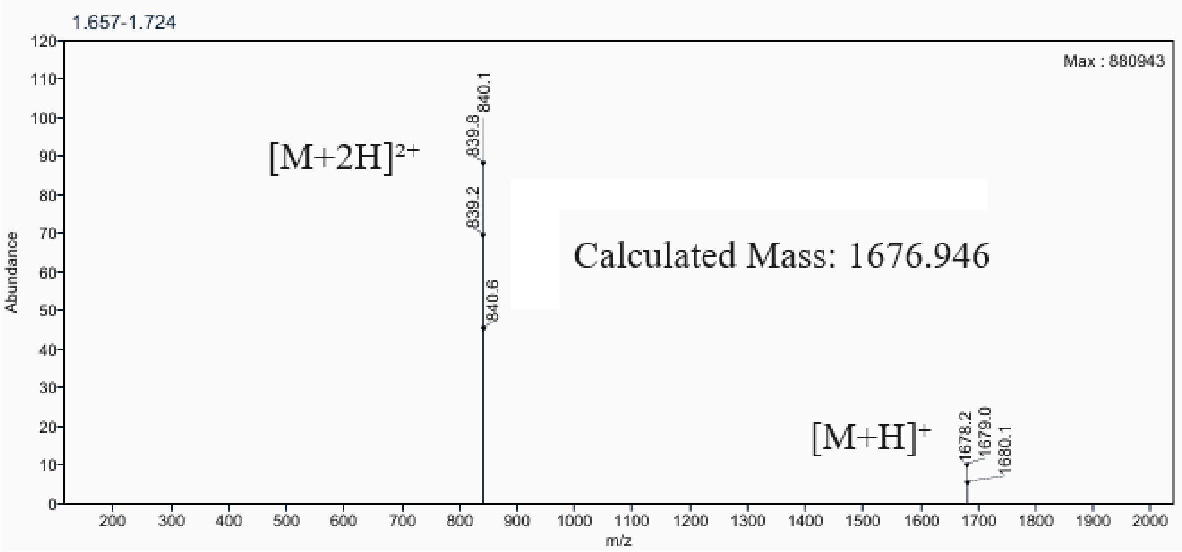

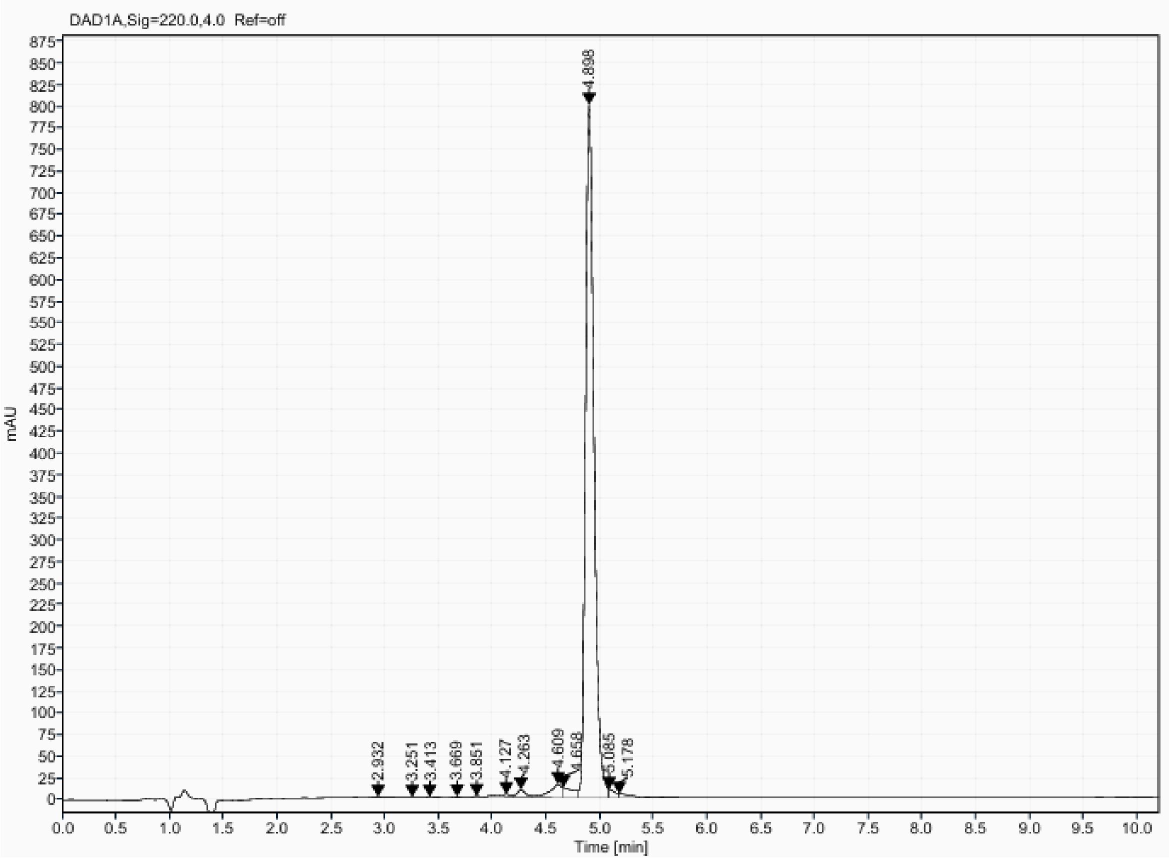

Supplementary Figure 28: Analytical UPLC and mass spectrum of MCB_D13
Sequence: cyclo[LSIDDPLLKKLAEAVG]

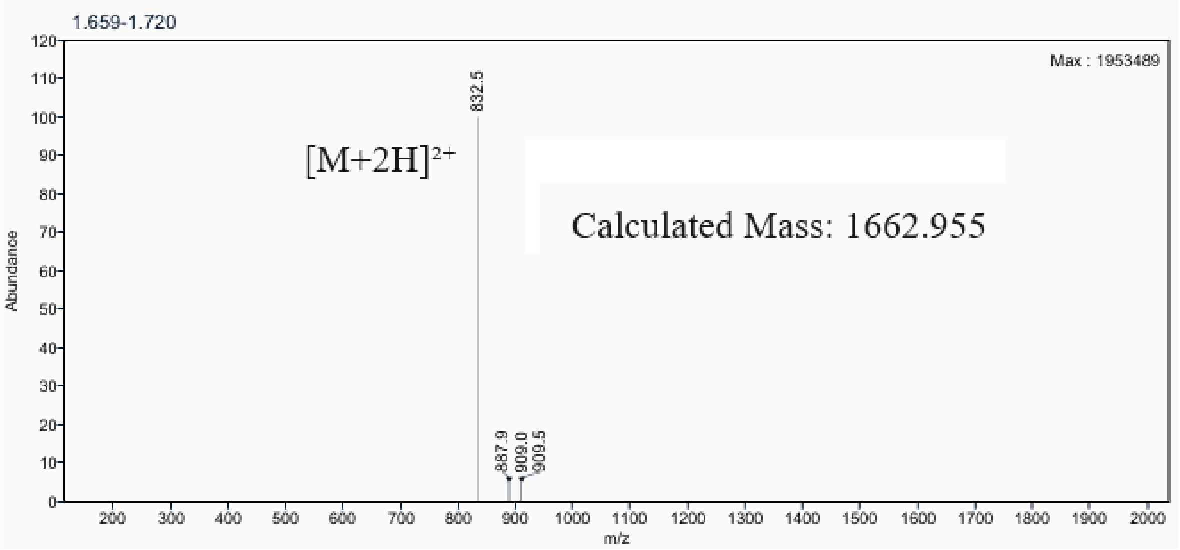

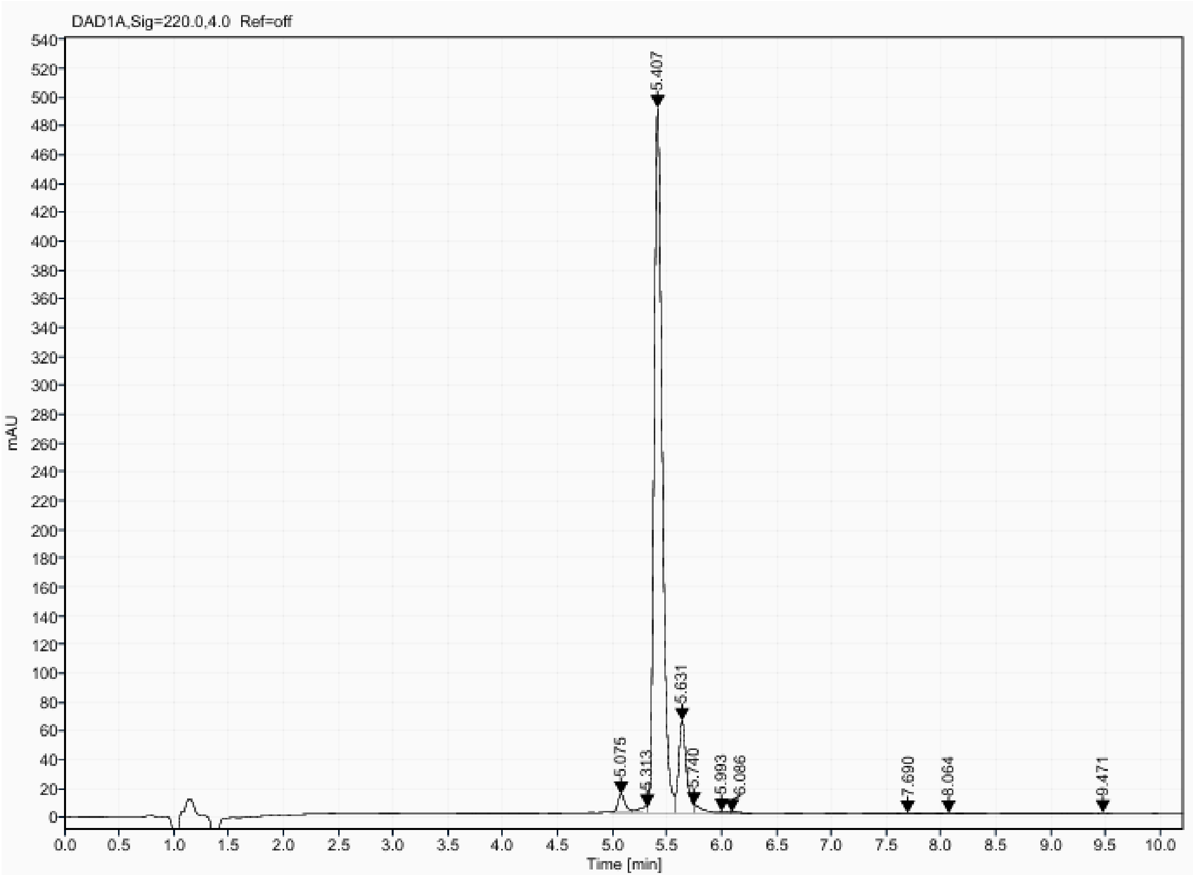

Supplementary Figure 29: Analytical UPLC and mass spectrum of MCB_D15
Sequence: cyclo[LKRLTVPKNLEEIADA]

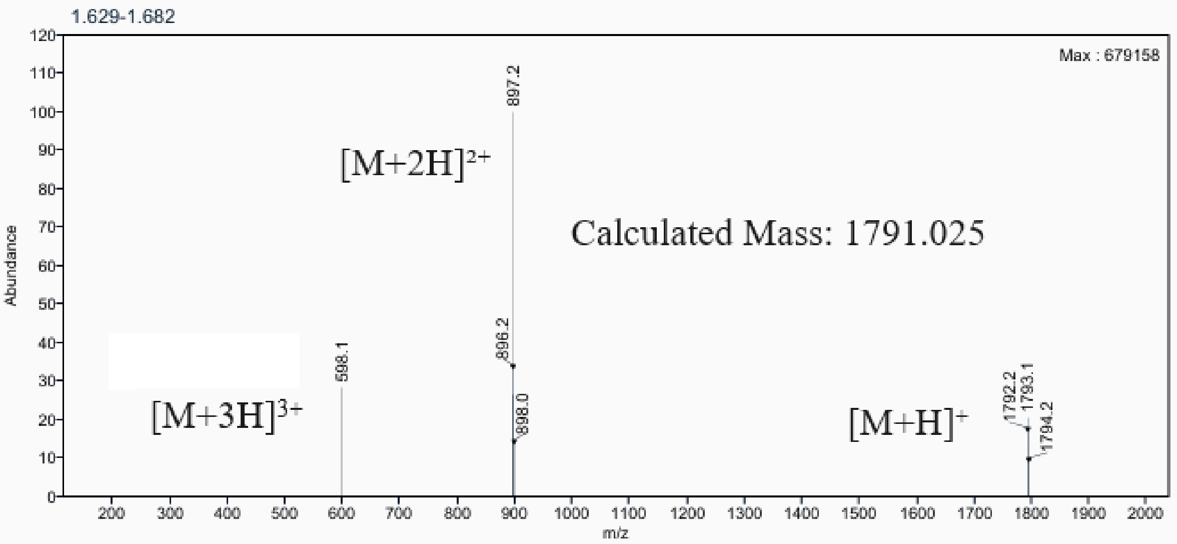

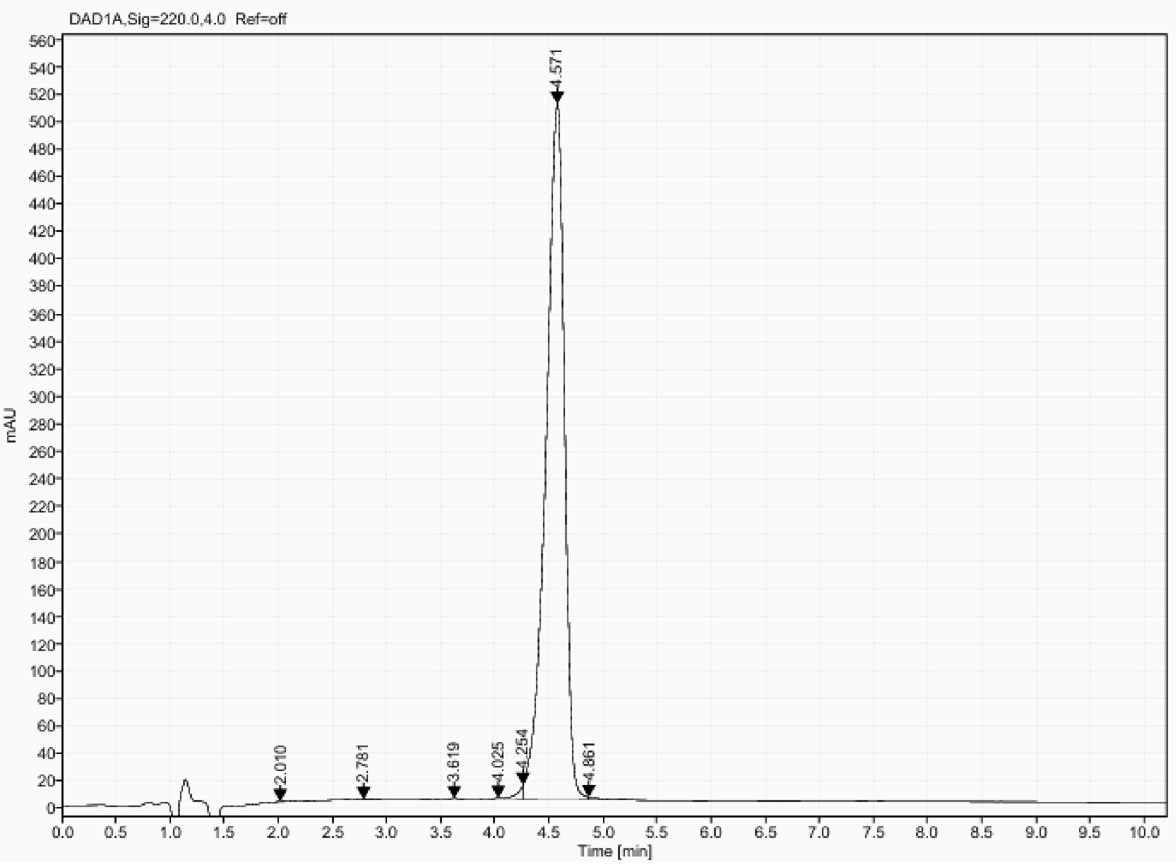

Supplementary Figure 30: Analytical UPLC and mass spectrum of MCB_D18
Sequence: cyclo[FKPQPDIDPAFAEIVG]

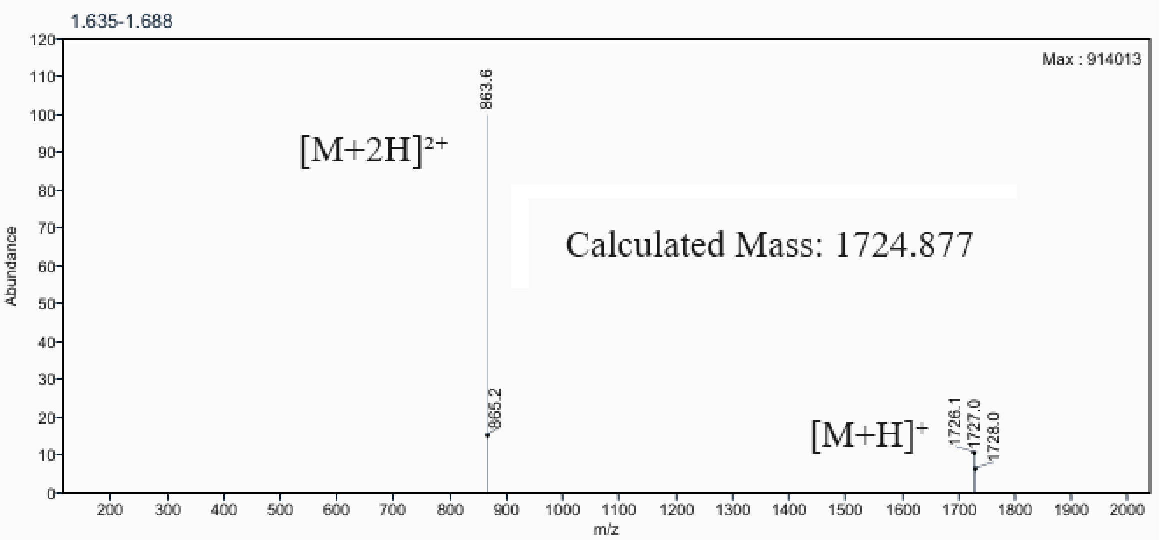

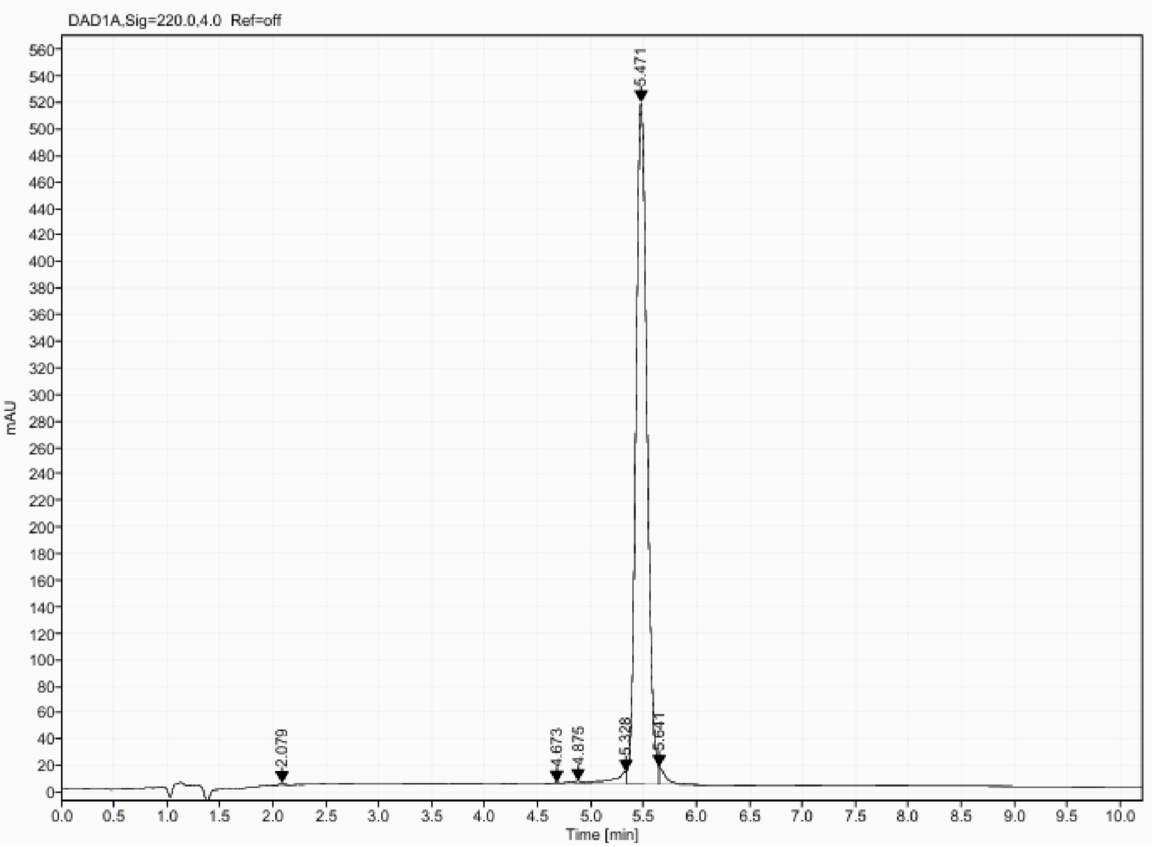

Supplementary Figure 31: Analytical UPLC and mass spectrum of MCB_D20
Sequence: cyclo[EDTLEGIARGLLTGKV]

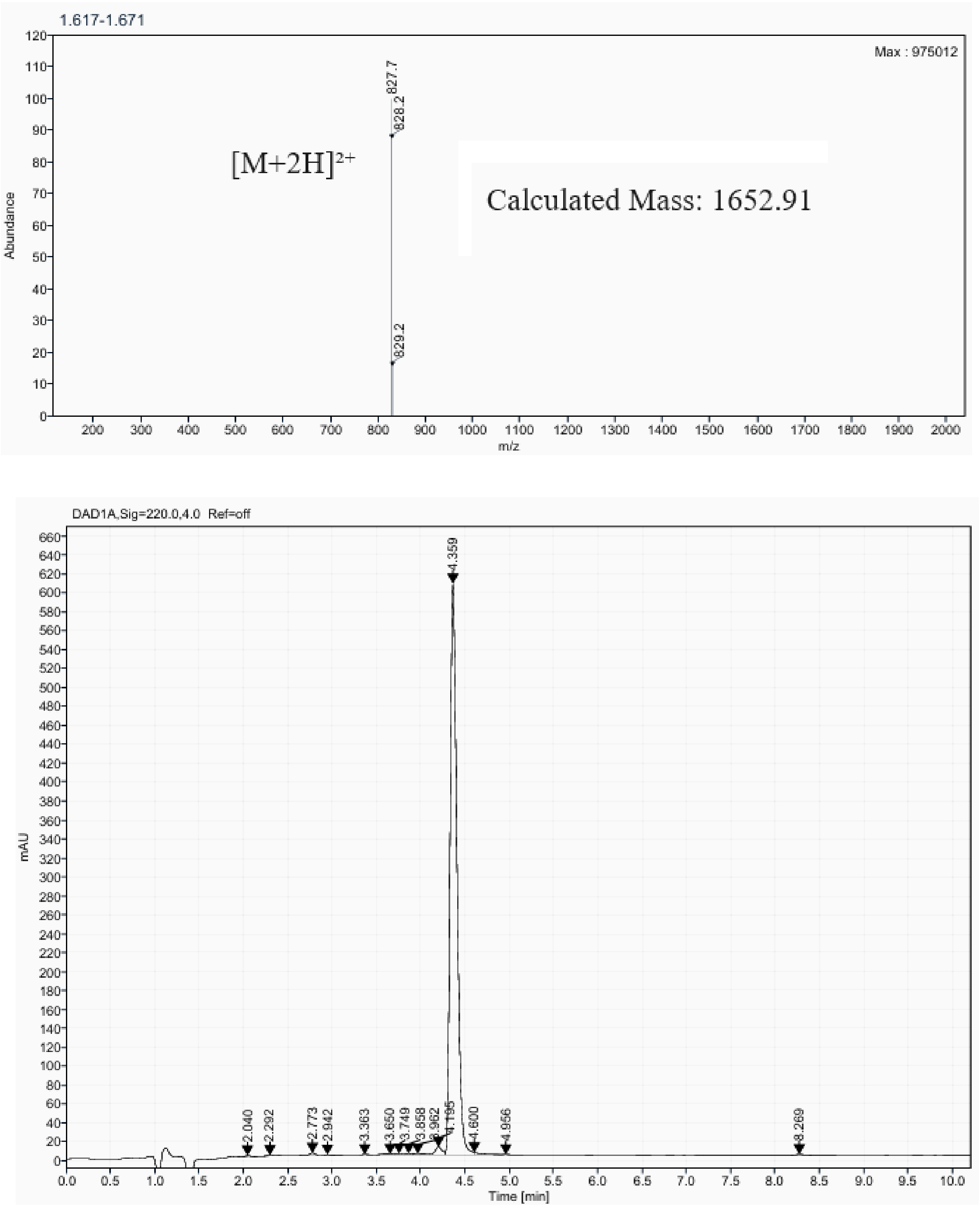

Supplementary Figure 32: Analytical UPLC and mass spectrum of MCB_D21
Sequence: cyclo[YDTEEGIAEGLLTGKV]

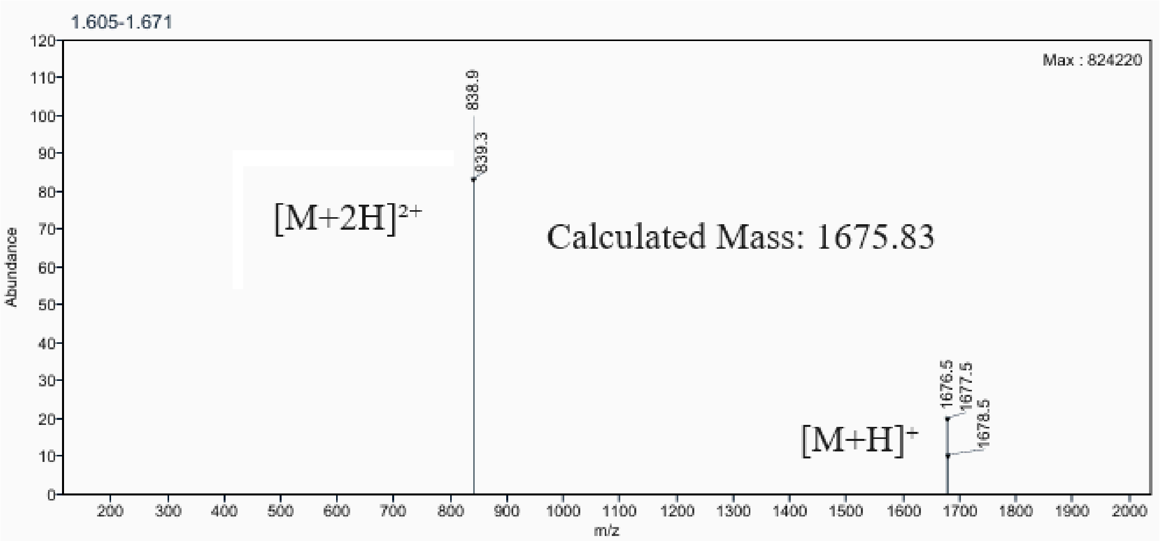

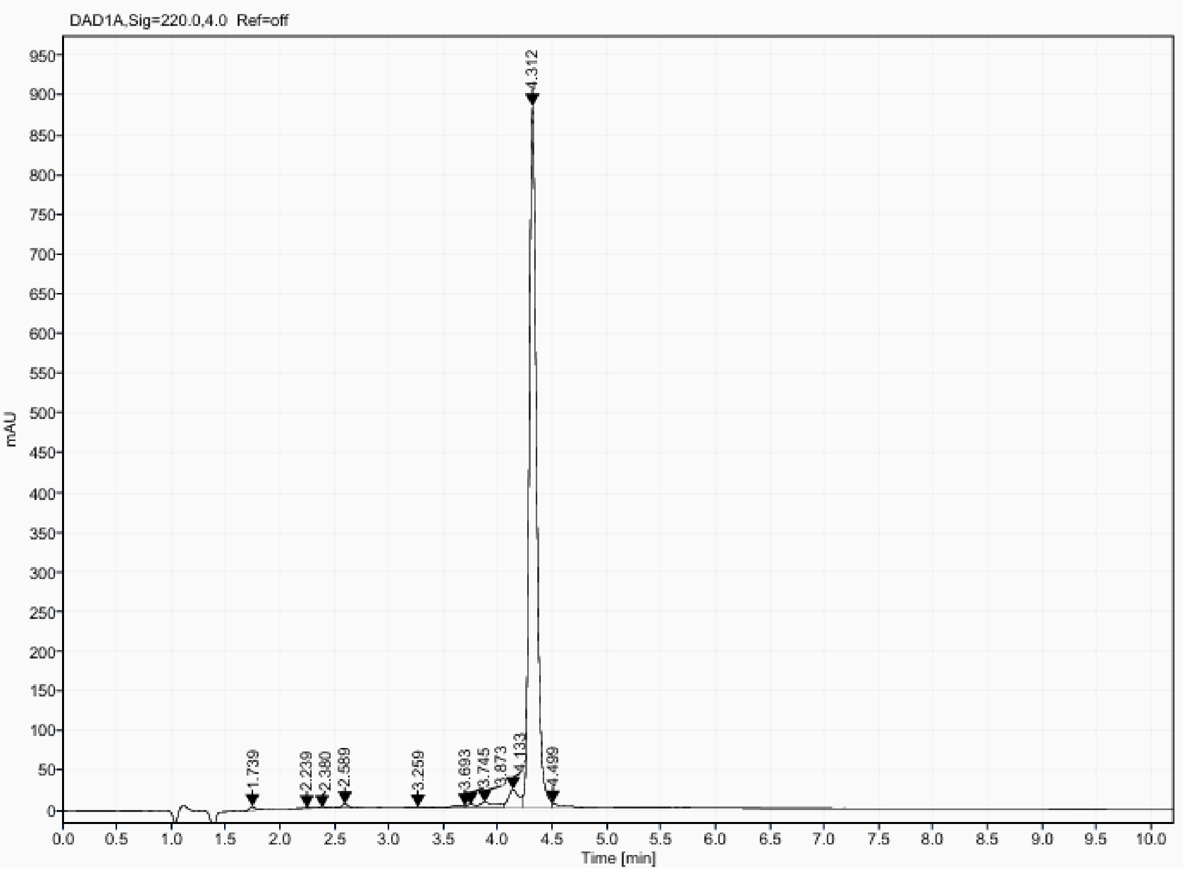

Supplementary Figure 33: Analytical UPLC and mass spectrum of MCB_D22
Sequence: cyclo[ADTEEGIAKGLLTGKV]

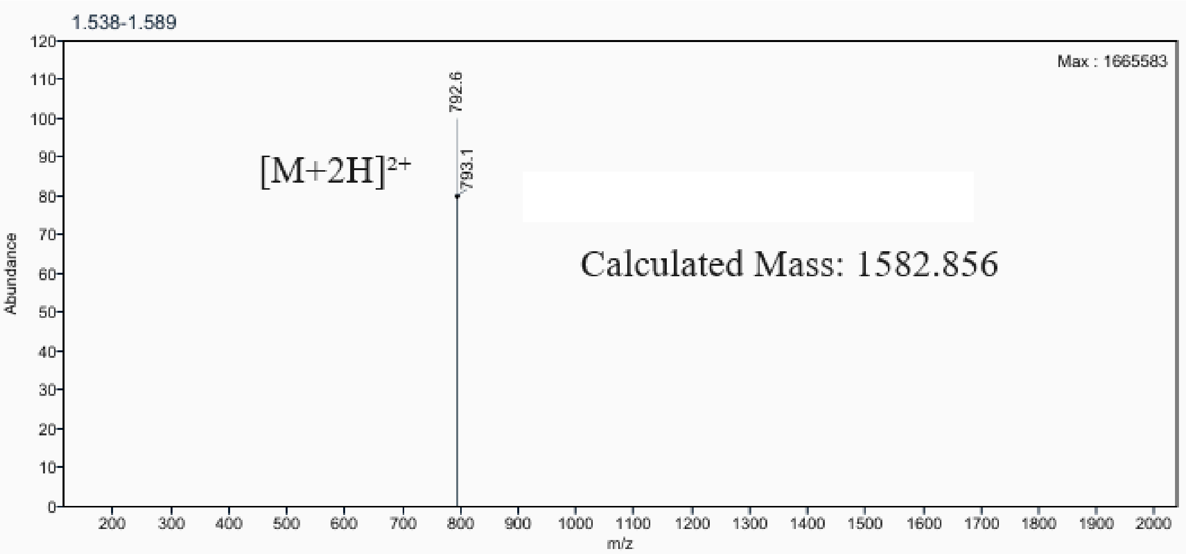

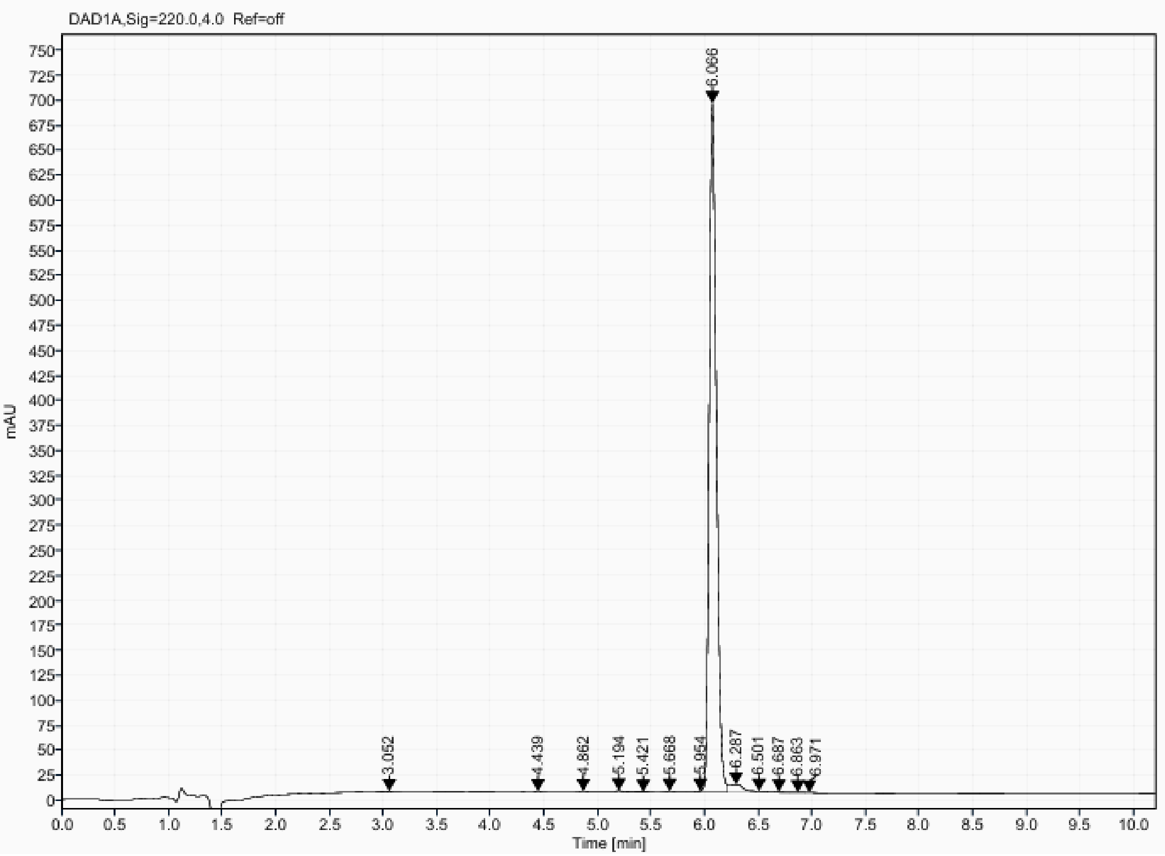

Supplementary Figure 34: Analytical UPLC and mass spectrum of MCB_D26
Sequence: cyclo[GSPEIRWLMDAFGVDE]

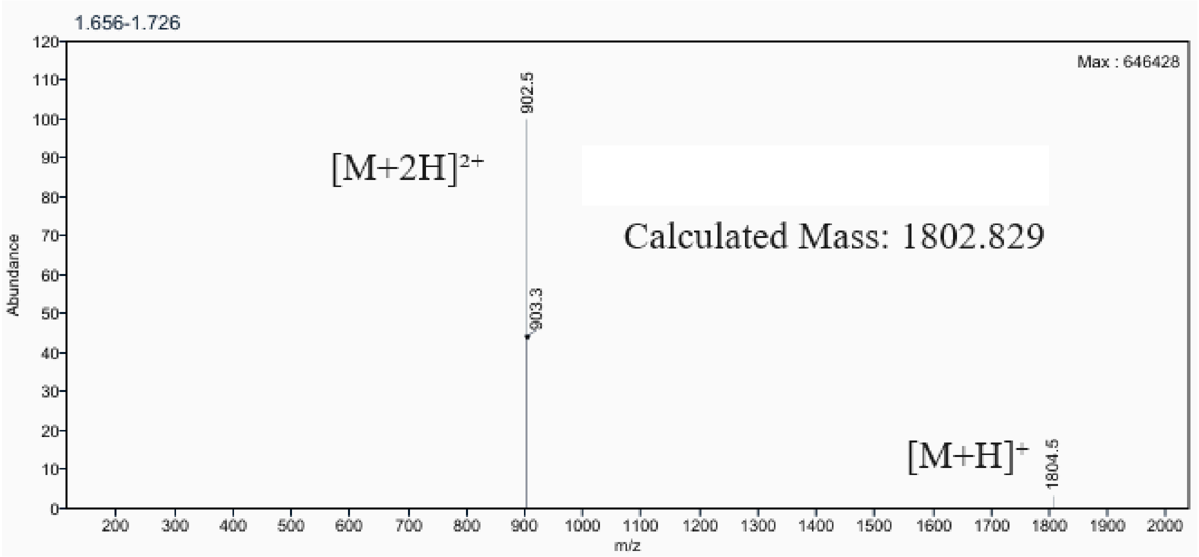

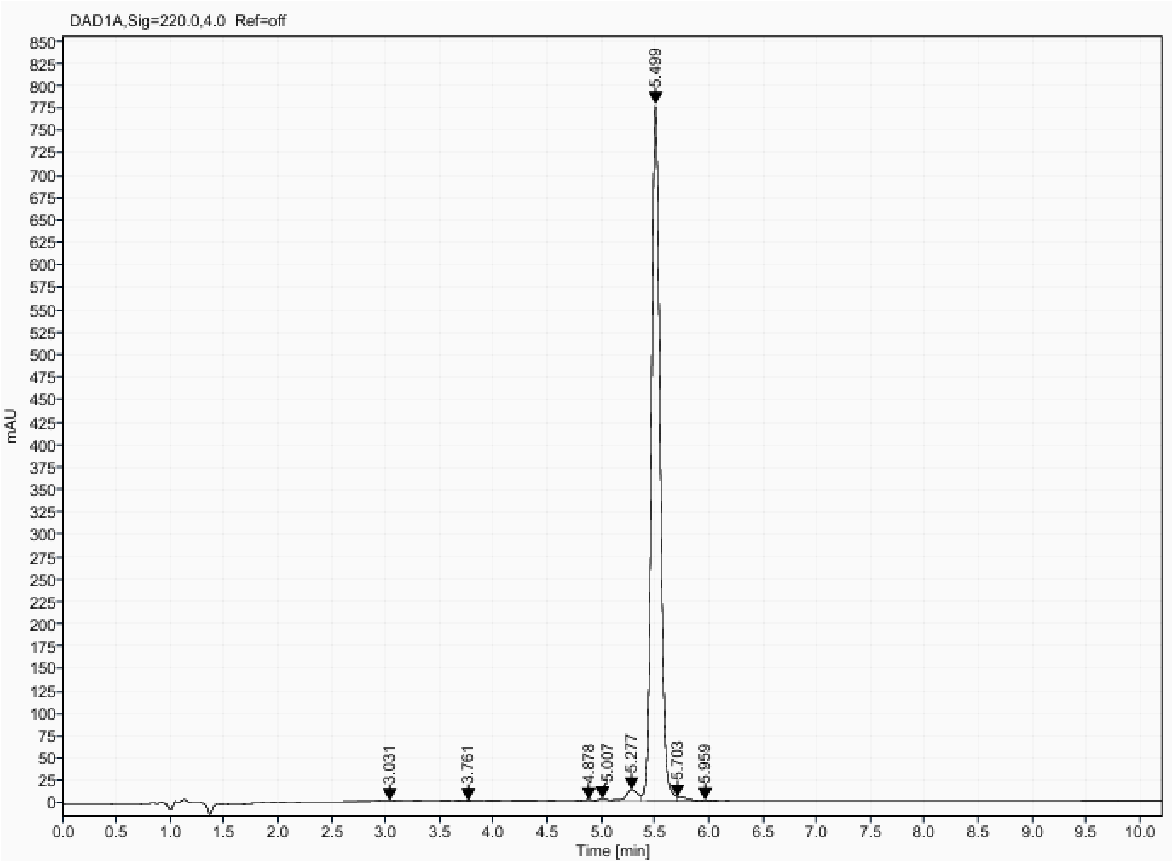

MDB peptides Mass Spec and HPLC

Supplementary Figure 35: Analytical UPLC and mass spectrum of MDB_D1
Sequence: cyclo[LVSMVGKPEKKYNWMVDE]

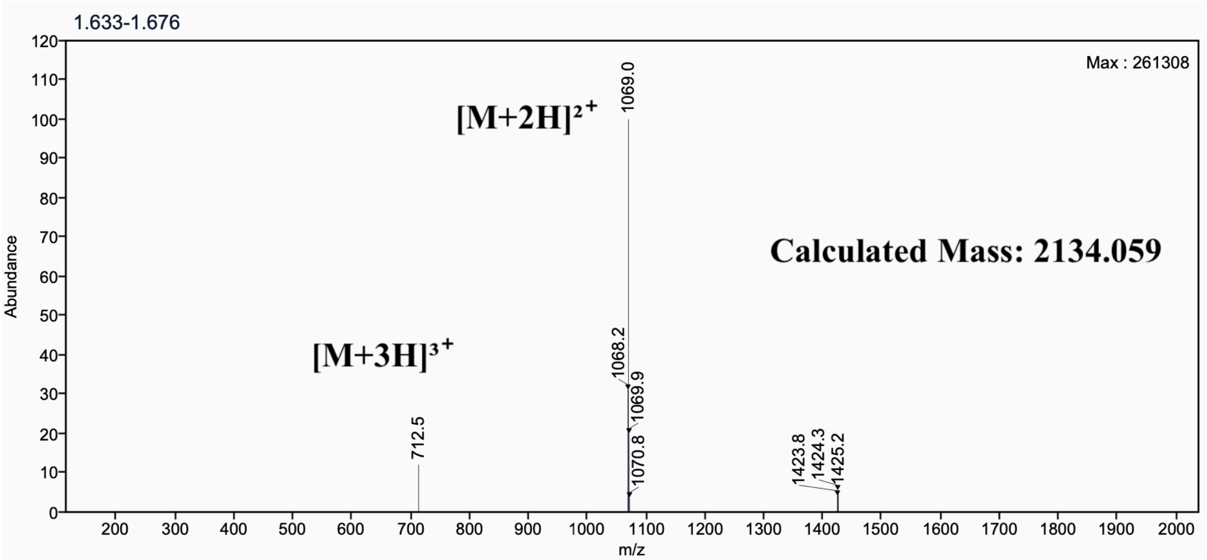

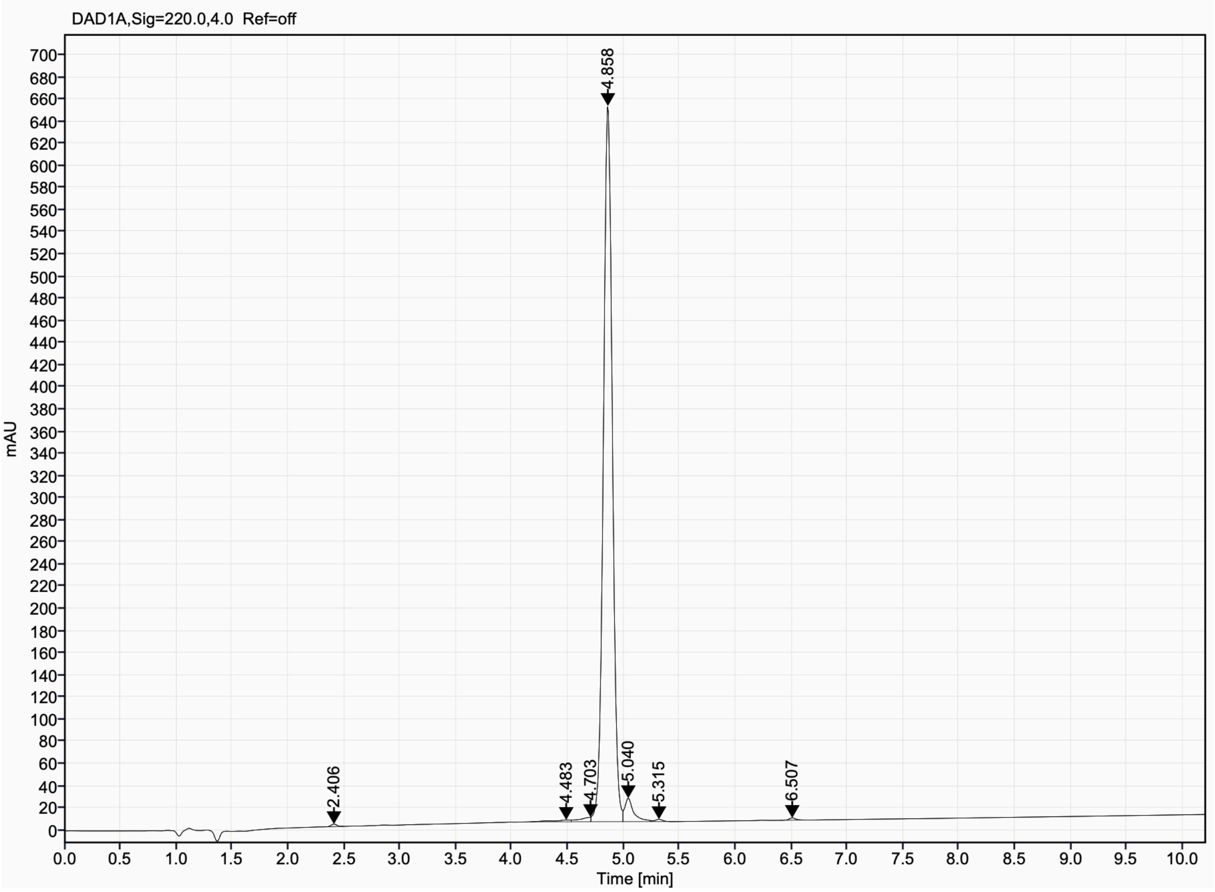

Supplementary Figure 36: Analytical UPLC and mass spectrum of MDB_D2
Sequence: cyclo[ERVLGIEESEDEDFRVF]

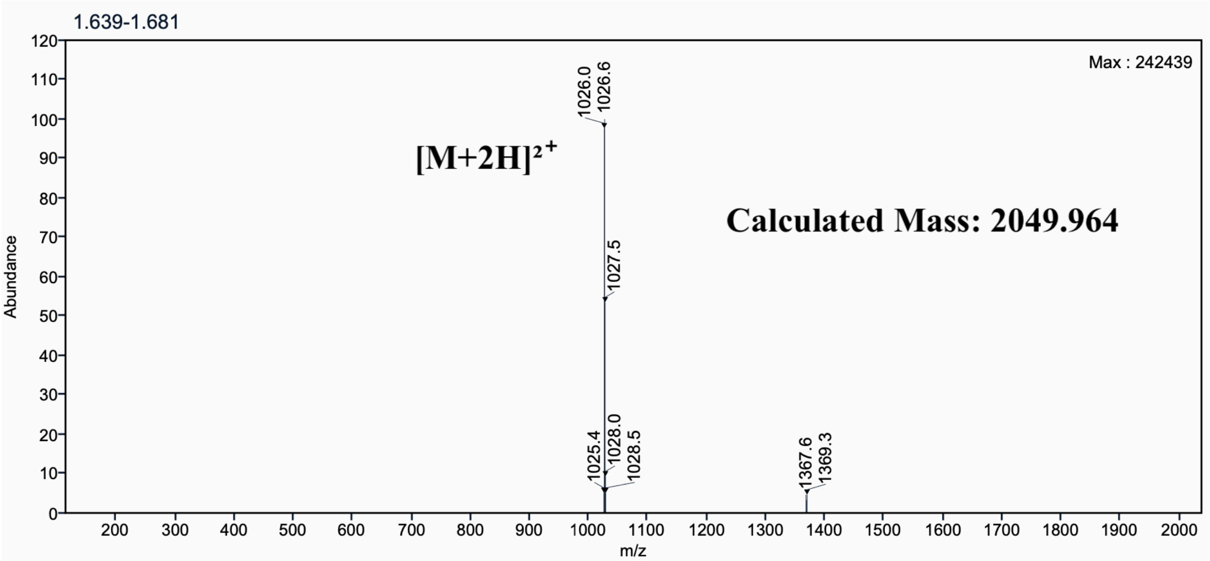

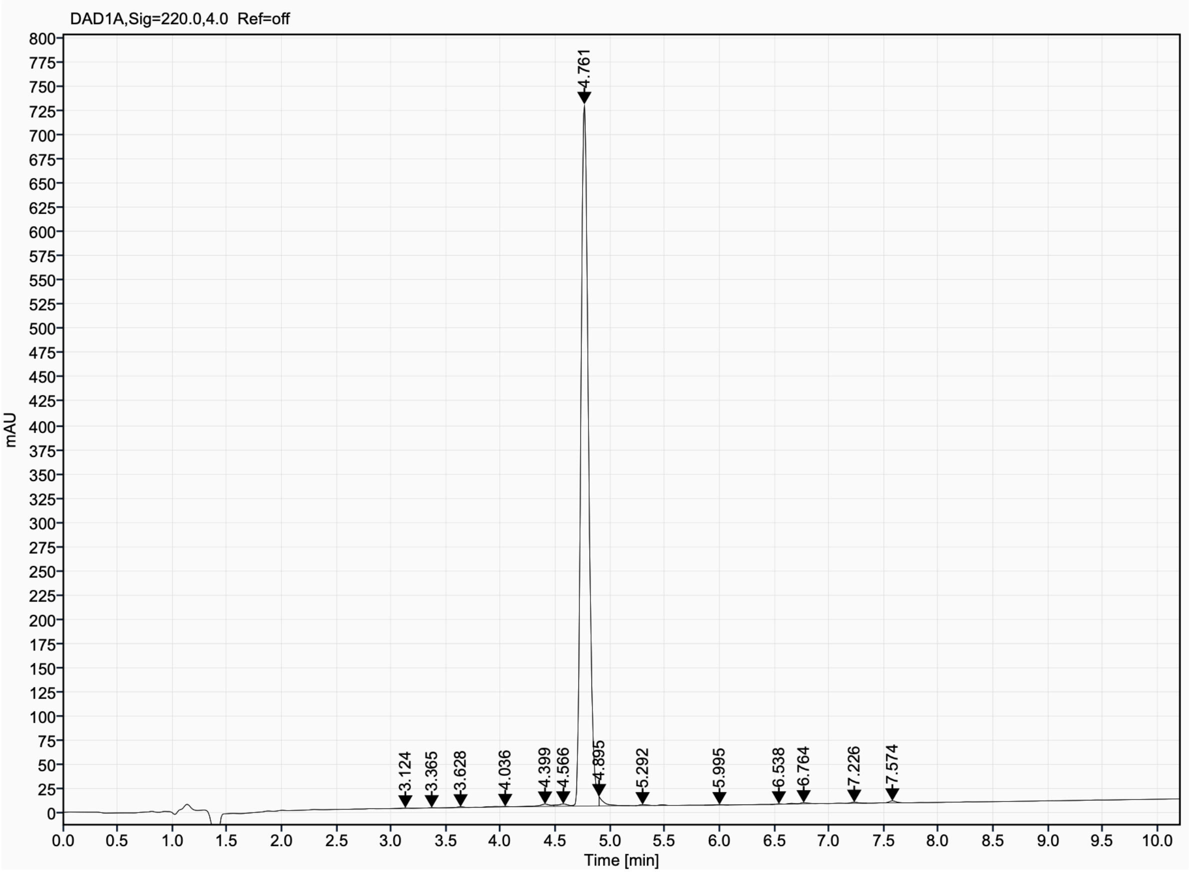

Supplementary Figure 37: Analytical UPLC and mass spectrum of MDB_D3
Sequence: cyclo[LMEMVGEKYDQNFILRE]

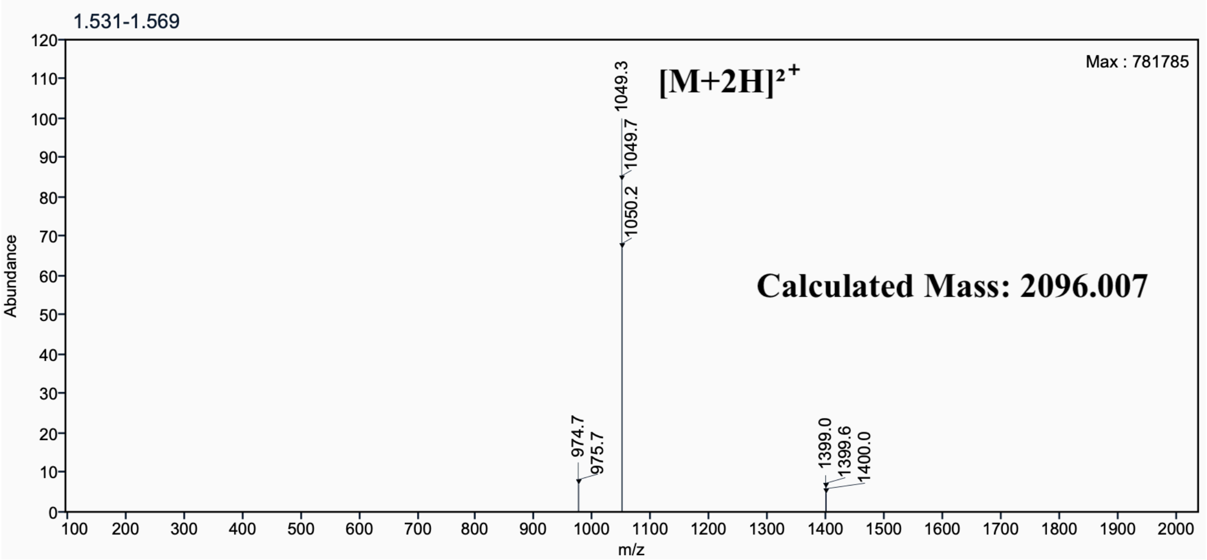

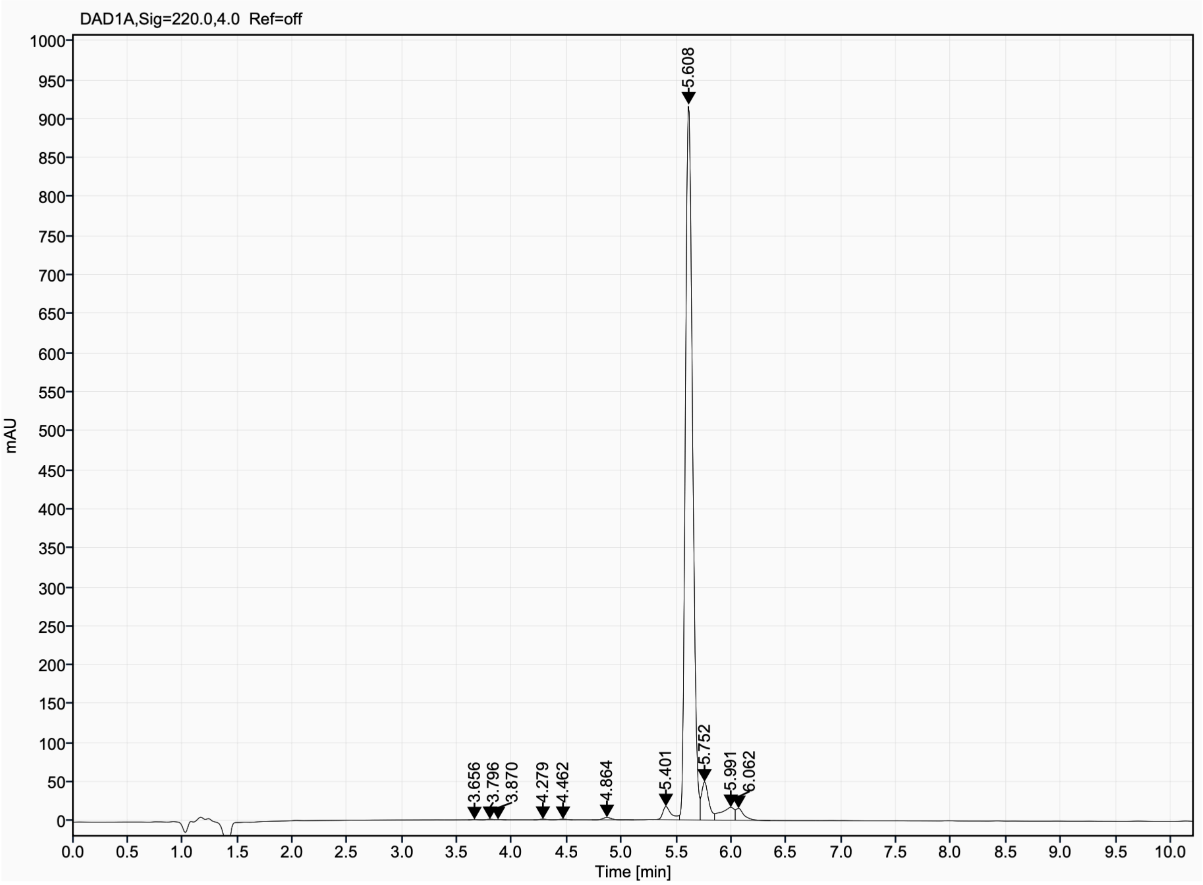

Supplementary Figure 38: Analytical UPLC and mass spectrum of MDB_D4
Sequence: cyclo[LTDVMGLDIESDDDFKV]

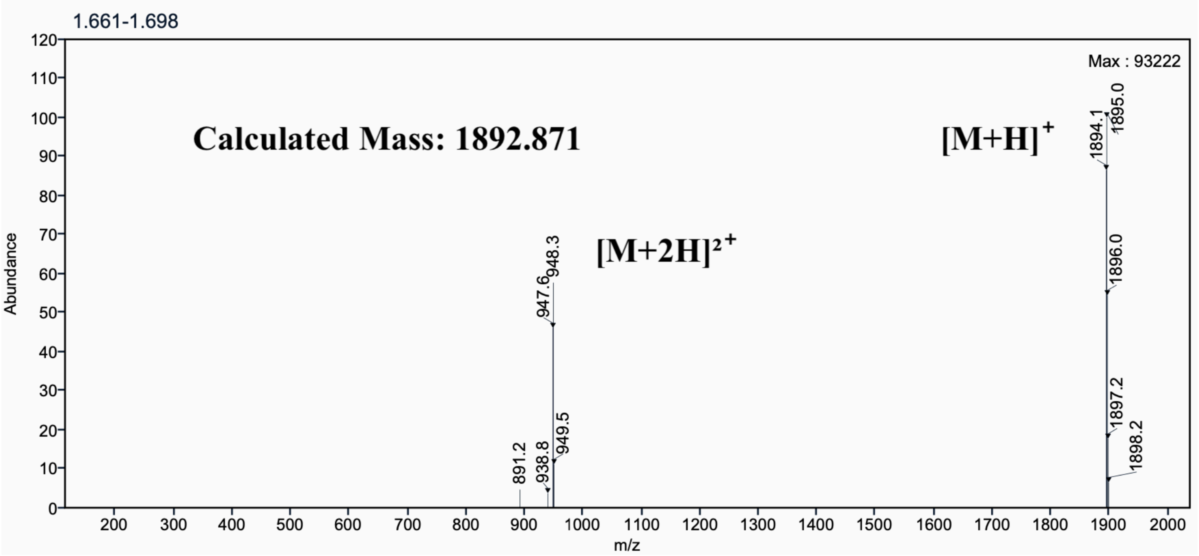

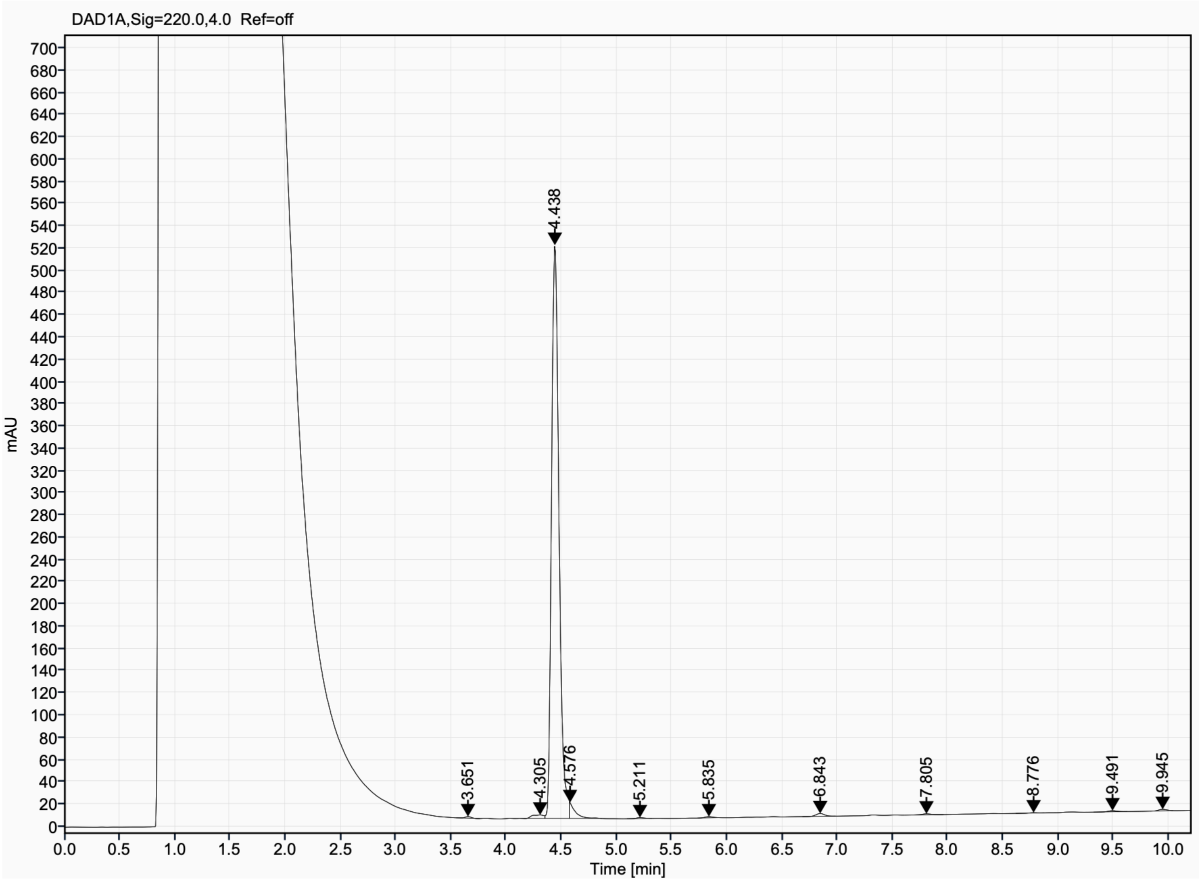

Supplementary Figure 39: Analytical UPLC and mass spectrum of MDB_D6
Sequence: cyclo[LHDVLGLPLEFDDEDYQV]

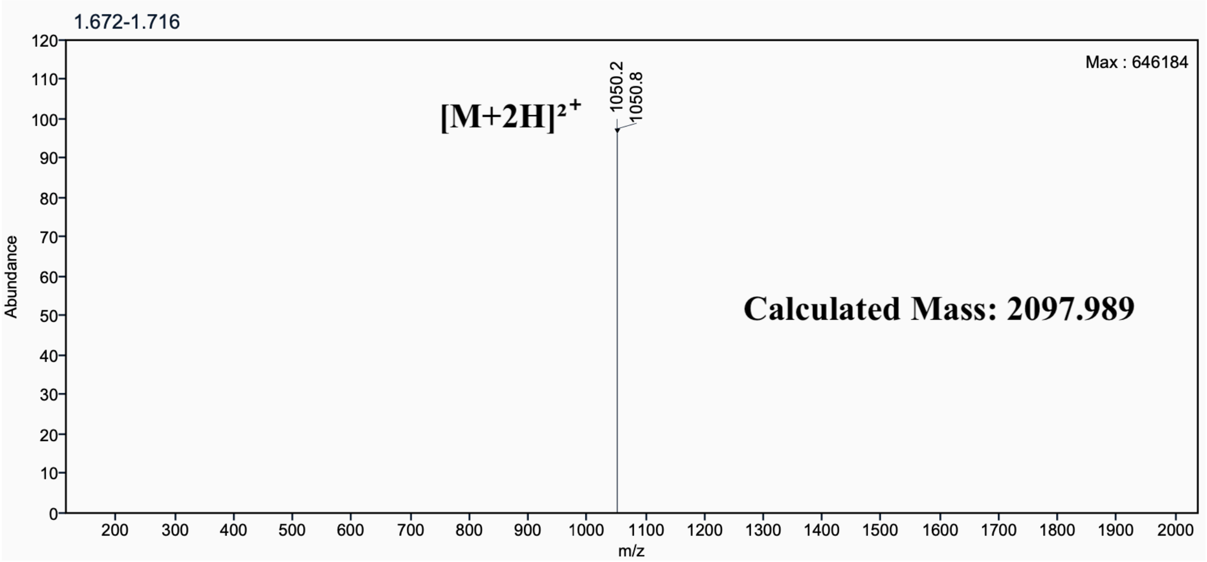

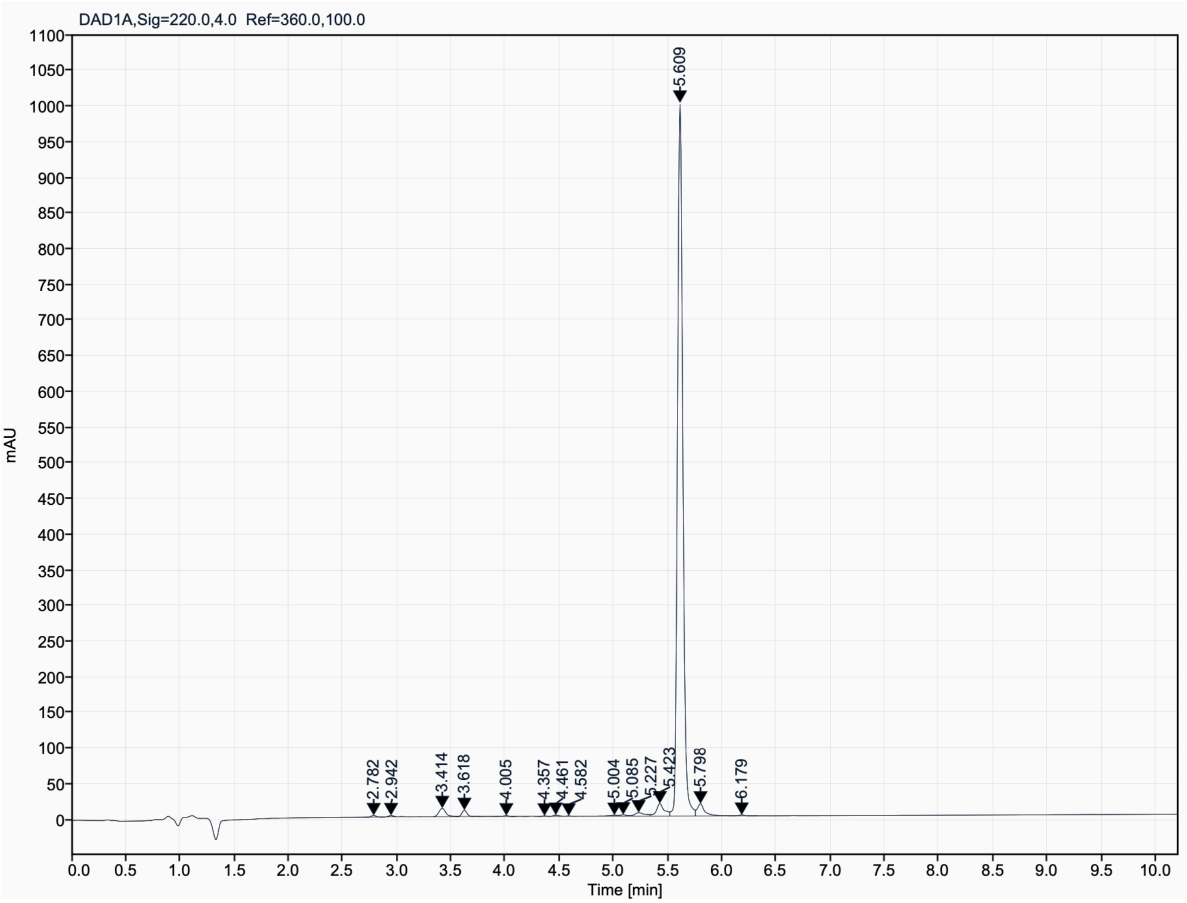

Supplementary Figure 40: Analytical UPLC and mass spectrum of MDB_D8
Sequence: cyclo[AKNKFEELWNELIDPSRK]

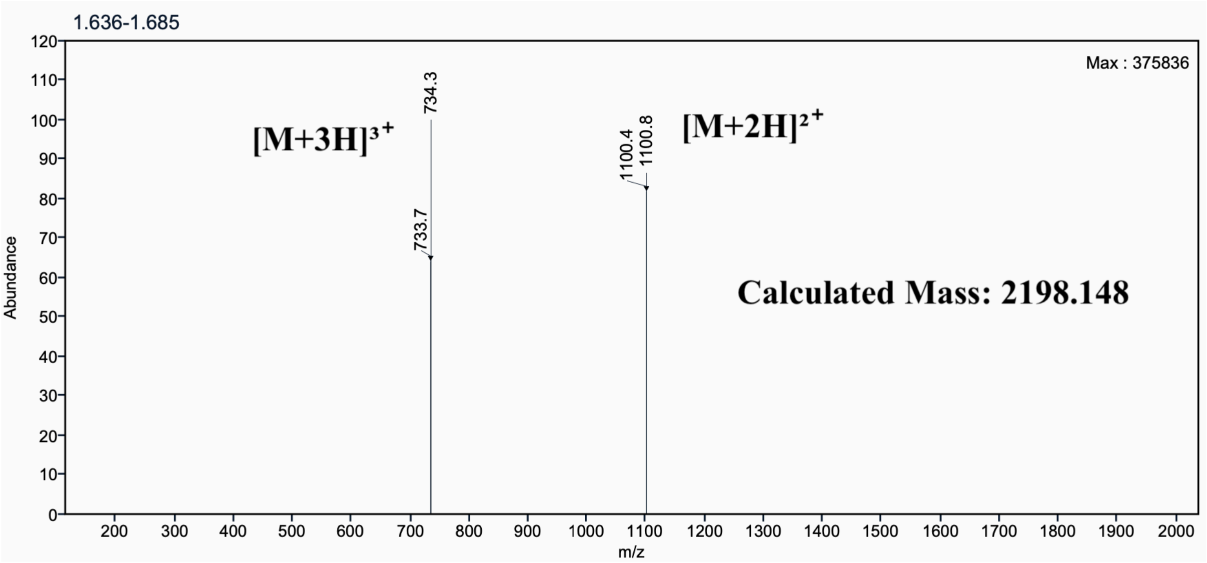

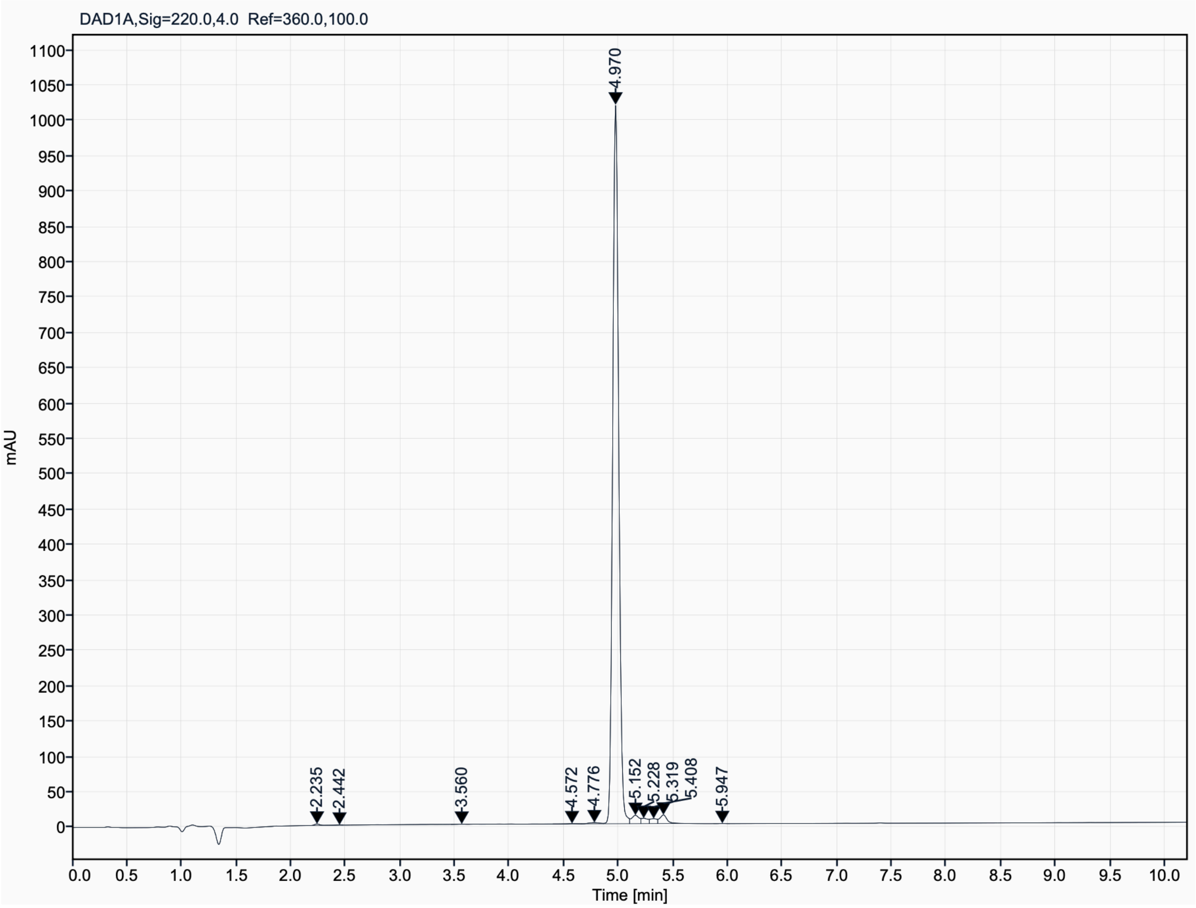

Supplementary Figure 41: Analytical UPLC and mass spectrum of MDB_D10
Sequence: cyclo[WSKMAGGAPLPGTPFAKE]

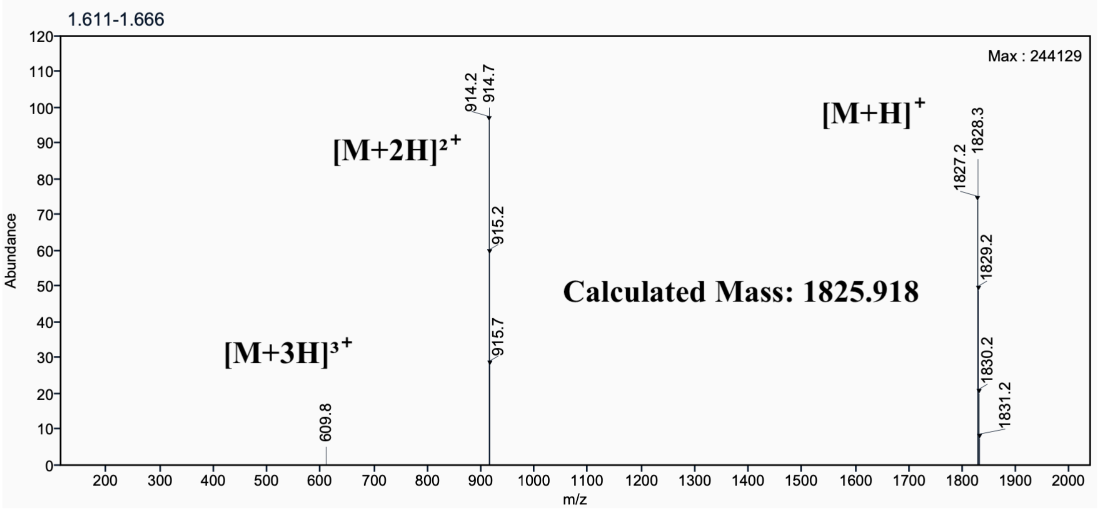

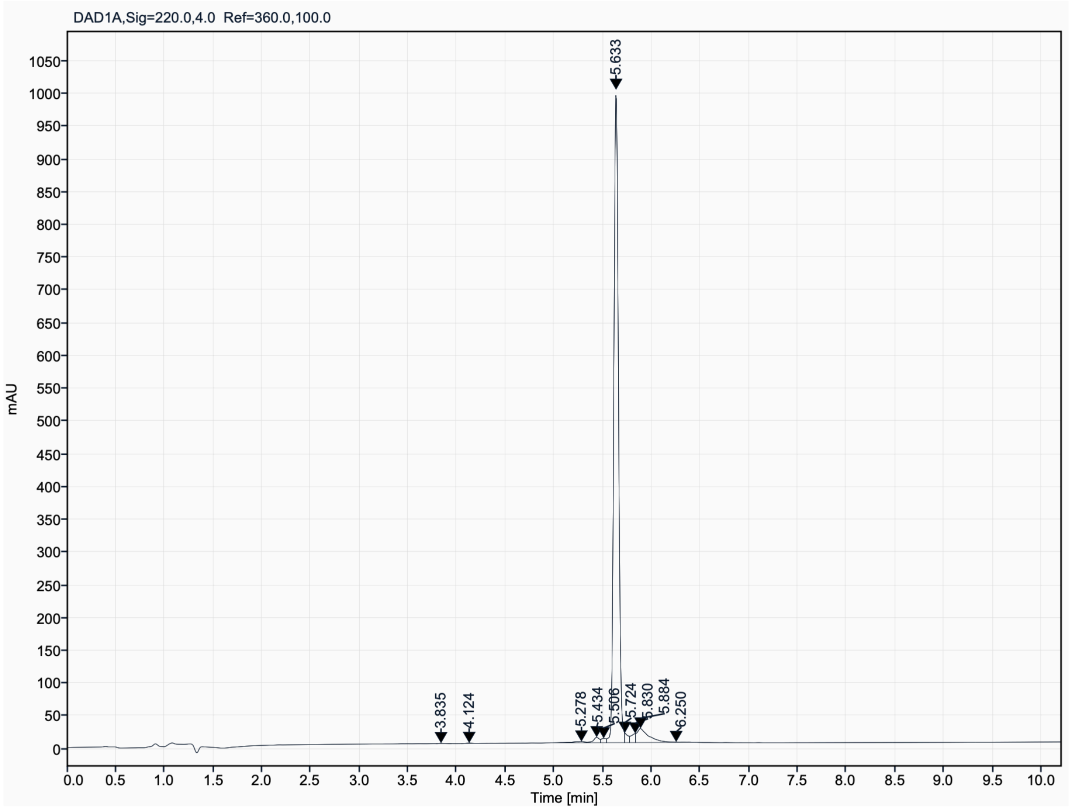

Supplementary Figure 42: Analytical UPLC and mass spectrum of MDB_D11
Sequence: cyclo[EWQKMVGLDLETDSEFAK]

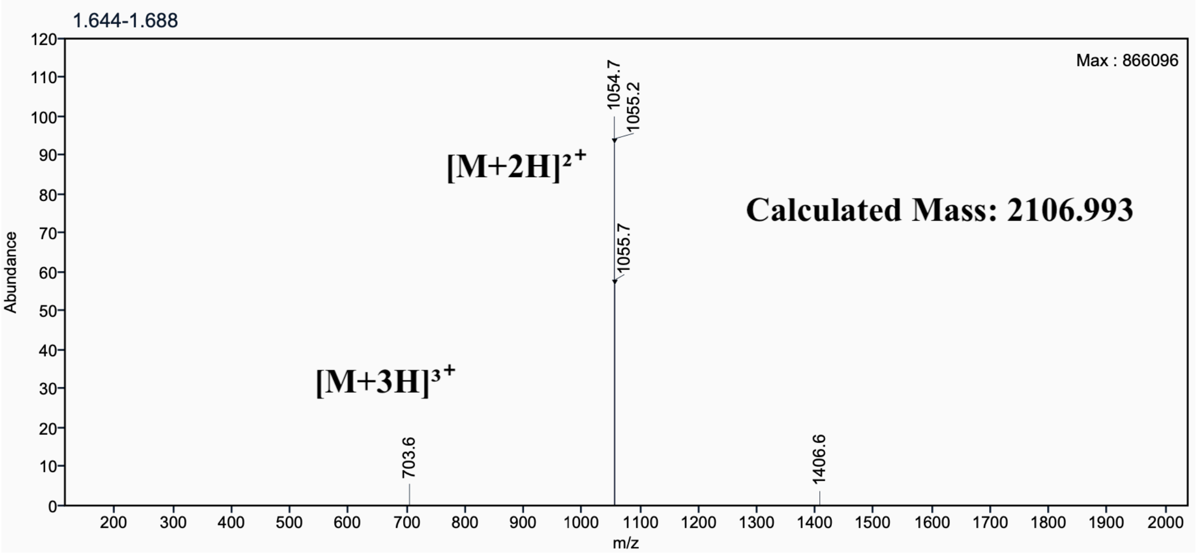

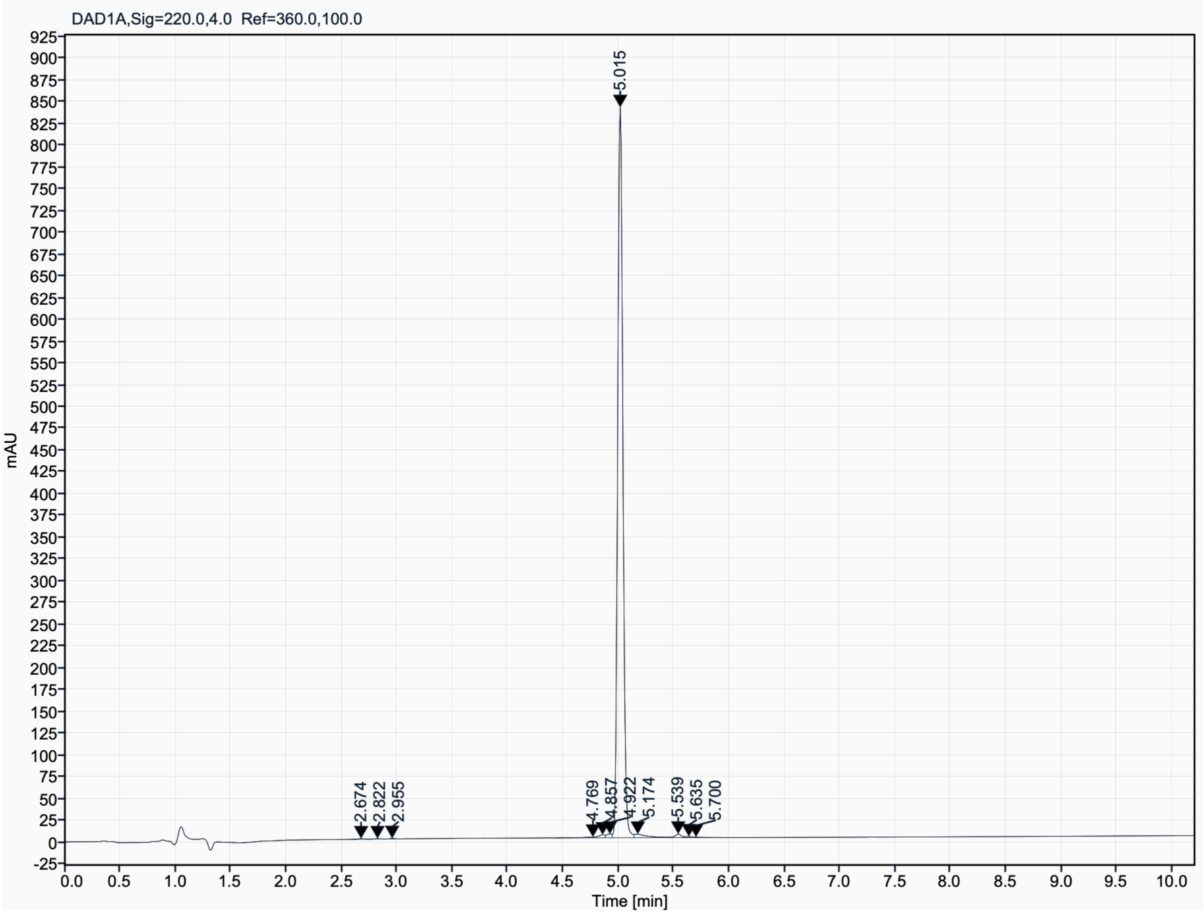

GAB peptides Mass Spec and HPLC

Supplementary Figure 43: Analytical HPLC and mass spectrum of GAB_D1
Sequence: cyclo[ETGEVENIDGVEIIYP]

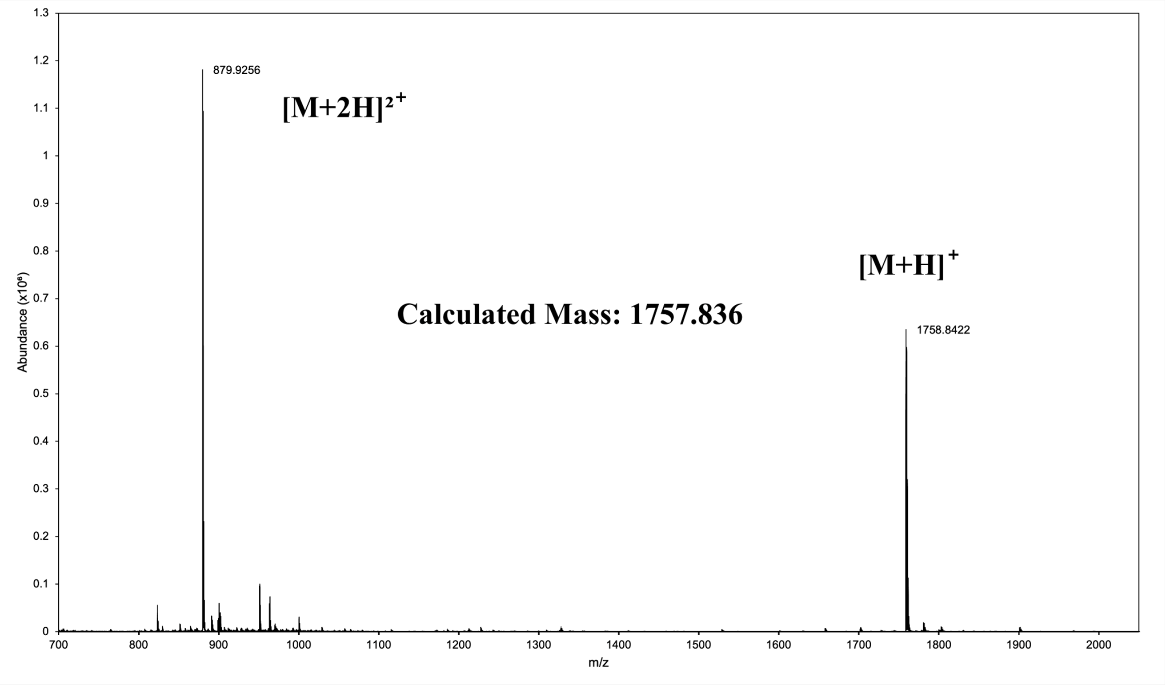

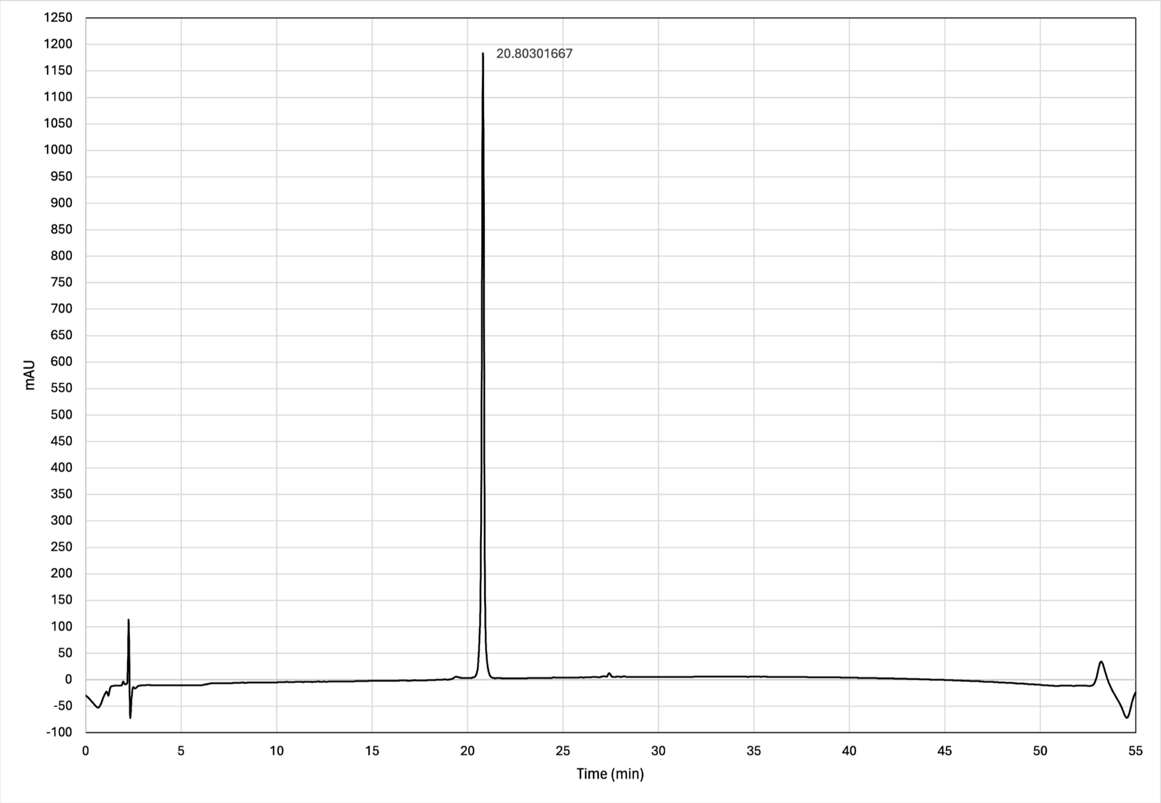

Supplementary Figure 44: Analytical HPCL and mass spectrum of GAB_D8
Sequence: cyclo[GSEYEEDGWTVLEPD]

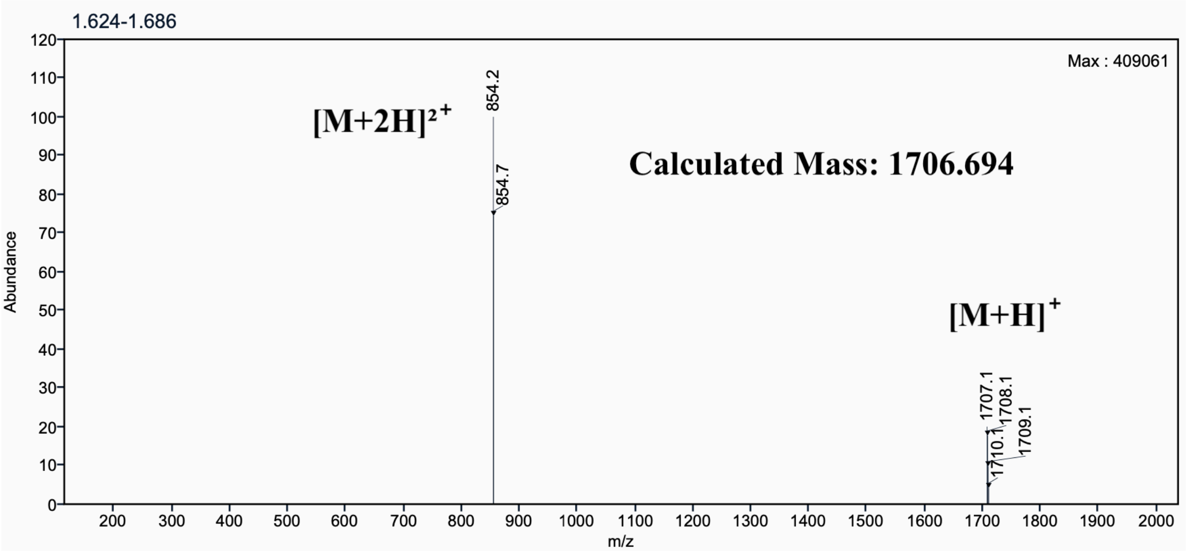

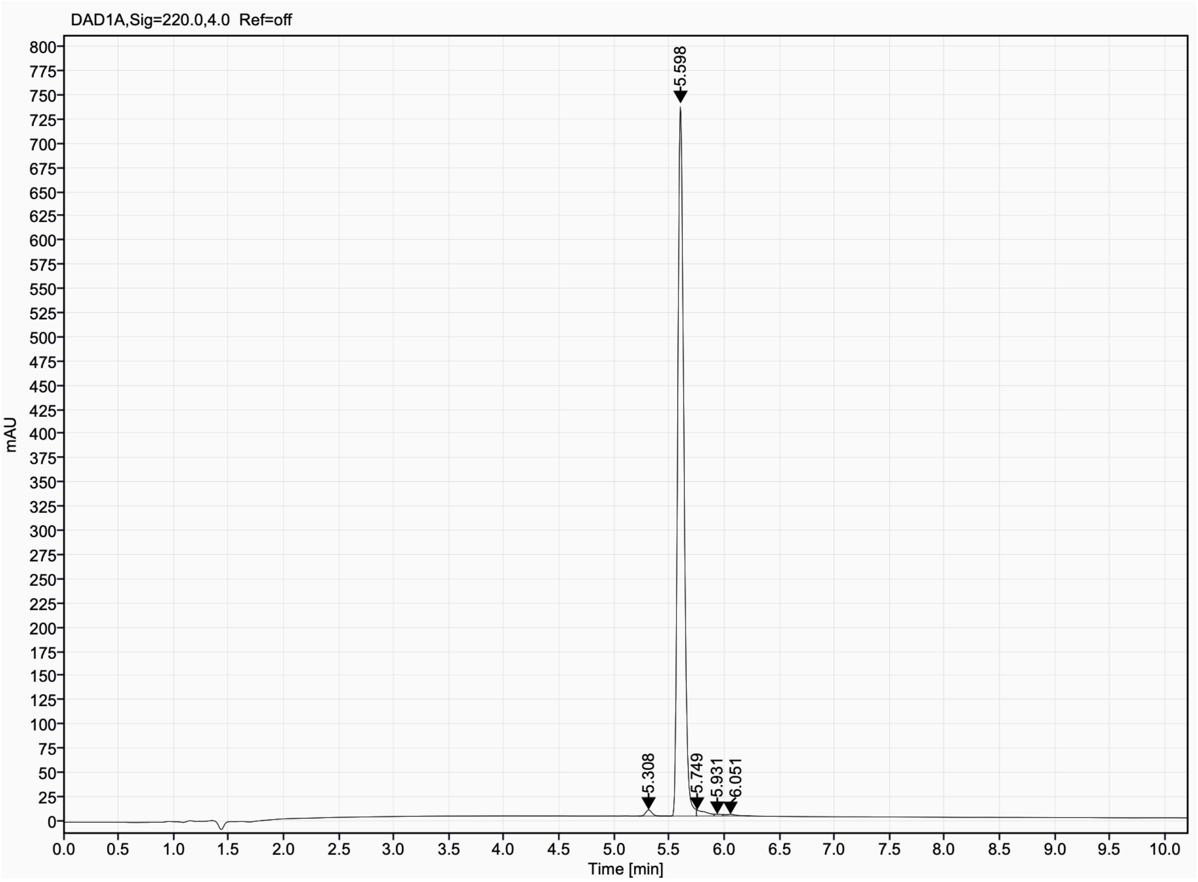

Supplementary Figure 45: Analytical HPLC and mass spectrum of GAB_D23
Sequence: cyclo[LEDGWVDIETGKE]

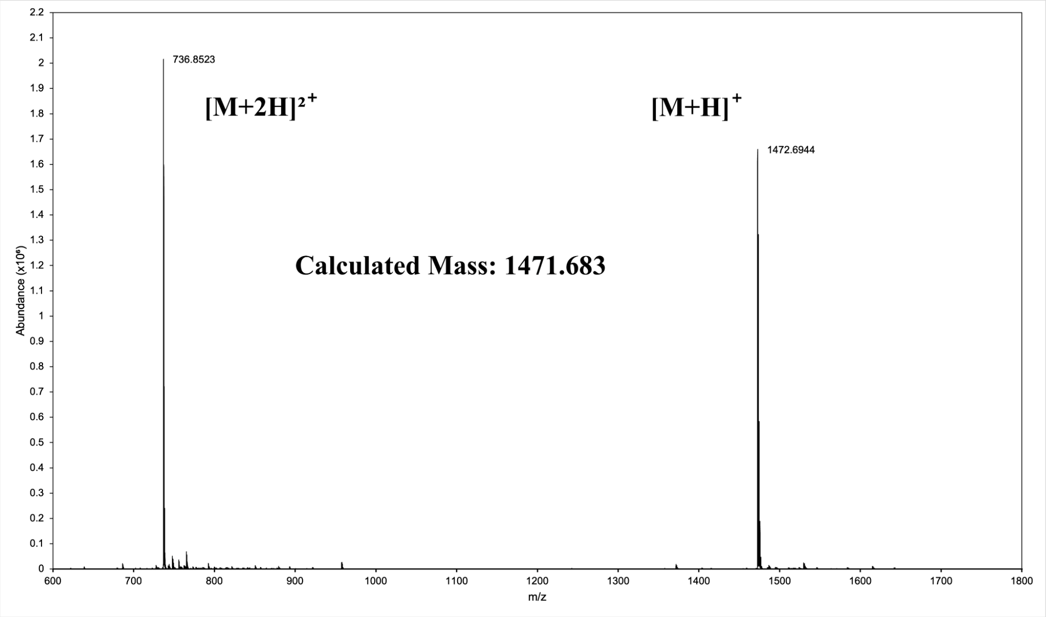

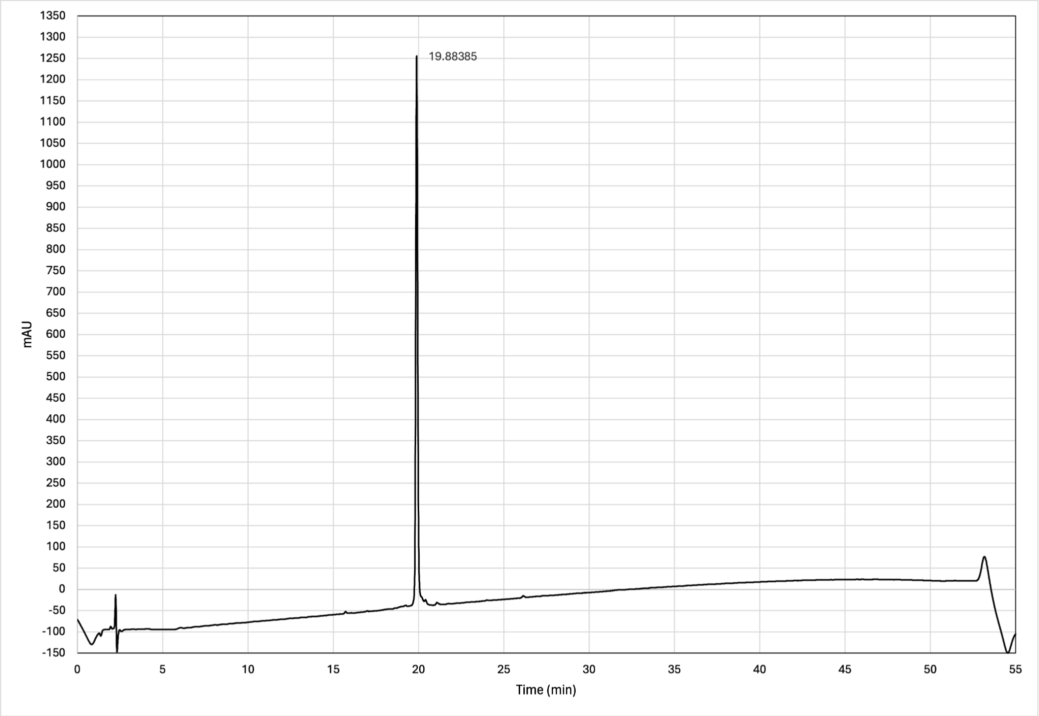

Supplementary Figure 46: Analytical HPLC and mass spectrum of GAB_D26
Sequence: cyclo[LDTGEVYKAPNGQEVIE]

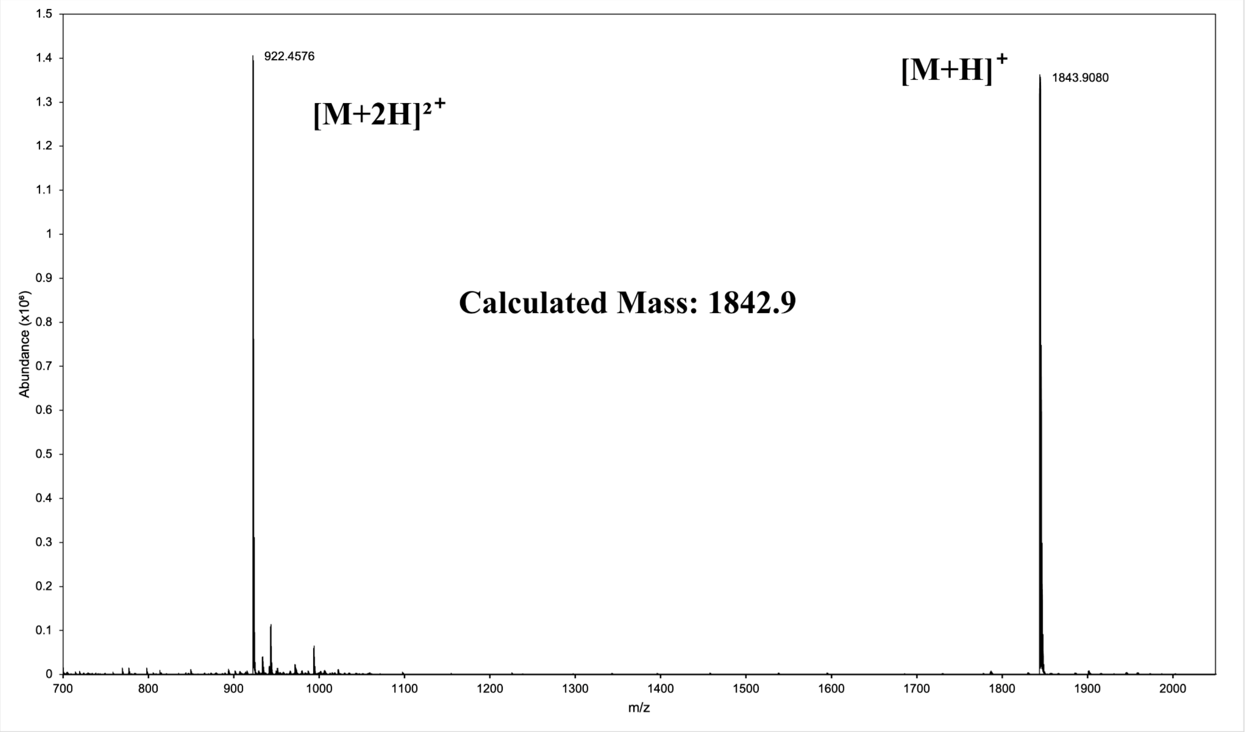

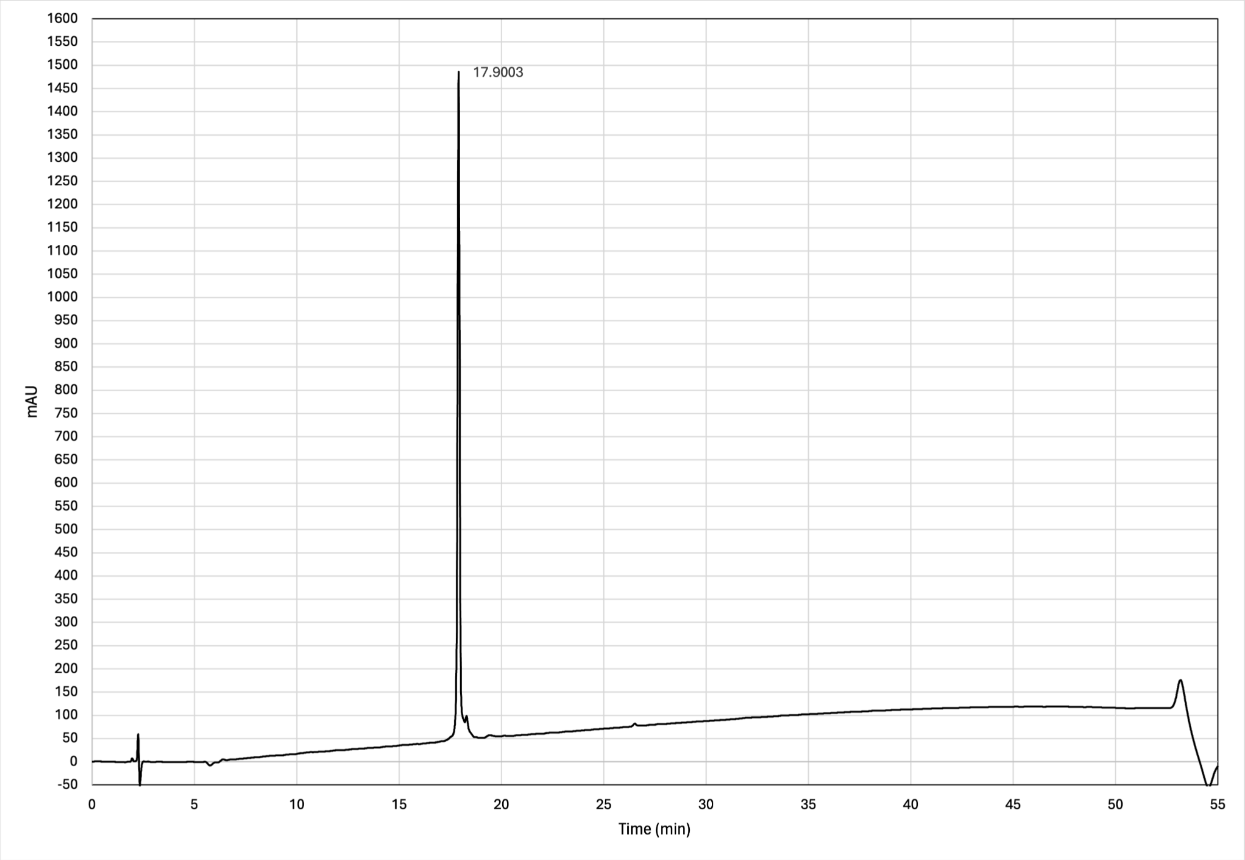

Supplementary Figure 47: Analytical UPLC and mass spectrum of GAB_D27

Sequence: cyclo[IDIDTEEEVMPGV]

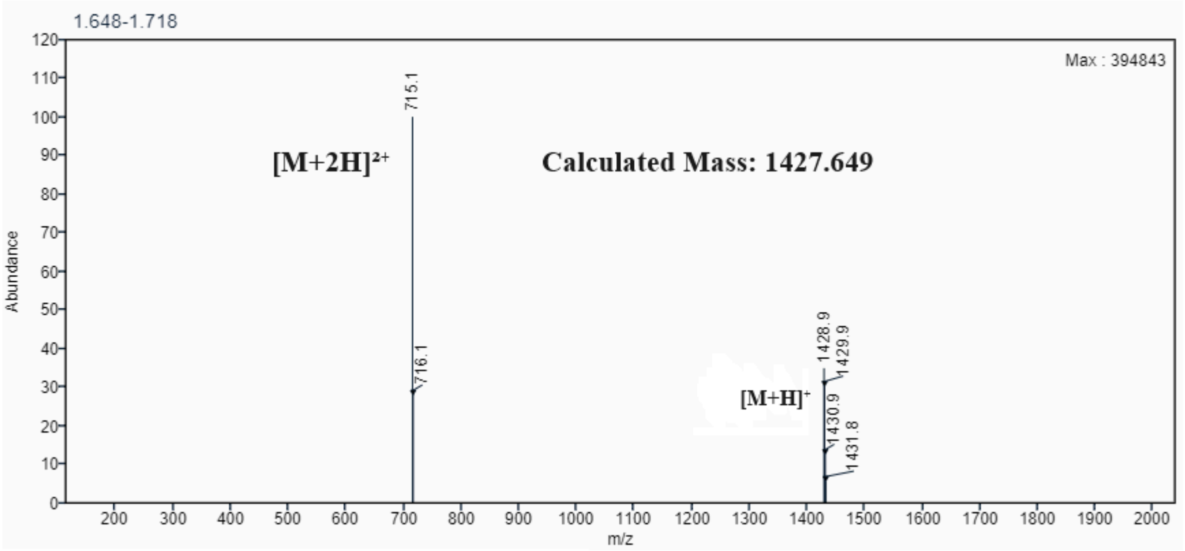

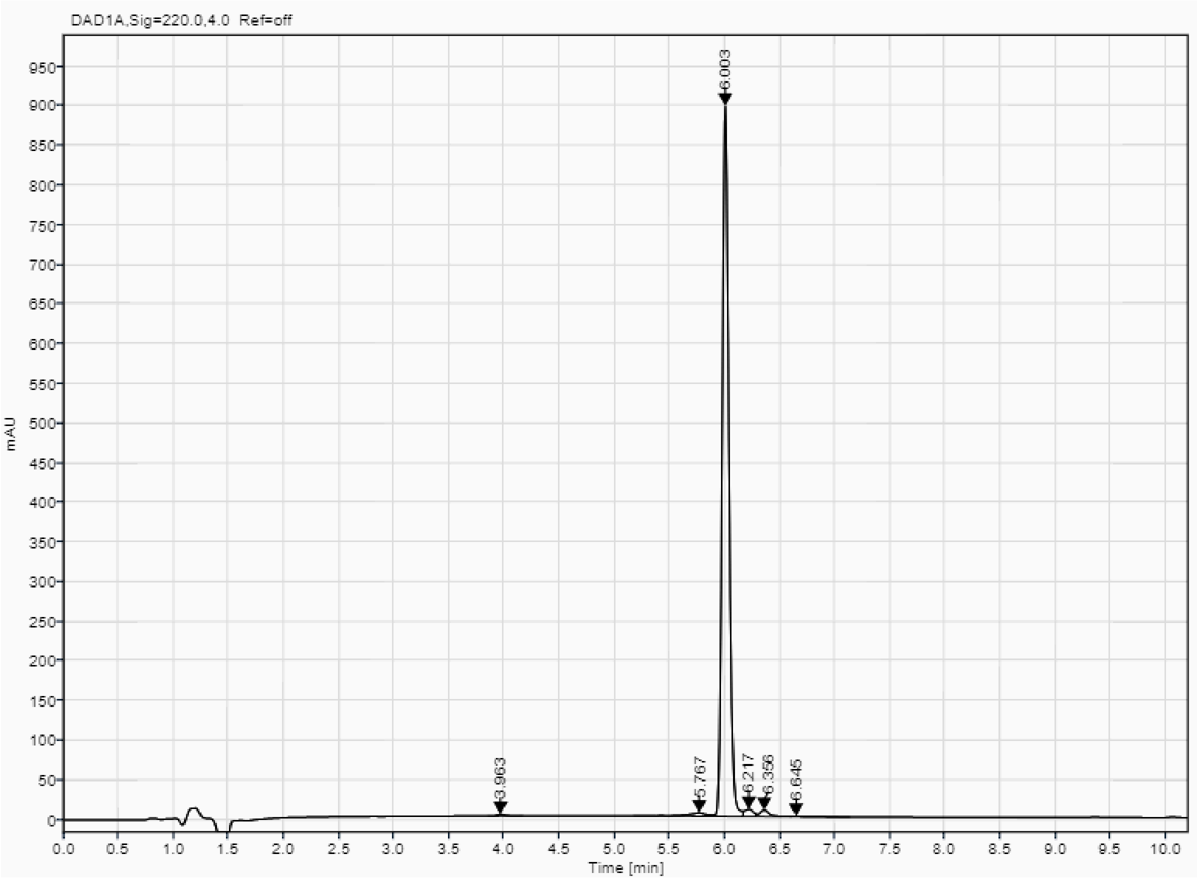

Supplementary Figure 48: Analytical UPLC and mass spectrum of GAB_D28

Sequence: cyclo[VMPGIIDIDTEEE]

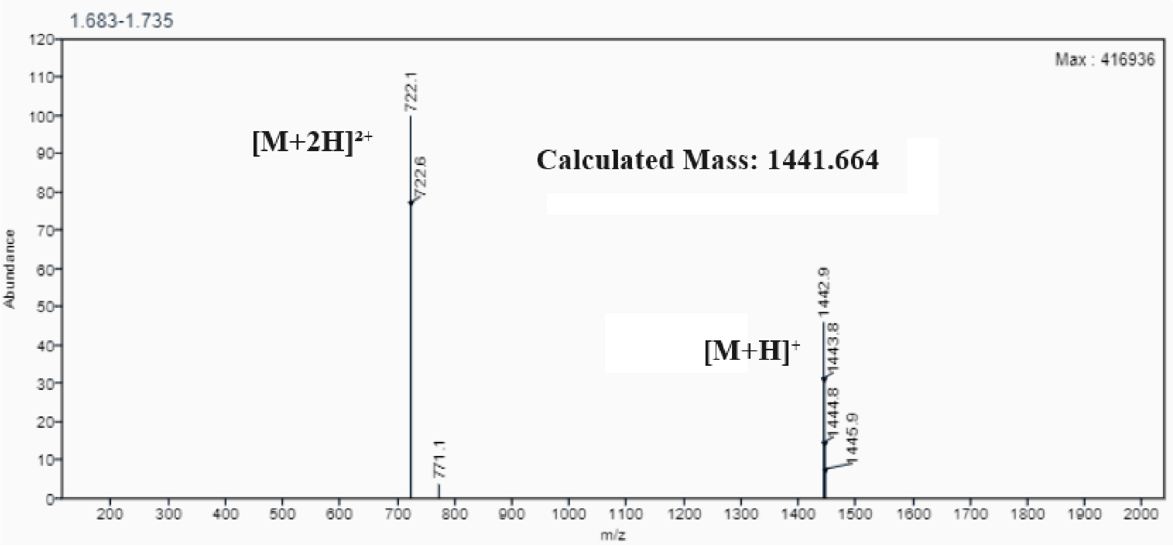

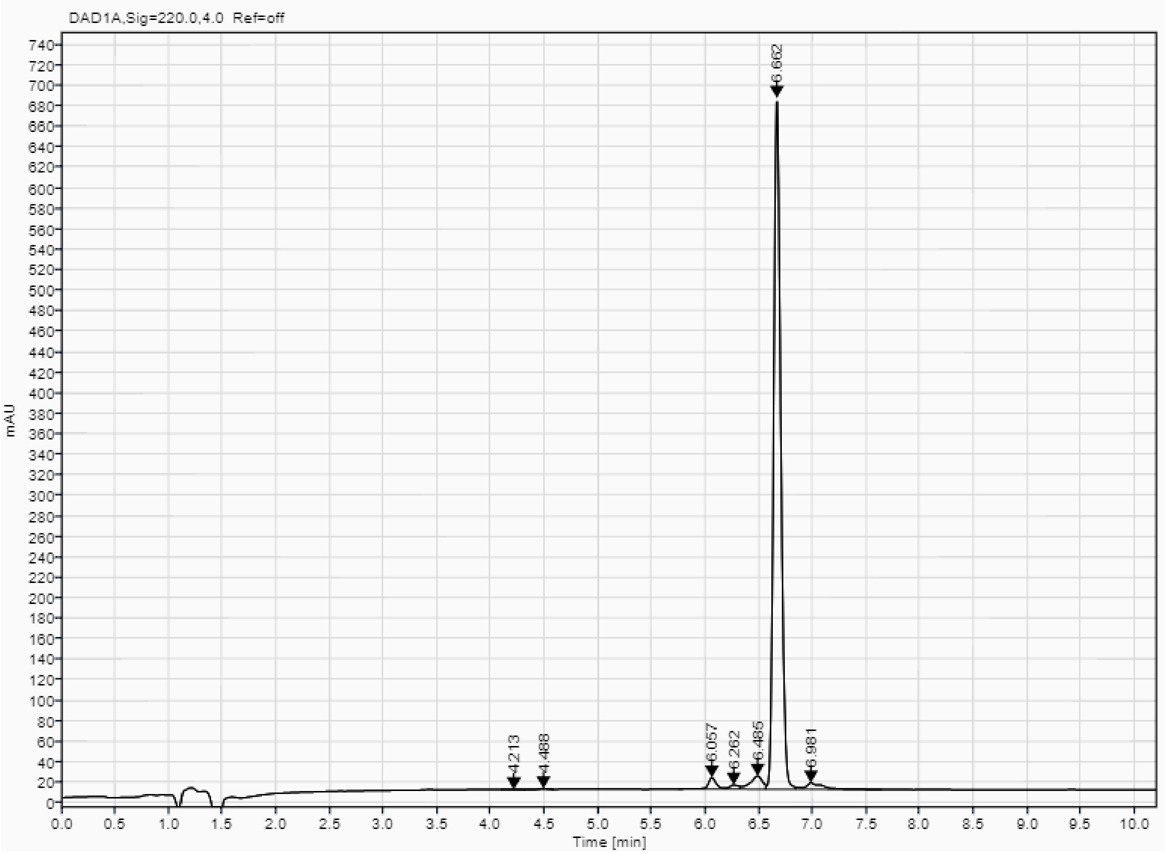

RBB peptides Mass Spec and HPLC

Supplementary Figure 49: Analytical UPLC and mass spectrum of RBB_D1
Sequence: cyclo[SSLPEYVRDVLGV]

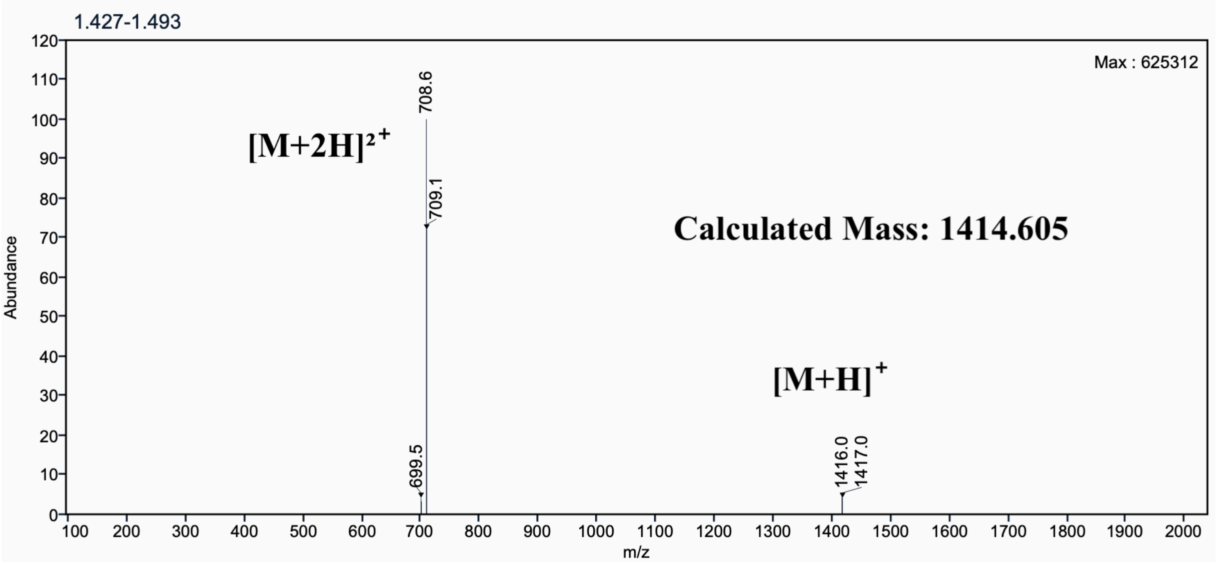

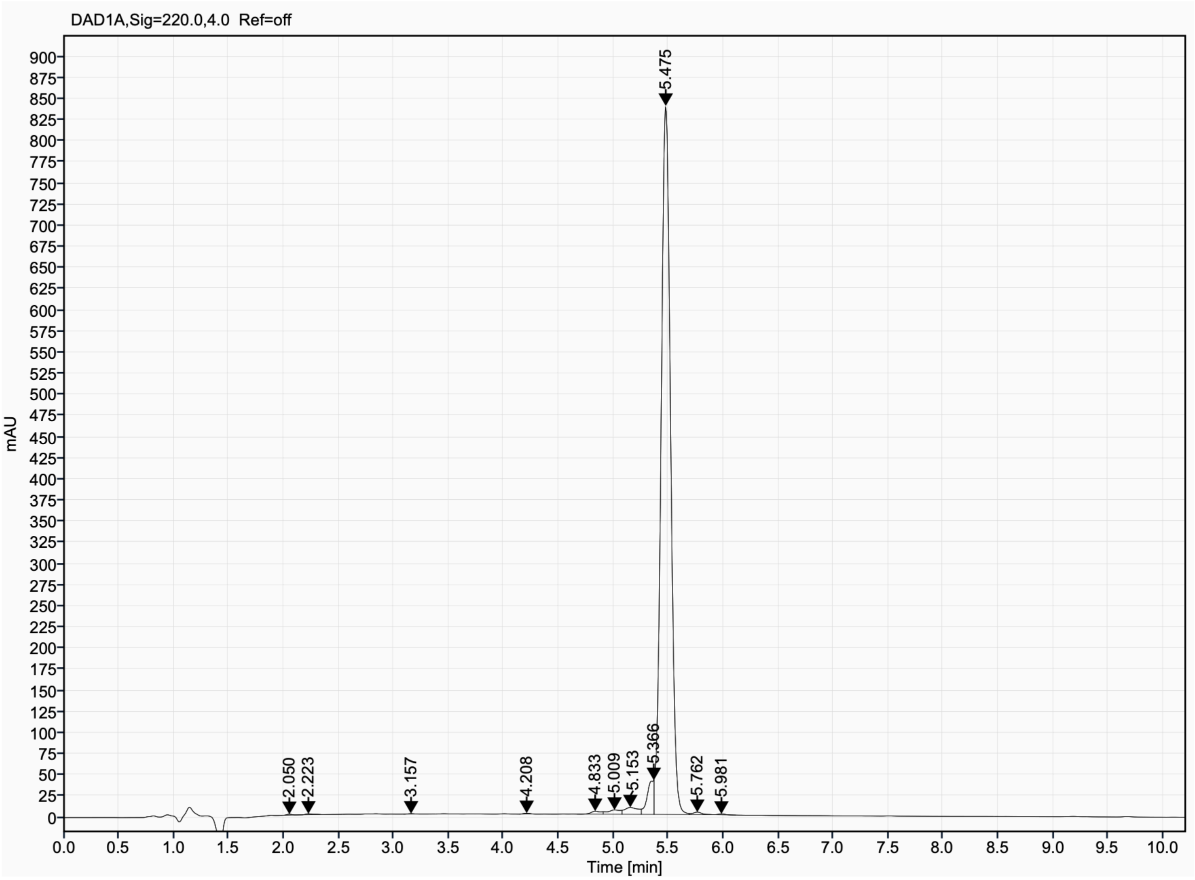

Supplementary Figure 50: Analytical UPLC and mass spectrum of RBB_D2
Sequence: cyclo[GKPGDSPWEKIESVVFG]

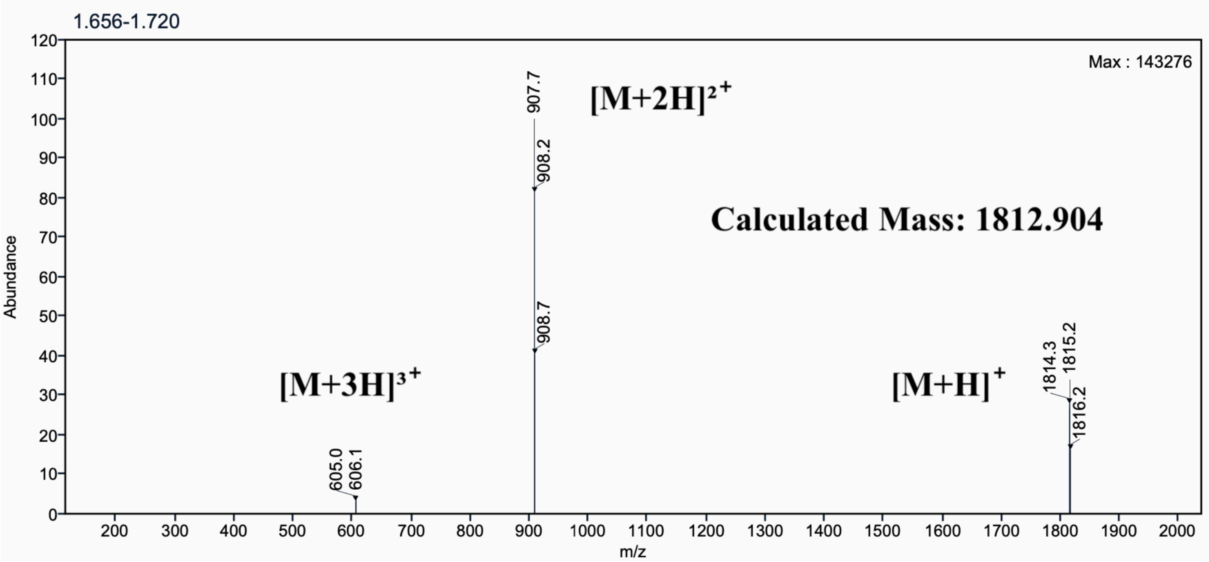

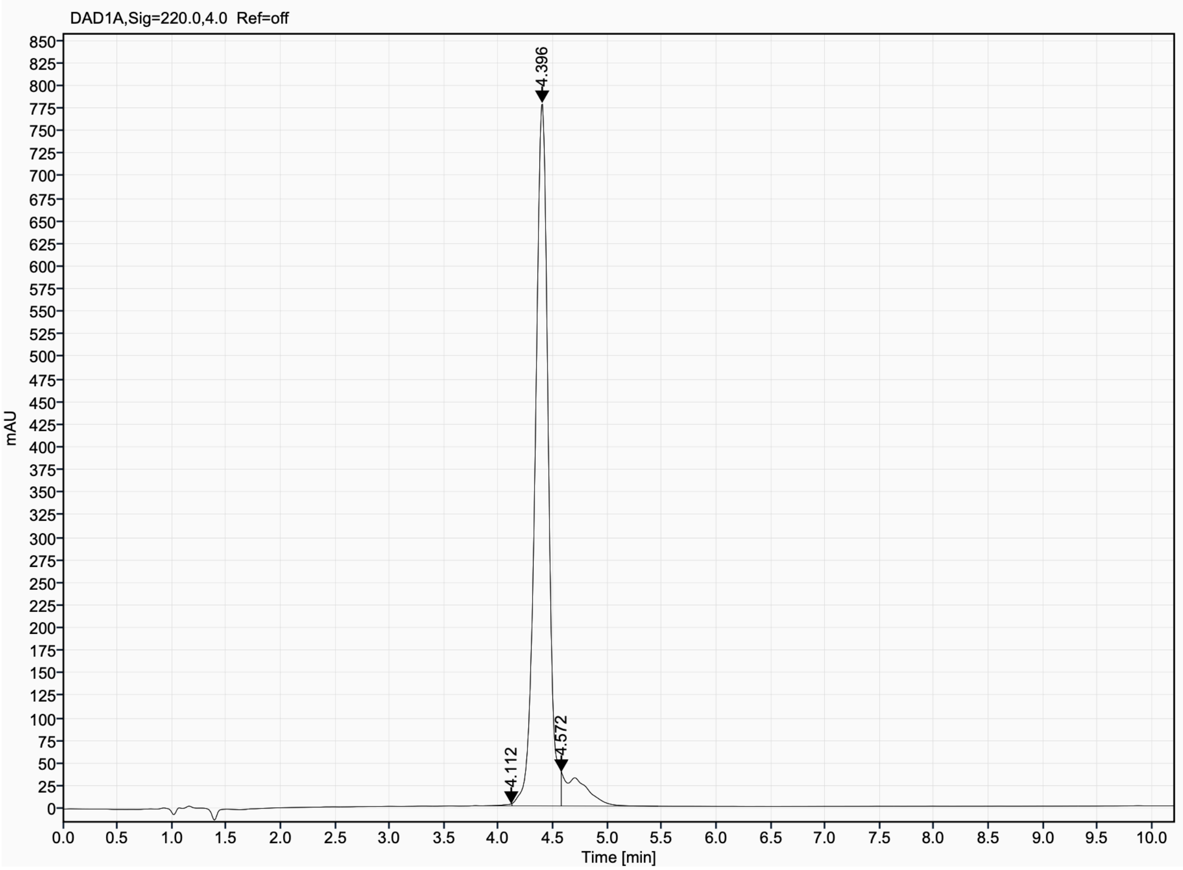

Supplementary Figure 51: Analytical UPLC and mass spectrum of RBB_D3
Sequence: cyclo[PDPAIPENVQKVFKEHML]

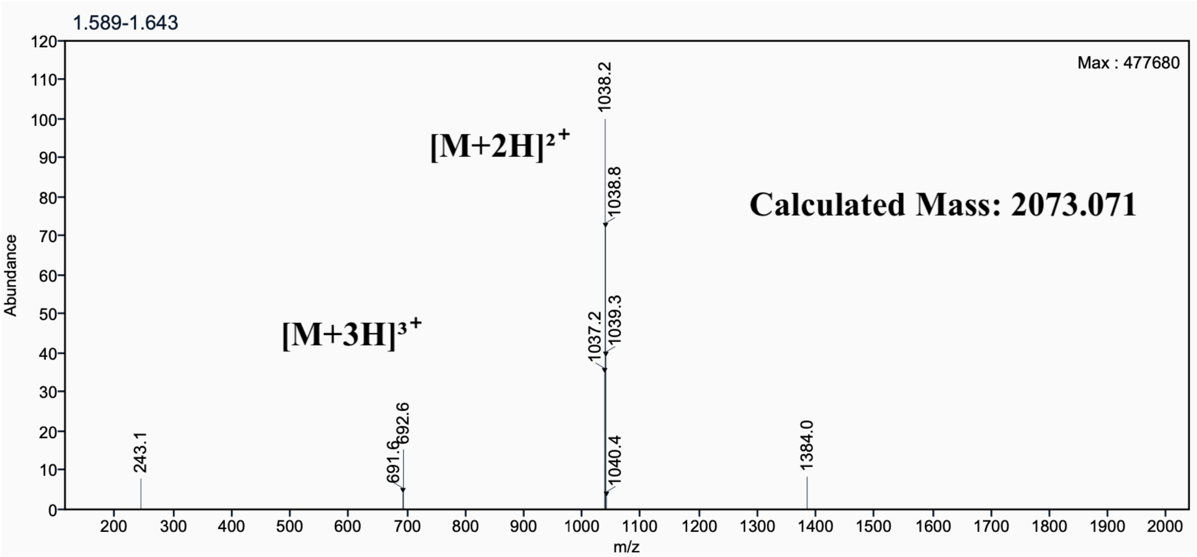

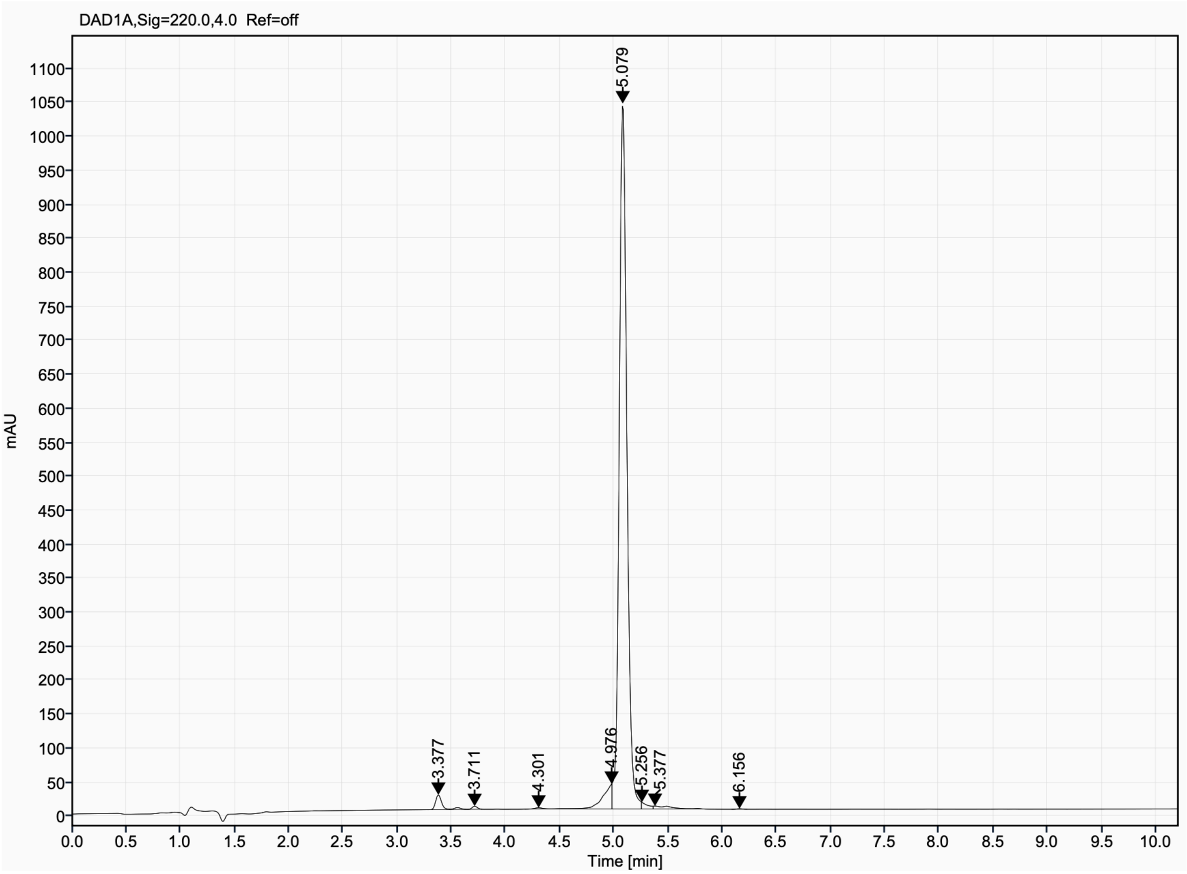

Supplementary Figure 52: Analytical UPLC and mass spectrum of RBB_D4
Sequence: cyclo[PDPAFPENVQKFKDKIE]

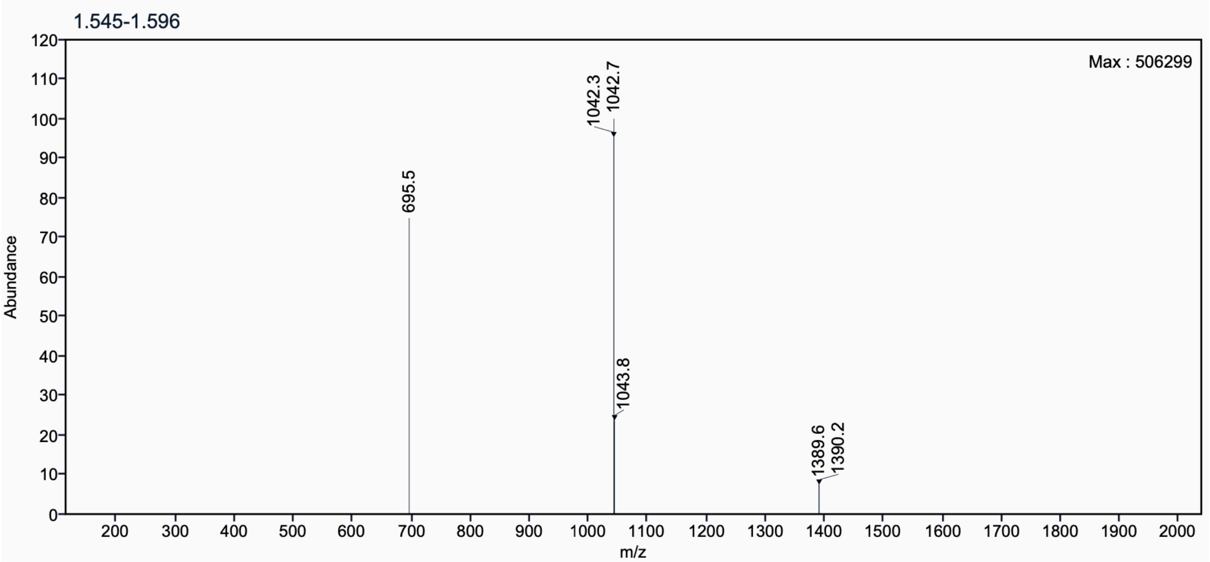

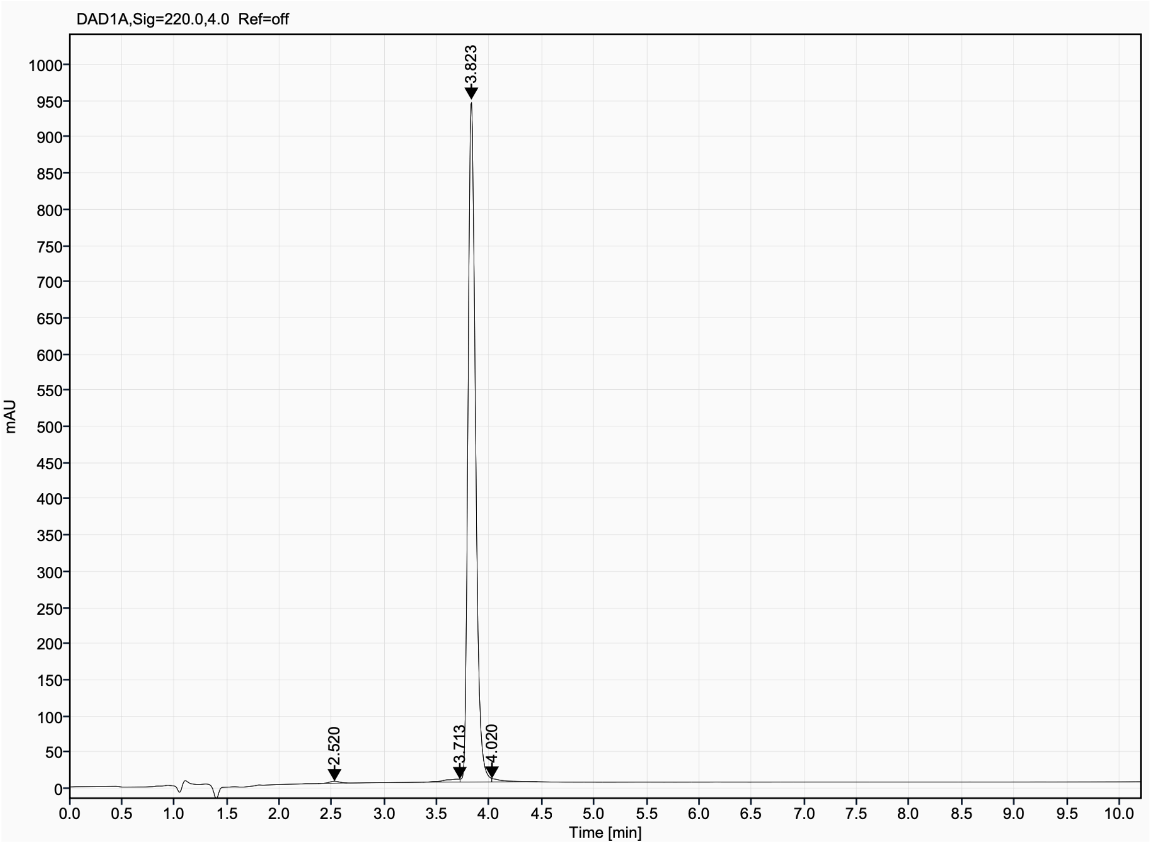

Supplementary Figure 53: Analytical UPLC and mass spectrum of RBB_D5
Sequence: cyclo[PDPAFPESIHKVFKDKII]

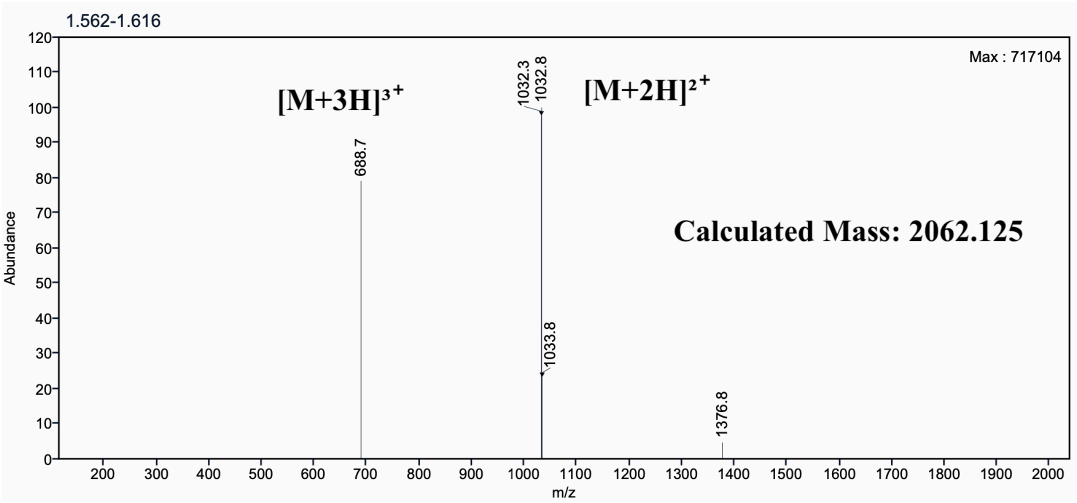

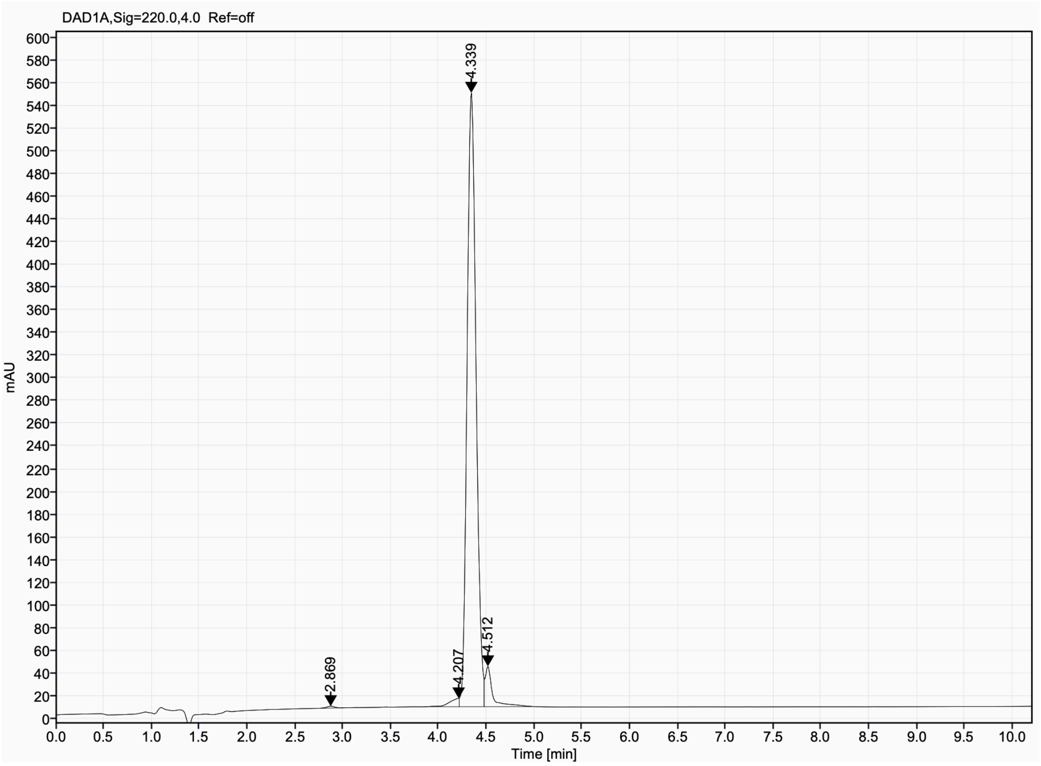

Supplementary Figure 54: Analytical UPLC and mass spectrum of RBB_D6
Sequence: cyclo[FSKYEPVDDSYPEGIRKV]

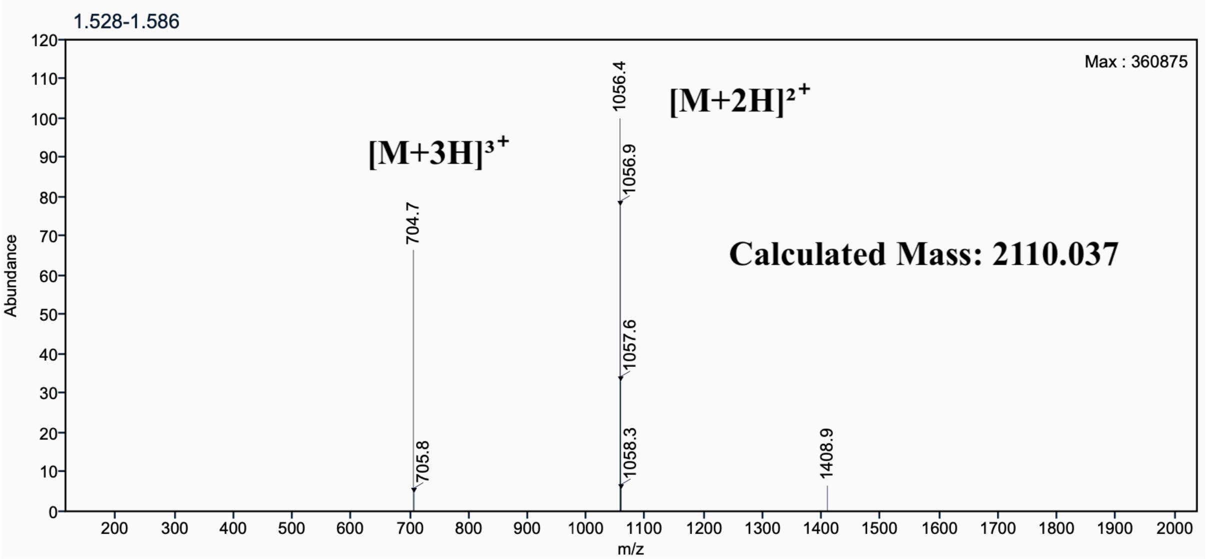

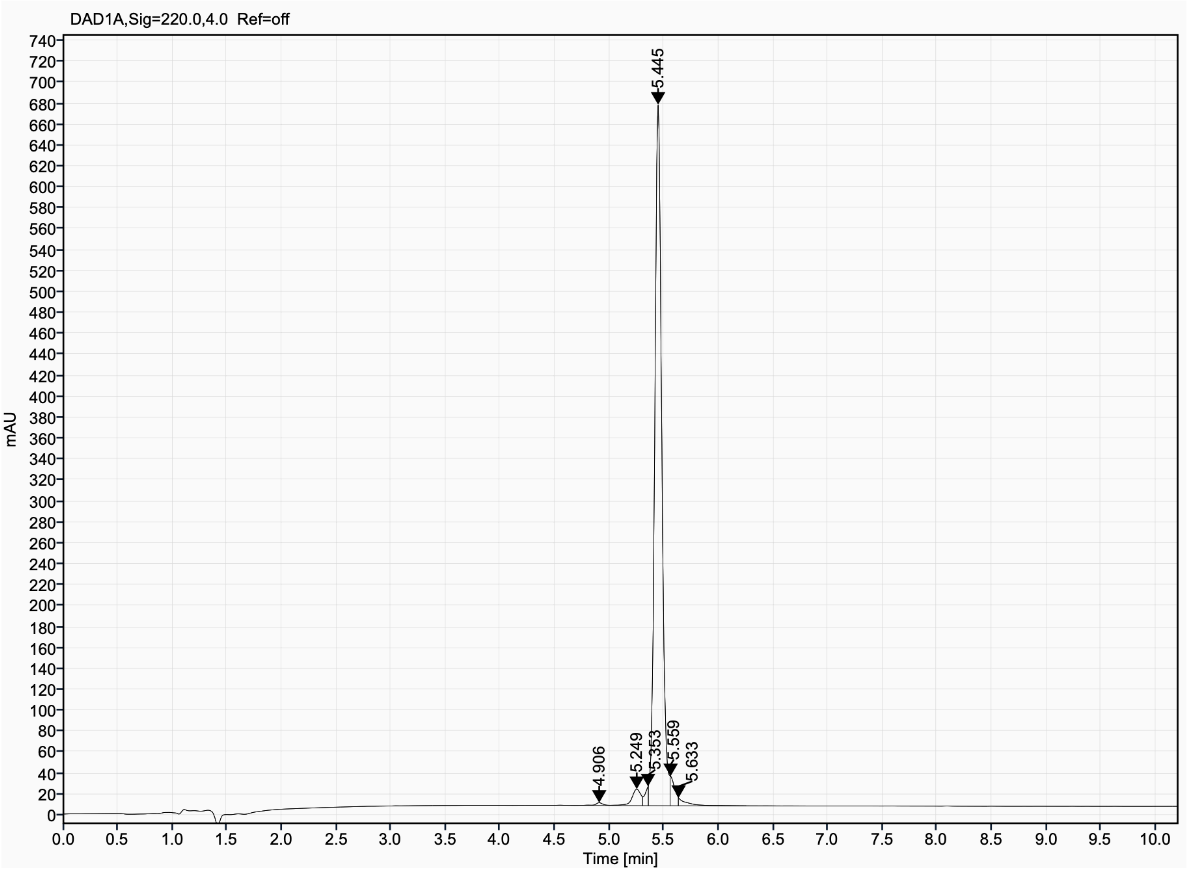

Supplementary Figure 55: Analytical UPLC and mass spectrum of RBB_D7
Sequence: cyclo[FEKYDPMDESYPENIRKV]

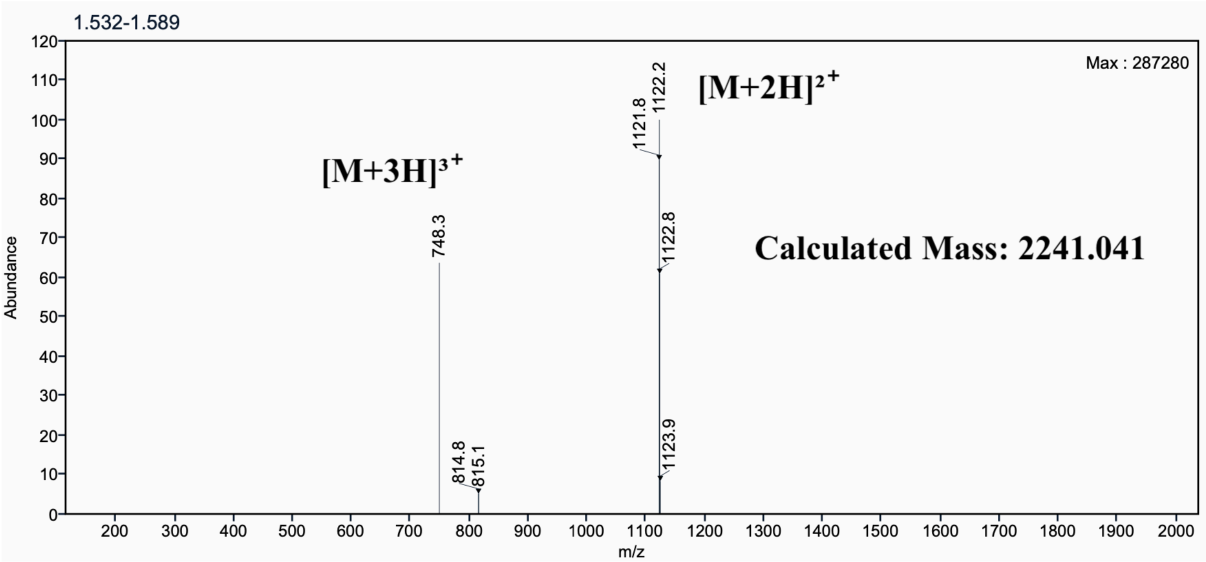

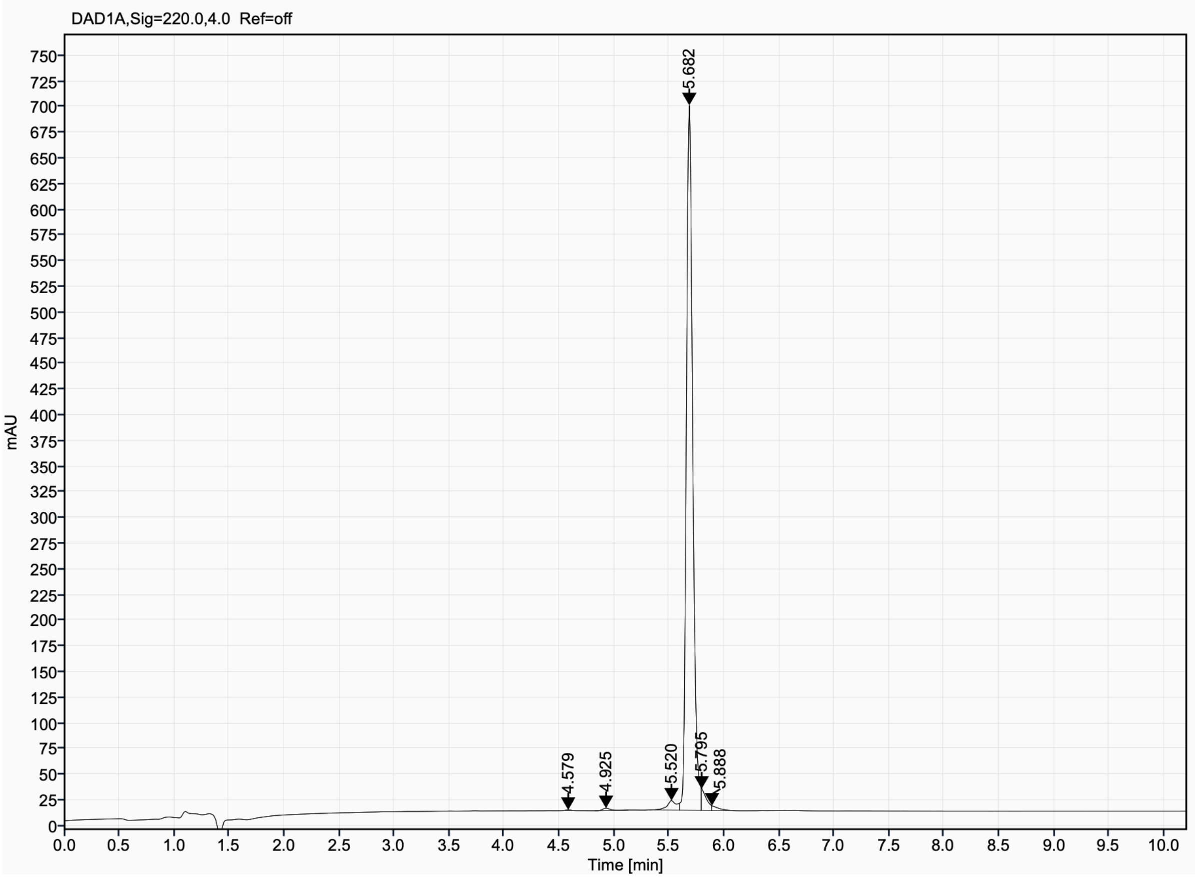

Supplementary Figure 56: Analytical UPLC and mass spectrum of RBB_D8
Sequence: cyclo[VEGNDKGSDIYEFIRDK]

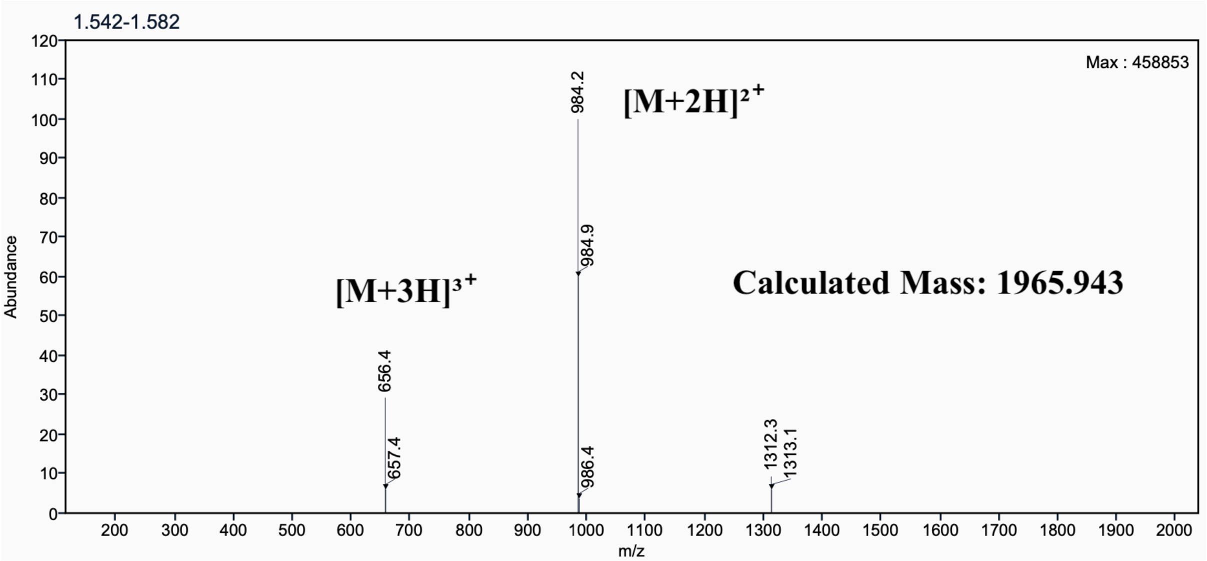

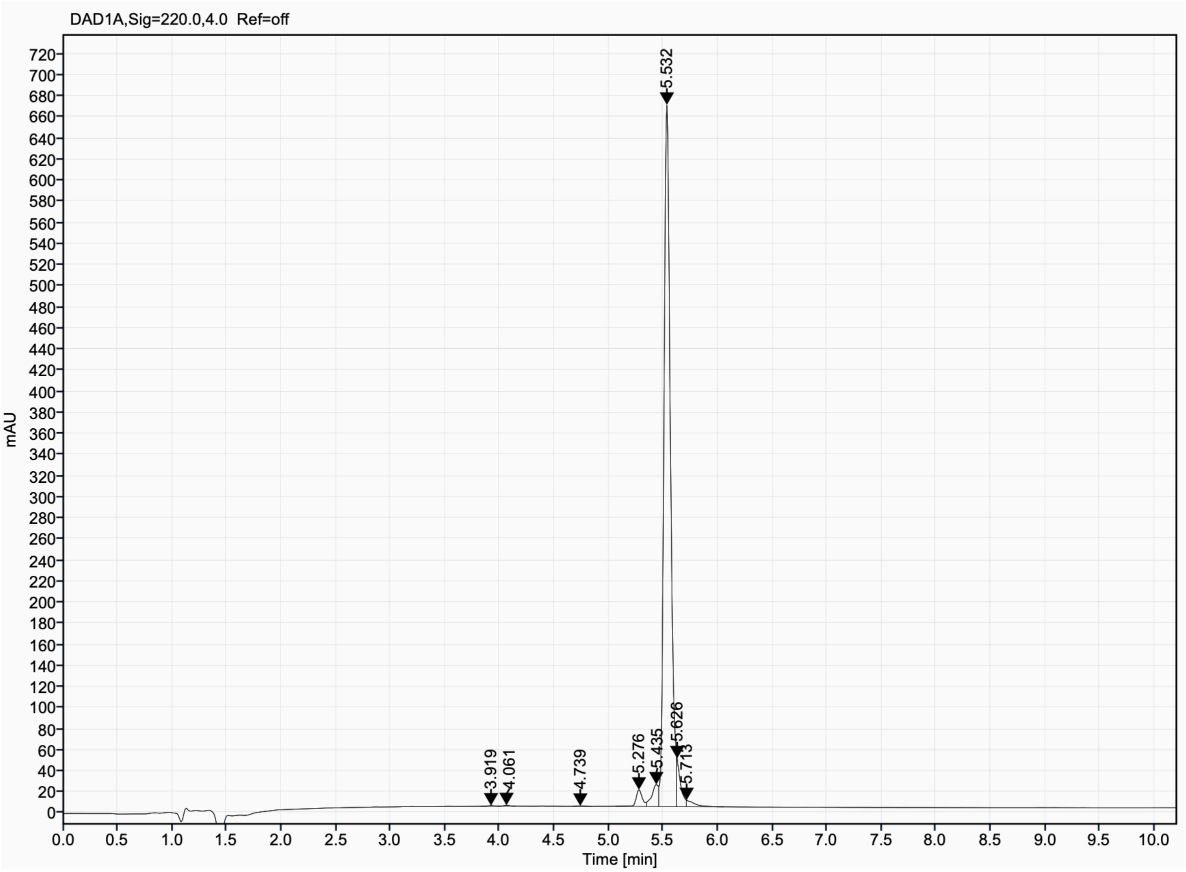

Supplementary Figure 57: Analytical UPLC and mass spectrum of RBB_D10
Sequence: cyclo[KLFGPDPYLPENVQ]

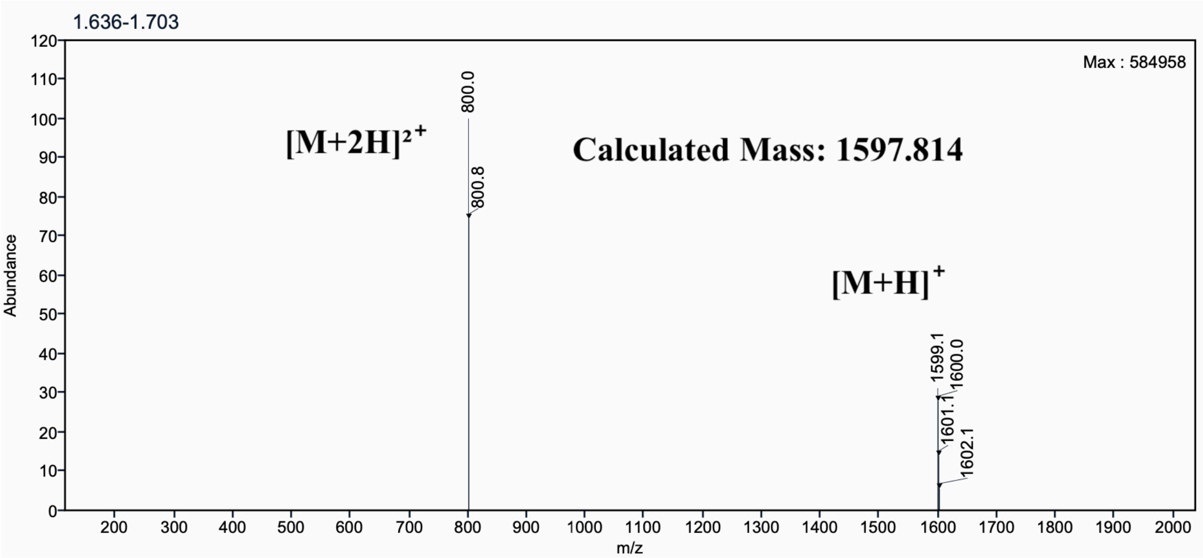

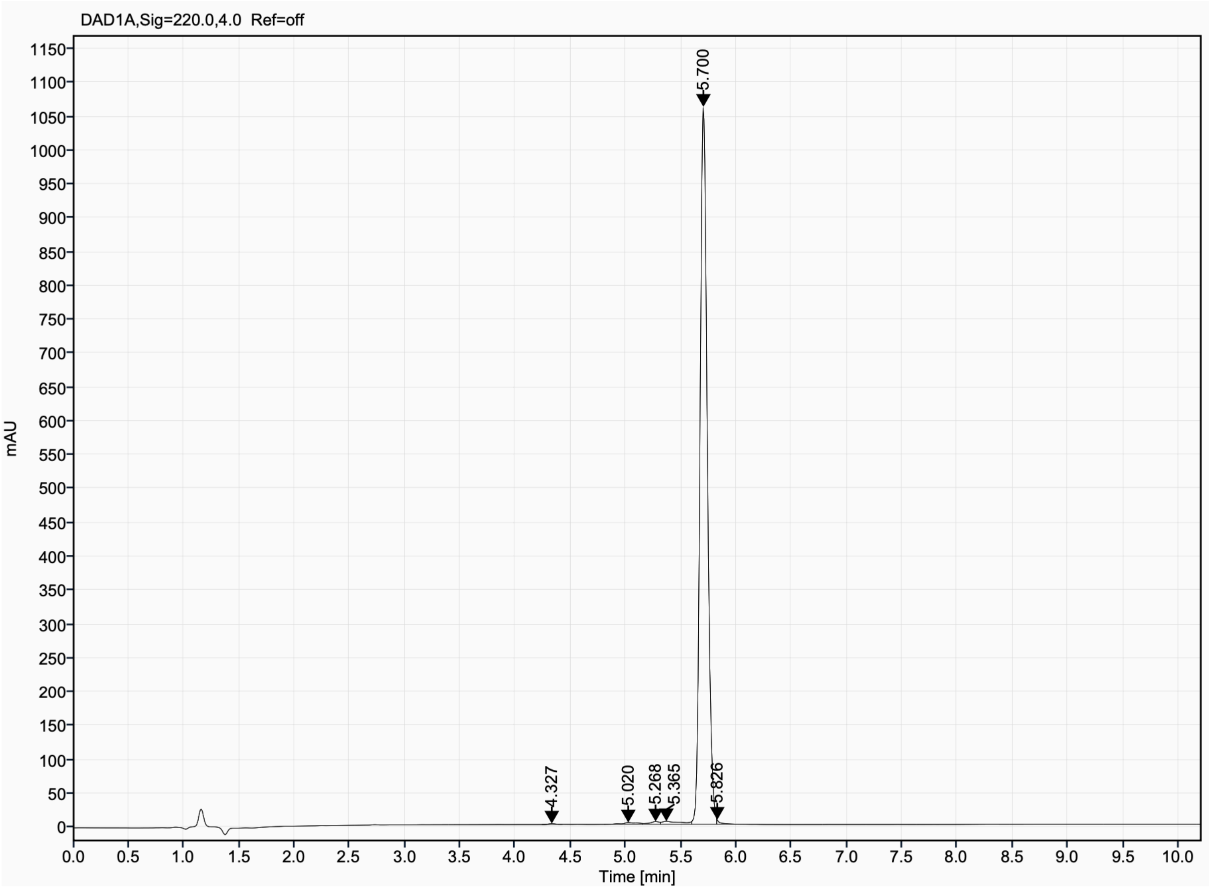

Supplementary Figure 58: Analytical UPLC and mass spectrum of RBB_D14
Sequence: cyclo[SVAKEIAEWIGIPSKVPP]

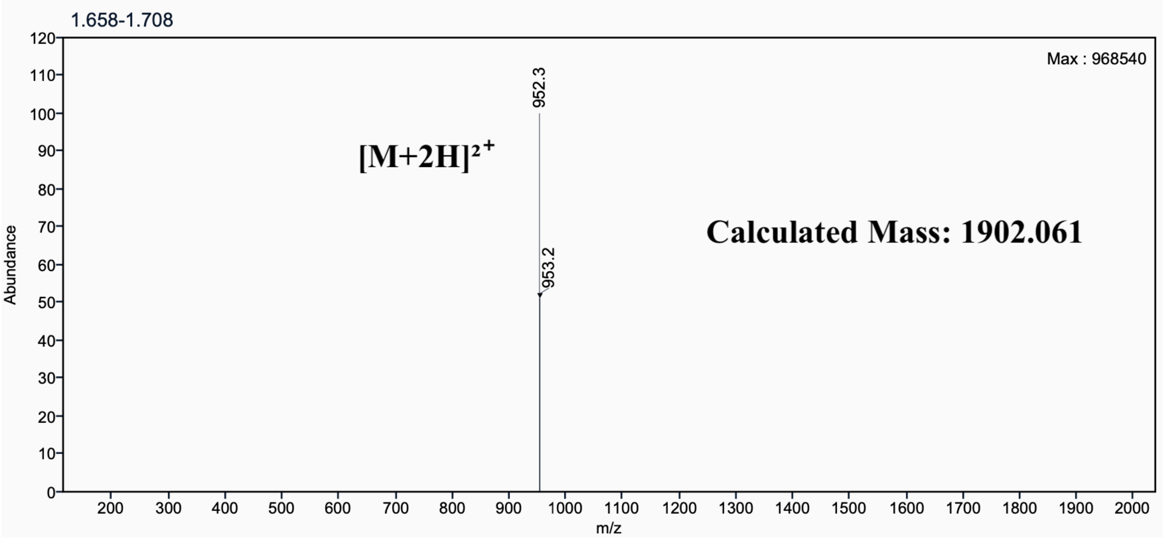

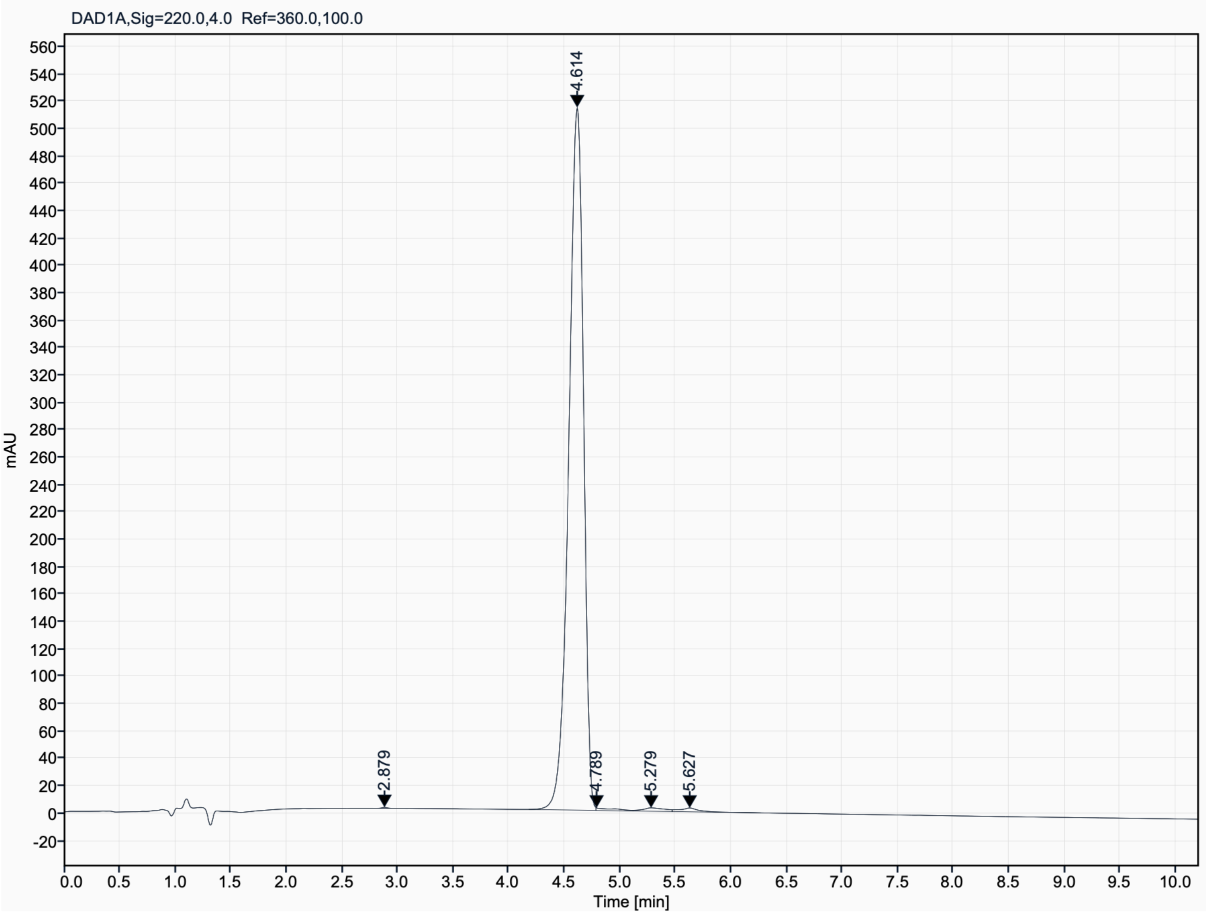

Supplementary Figure 59: Analytical UPLC and mass spectrum of RBB_D15
Sequence: cyclo[SLAKEIADWIGIPSSVPP]

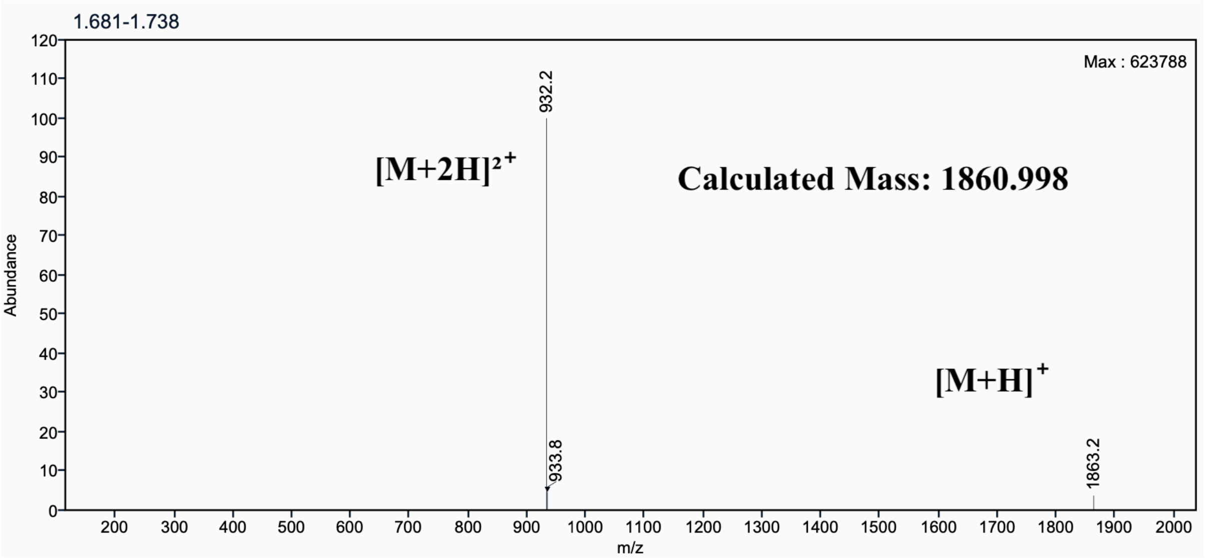

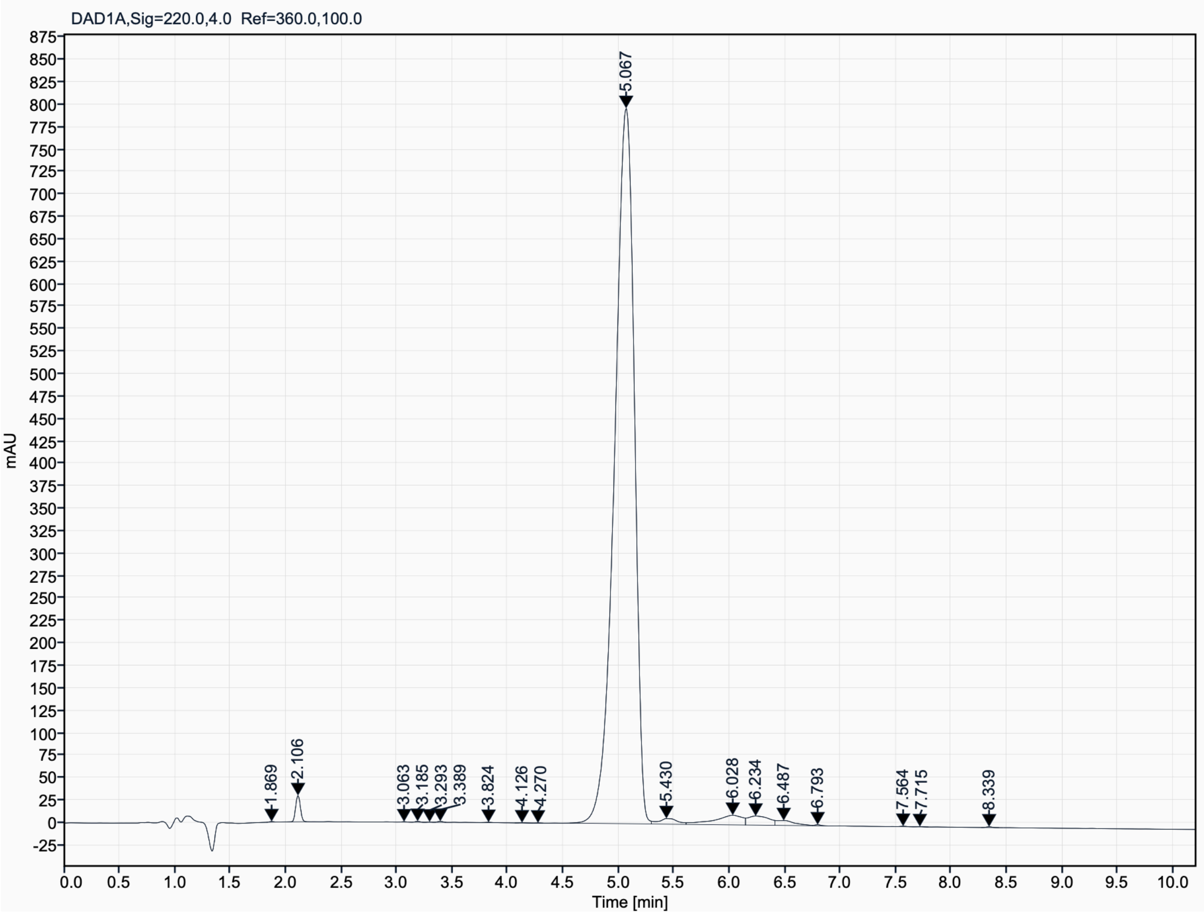

5. Supplementary References

1. Hosseinzadeh, P. *et al.* Comprehensive computational design of ordered peptide macrocycles. *Science* **358**, 1461–1466 (2017).
2. Kabsch, W. & Sander, C. Dictionary of protein secondary structure: pattern recognition of hydrogen-bonded and geometrical features. *Biopolymers* **22**, 2577–2637 (1983).
3. Fire, E., Gullá, S. V., Grant, R. A. & Keating, A. E. Mcl-1-Bim complexes accommodate surprising point mutations via minor structural changes. *Protein Sci.* **19**, 507–519 (2010).
4. Lee, T. *et al.* Discovery and biological characterization of potent myeloid cell leukemia-1 inhibitors. *FEBS Lett.* **591**, 240–251 (2017).
5. Kussie, P. H. *et al.* Structure of the MDM2 oncoprotein bound to the p53 tumor suppressor transactivation domain. *Science* **274**, 948–953 (1996).
6. Weiergräber, O. H. *et al.* Ligand binding mode of $GABA_A$ receptor-associated protein. *J. Mol. Biol.* **381**, 1320–1331 (2008).
7. Rettie, S. A. *et al.* Cyclic peptide structure prediction and design using AlphaFold. *bioRxiv* (2023) doi:10.1101/2023.02.25.529956.
8. Zhang, Y. & Skolnick, J. TM-align: a protein structure alignment algorithm based on the TM-score. *Nucleic Acids Res.* **33**, 2302–2309 (2005).
9. van der Maaten, L. Visualizing Data using t-SNE. https://www.jmlr.org/papers/volume9/vandermaaten08a/vandermaaten08a.pdf?fbcl (2008).
10. Pedregosa, F. *et al.* Scikit-learn: Machine Learning in Python. *arXiv [cs.LG]* 2825–2830 (2012).
11. Dauparas, J. *et al.* Robust deep learning-based protein sequence design using ProteinMPNN. *Science* **378**, 49–56 (2022).
12. Jumper, J. *et al.* Highly accurate protein structure prediction with AlphaFold. *Nature* **596**, 583–589 (2021).

13. Lin, Z. *et al.* Evolutionary-scale prediction of atomic-level protein structure with a language model. *Science* **379**, 1123–1130 (2023).
14. Baek, M. *et al.* Accurate prediction of protein structures and interactions using a three-track neural network. *Science* **373**, 871–876 (2021).
15. Wang, J. *et al.* Scaffolding protein functional sites using deep learning. *Science* **377**, 387–394 (2022).
16. Watson, J. L. *et al.* De novo design of protein structure and function with RFdiffusion. *Nature* **620**, 1089–1100 (2023).
17. Sternberg, A. MaxCluster: a tool for protein structure comparison and clustering. http://www.sbg.bio.ic.ac.uk/maxcluster/index.html.
18. Siew, N., Elofsson, A., Rychlewski, L. & Fischer, D. MaxSub: an automated measure for the assessment of protein structure prediction quality. *Bioinformatics* **16**, 776–785 (2000).
19. Khatib, F. *et al.* Algorithm discovery by protein folding game players. *Proc. Natl. Acad. Sci. U. S. A.* **108**, 18949–18953 (2011).
20. Bhardwaj, G. *et al.* Accurate de novo design of hyperstable constrained peptides. *Nature* **538**, 329–335 (2016).
21. Baek, M. *et al.* Efficient and accurate prediction of protein structure using RoseTTAFold2. *bioRxiv* 2023.05.24.542179 (2023) doi:10.1101/2023.05.24.542179.
22. Brown, H. *et al.* Structure-Based Design of Stapled Peptides That Bind GABARAP and Inhibit Autophagy. *J. Am. Chem. Soc.* **144**, 14687–14697 (2022).
23. Bhardwaj, G. *et al.* Accurate de novo design of membrane-traversing macrocycles. *Cell* (2022) doi:10.1016/j.cell.2022.07.019.
24. Chaudhury, S., Lyskov, S. & Gray, J. J. PyRosetta: a script-based interface for implementing molecular modeling algorithms using Rosetta. *Bioinformatics* **26**, 689–691 (2010).
25. The UniProt Consortium. UniProt: the universal protein knowledgebase. *Nucleic Acids Res.* **45**, D158–D169 (2017).

26. Mirdita, M. *et al.* ColabFold: making protein folding accessible to all. *Nat. Methods* **19**, 679–682 (2022).

27. Anil, B., Riedinger, C., Endicott, J. A. & Noble, M. E. M. The structure of an MDM2-Nutlin-3a complex solved by the use of a validated MDM2 surface-entropy reduction mutant. *Acta Crystallogr. D Biol. Crystallogr.* **69**, 1358–1366 (2013).

28. Sumida, K. H. *et al.* Improving Protein Expression, Stability, and Function with ProteinMPNN. *J. Am. Chem. Soc.* **146**, 2054–2061 (2024).

29. Bhandari, B. K., Gardner, P. P. & Lim, C. S. Solubility-Weighted Index: fast and accurate prediction of protein solubility. *Bioinformatics* **36**, 4691–4698 (2020).

30. Davis, G. D., Elisee, C., Newham, D. M. & Harrison, R. G. New fusion protein systems designed to give soluble expression in Escherichia coli. *Biotechnol. Bioeng.* **65**, 382–388 (1999).

31. Dang, B. *et al.* SNAC-tag for sequence-specific chemical protein cleavage. *Nat. Methods* **16**, 319–322 (2019).

32. Leveille, A. N. *et al.* Exploring Arylidene-Indolinone Ligands of Autophagy Proteins LC3B and GABARAP. *bioRxiv* (2024).

33. Kabsch, W. XDS. *Acta Crystallogr. D Biol. Crystallogr.* **66**, 125–132 (2010).

34. Vagin, A. & Teplyakov, A. Molecular replacement with MOLREP. *Acta Crystallogr. D Biol. Crystallogr.* **66**, 22–25 (2010).

35. Vagin, A. & Lebedev, A. MoRDa, an automatic molecular replacement pipeline. *Acta Crystallogr. A Found. Adv.* **71**, s19–s19 (2015).

36. Terwilliger, T. C. *et al.* Iterative model building, structure refinement and density modification with the PHENIX AutoBuild wizard. *Acta Crystallogr. D Biol. Crystallogr.* **64**, 61–69 (2008).

37. Liebschner, D. *et al.* Macromolecular structure determination using X-rays, neutrons and electrons: recent developments in Phenix. *Acta Crystallogr. D Struct. Biol.* **75**, 861–877 (2019).

38. Emsley, P., Lohkamp, B., Scott, W. G. & Cowtan, K. Features and development of coot. *Acta Crystallogr. D Biol. Crystallogr.* **66**, 486–501 (2010).

39. Williams, C. J. *et al.* MolProbity: More and better reference data for improved all-atom structure validation. *Protein Sci.* **27**, 293–315 (2018).

40. Winn, M. D. *et al.* Overview of the CCP4 suite and current developments. *Acta Crystallogr. D Biol. Crystallogr.* **67**, 235–242 (2011).

41. McCoy, A. J. *et al.* Phaser crystallographic software. *J. Appl. Crystallogr.* **40**, 658–674 (2007).
